# The Transcriptional Regulator CBP Has Defined Spatial Associations within Interphase Nuclei

**DOI:** 10.1371/journal.pcbi.0020139

**Published:** 2006-10-20

**Authors:** Kirk J McManus, David A Stephens, Niall M Adams, Suhail A Islam, Paul S Freemont, Michael J Hendzel

**Affiliations:** 1 Department of Oncology, Cross Cancer Institute, Edmonton, Alberta, Canada; 2 Department of Mathematics, Imperial College London, London, United Kingdom; 3 Division of Molecular Biosciences, Imperial College London, London, United Kingdom; State University of New York at Buffalo, United States of America

## Abstract

It is becoming increasingly clear that nuclear macromolecules and macromolecular complexes are compartmentalized through binding interactions into an apparent three-dimensionally ordered structure. This ordering, however, does not appear to be deterministic to the extent that chromatin and nonchromatin structures maintain a strict 3-D arrangement. Rather, spatial ordering within the cell nucleus appears to conform to stochastic rather than deterministic spatial relationships. The stochastic nature of organization becomes particularly problematic when any attempt is made to describe the spatial relationship between proteins involved in the regulation of the genome. The CREB–binding protein (CBP) is one such transcriptional regulator that, when visualised by confocal microscopy, reveals a highly punctate staining pattern comprising several hundred individual foci distributed within the nuclear volume. Markers for euchromatic sequences have similar patterns. Surprisingly, in most cases, the predicted one-to-one relationship between transcription factor and chromatin sequence is not observed. Consequently, to understand whether spatial relationships that are not coincident are nonrandom and potentially biologically important, it is necessary to develop statistical approaches. In this study, we report on the development of such an approach and apply it to understanding the role of CBP in mediating chromatin modification and transcriptional regulation. We have used nearest-neighbor distance measurements and probability analyses to study the spatial relationship between CBP and other nuclear subcompartments enriched in transcription factors, chromatin, and splicing factors. Our results demonstrate that CBP has an order of spatial association with other nuclear subcompartments. We observe closer associations between CBP and RNA polymerase II–enriched foci and SC35 speckles than nascent RNA or specific acetylated histones. Furthermore, we find that CBP has a significantly higher probability of being close to its known in vivo substrate histone H4 lysine 5 compared with the closely related H4 lysine 12. This study demonstrates that complex relationships not described by colocalization exist in the interphase nucleus and can be characterized and quantified. The subnuclear distribution of CBP is difficult to reconcile with a model where chromatin organization is the sole determinant of the nuclear organization of proteins that regulate transcription but is consistent with a close link between spatial associations and nuclear functions.

## Synopsis

The cell nucleus is the part of the cell that houses the genome and the associated machinery that are responsible for its duplication, maintenance, and expression. It has become apparent that the individual chromosomes that comprise the genome and the machinery that act on the genome and its RNA products are organized within the nuclear volume. The nature of this organization has been difficult to define because simple mapping has shown that it is not defined by predefined 3-D locations for each component. In this study, McManus and colleagues have developed a statistical tool to facilitate the characterization of spatial relationships, their relationship between organization and function, and the identification of rules defining these relationships. With the specific example of the CREB–binding protein, the authors have used this new statistical tool to determine how the organization of the CREB–binding protein relates to the varying protein–protein complexes, catalytic activity, and functions of the protein. Their results demonstrate that this statistical approach can identify spatial relationships that cannot be defined by the more simple techniques employed to date and can open the door for determining the rules of nuclear organization.

## Introduction

It is now appreciated that the spatial relationships between chromatin and nonchromatin structures within the nucleoplasm are correlated with transcriptional activity. Some general rules are emerging for the organization of chromatin that are typically cited as evidence for both spatio–temporal organization of the nucleoplasm and for an underlying regulated process to establish and maintain spatio–temporal organization [1,2]. Specifically, chromosomes and regions of chromosomes segregate differently within the nucleus, depending on whether or not they are rich in potentially transcribed genes. This organization has been described as a polar chromosomal organization because the individual interphase chromosome territories segregate their R-bands (gene rich) into the interior of the nucleoplasm, whereas their G-bands (gene poor) are gathered against the periphery of the nucleus and against the nucleolar surface [3]. Euchromatin sequences are further organized such that they maintain a spatial relationship with the predominant nucleoplasmic nonchromatin structure, the splicing factor compartments [4]. Smaller nonchromatin structures, such as promyelocytic leukemia (PML) and Cajal bodies, associate with specific regions of the genome [5–7]. Beyond these rather general descriptors, our understanding of spatio–temporal regulation of the genome is limited. Most important, the most obvious prediction that arises from the molecular characterization of the RNA polymerase II (RNA PolII) transcriptional machinery, that genes represent the principal nuclear binding site for these proteins, has not been commonly observed despite obvious attention to the question [8,9].

Careful studies have been performed with 3-D deconvolution microscopy or laser scanning confocal microscopy to examine the spatial relationships between RNA PolII, sites of RNA PolII transcription, sequence-specific DNA binding proteins, and chromatin modifying machinery [8–11]. For example, Grande et al. [9] examined the distribution of the glucocorticoid receptor, Oct1, and E2F-1 relative to RNA PolII and found little or no relationship. Similarly, Hendzel et al. [12] examined the relationship between transcription factors involved in chromatin modification and modified chromatin. They concluded that chromatin was not defining the subnuclear localization of these proteins. Coimmunoprecipitation also does not dictate that molecules colocalize. For example, a study of hnRNPs demonstrated to coimmunoprecipitate also failed to find that these proteins colocalize [13]. These proteins are consistently observed to enrich in small but abundant foci that are distinct from the larger, less-abundant foci that are commonly observed when transcription factors are transiently overexpressed. The latter often do show colocalization of nuclear proteins that can interact [14,15]. The failure of the native small nuclear foci to colocalize has led us to propose that these may be structures that are independent of chromatin, perhaps involved in the assembly of macromolecular complexes rather than reflecting sites where they function [14]. Consistent with this hypothesis, foci enriched in the transcription factors Sp1 and Sp3 have recently been shown to persist through mitosis, where they are found in the cytoplasm and associate with F-actin rather than chromatin [16].

By performing a comprehensive localization analysis for the transcriptional regulator CREB–binding protein (CBP), we have revealed that, while there may be rules for the spatial organization of these foci, they are not easily linked to function by colocalizing to sites of known activities. An alternative relationship that appears to be related to function has been defined for nuclear structures involved in the expression of protein-coding genes. Structures may show clear spatial relationships that are complimentary, rather than coincident. The best characterized of these relationships include the proximity of the major histocompatibility complex gene cluster to PML bodies [5] and the association of transcribed genes to splicing factor compartments [4].

If we accept the alternative possibility for intranuclear sites enriched in proteins involved in transcriptional regulation of RNA PolII–transcribed genes [14], spatial relationships rather than spatial colocalization may be an important factor in terms of function. This possibility has been introduced previously when it was determined that nuclear bodies that are related in function are often in close spatial proximity within the nucleoplasm [17]. When examined by 3-D deconvolution microscopy, protein-rich intranuclear sites involved in RNA PolII transcription typically approach the resolution limits of the microscope (~200 nm diameter). Because of their small size, we cannot be sure whether they represent single unified structures or a number of smaller sites packed close together. We can, however, make estimations about the number of macromolecules present within the structure and thus stochiometric relationships in colocalization studies. In addition, we know with certainty that these foci do not contain one or two copies of the protein under study, but comprise hundreds to thousands of individual macromolecules [18]. In this context, the failure to observe coenrichment of chromatin proteins or DNA in these foci may reflect separate functions for these structures. We have previously proposed that such intranuclear foci are involved in processes such as the assembly of multiprotein complexes that can then be released into the surrounding nucleoplasm where they can act directly on their chromatin target [14,19].

In this study, we have extended our earlier studies to determine whether CBP-enriched nuclear foci have distinct nonrandom spatial associations within the interphase nucleus. CBP is a well-defined and important regulator of gene transcription and chromatin structure and has measurable histone acetyltransferase (HAT) activity in vivo, with a strong preference for specific lysines (Lys) on histones H3 and H4 [18]. Furthermore, CBP is a well-established transcriptional coactivator for a large number of DNA binding proteins and is able to associate with an ever-expanding list of nonhistone nuclear proteins. This leads to the attractive hypothesis that CBP may function as a molecular scaffold that associates directly or indirectly with a variety of proteins simultaneously, including components of the transcriptional machinery and specific histone susbstrates [19]. To test this hypothesis, we have developed a distance-based statistical method that can analyse relationships between intranuclear foci in terms of interpoint distances (e.g., [20]). This approach is particularly appropriate since no other useful localizing information is readily available, the measurements relate to real distances, and the coordinate system used to calculate distances is essentially arbitrary, thereby allowing meaningful comparisons between individual cells and different batches of cells. Furthermore, there are a number of available methods for summarizing information from the collection of observed distances, including nearest-neighbor (NN) distances and mean distance to all points; for example, Noordmans et al. analyze voxel-by-voxel data across the entire 3-D image to gain understanding of the spatial heterogeneity in signal [21]. In contrast, our statistical approach focuses only on the point pattern corresponding to the identified objects in the image. Using this approach, we find that CBP has a hierarchy of nonrandom spatial relationships with a subset of nuclear compartments, including RNA PolII transcription components and chromatin. We also find that CBP has a significantly higher probability of being spatially associated with its known in vivo substrate, histone H4 Lys 5, compared with the closely related H4 Lys 12. Our results have broader implications in the context of understanding nuclear organisation, where the underlying spatial mechanisms are unknown.

## Results

### Characterization of CBP Distribution Relative to Bulk Chromatin

**Figure 1:**
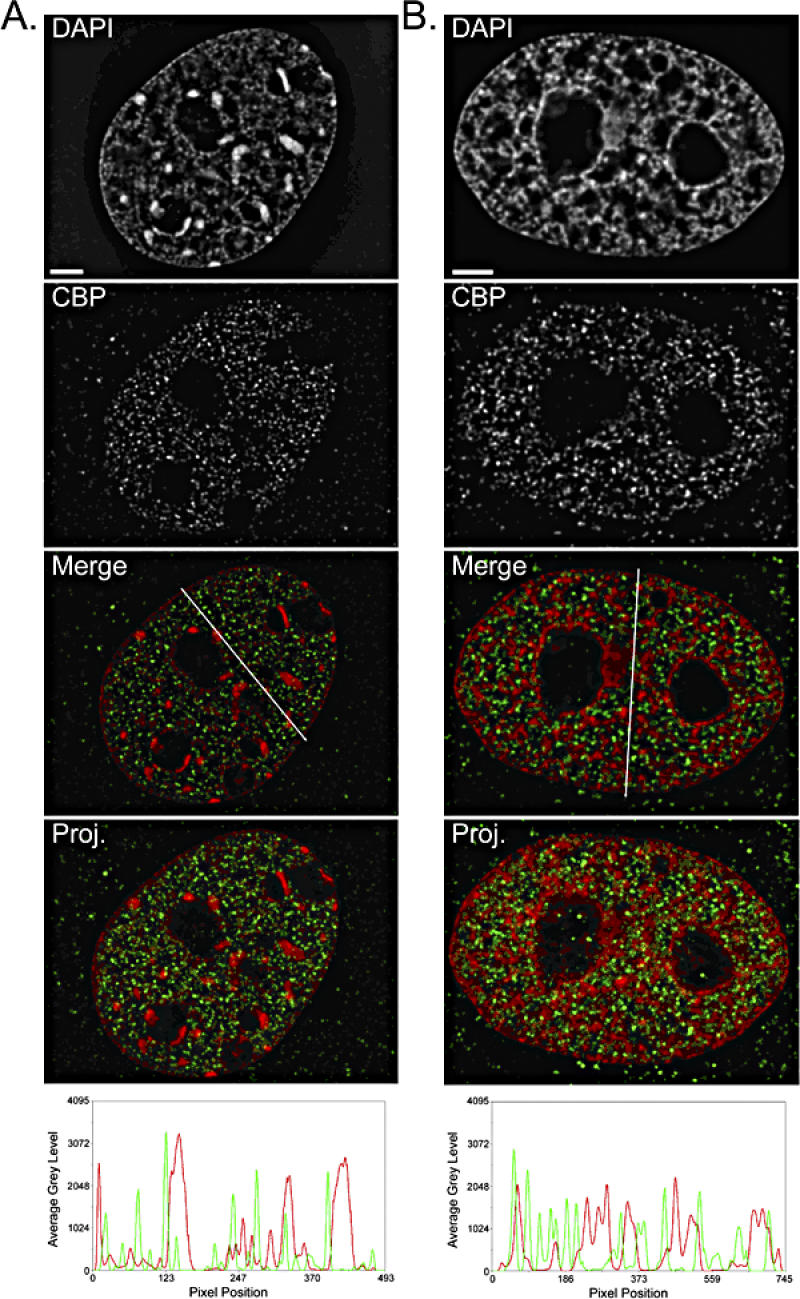
Spatial Relationship between Endogenous CBP and DNA Shown here are representative high-resolution (100×) deconvolved images of an interphase 10T1/2 cell (A) and an IM cell (B) immunofluorescently labeled with anti-CBP and counterstained with DAPI. A combined image of the single planes is presented as the “Merge,” with the CBP and DNA shown in green and red, respectively. A 3-D projection of the entire nucleus is presented (Proj). The linescan (bottom row) demonstrates that endogenous CBP preferentially localizes within euchromatin (less intense DAPI signal) or in regions immediately adjacent to intensely staining heterochromatic regions (intense DAPI signal). Scale bars represent 3 μm.

To define the properties of nuclear foci enriched in RNA PolII transcription factors, we determined whether or not CBP foci colocalized with reference structures of differing function. Our analysis focused on determining whether or not CBP foci colocalized with euchromatin, sites of dynamic acetylation, sites of RNA PolII transcription, and heterochromatin within the nucleoplasm. Figure 1 shows the relationship between CBP and the distribution of genomic DNA. The deconvolved images on the left show the relationship between the DNA distribution (red in composite images) and CBP (green in composites) in mouse 10T1/2 embryonic fibroblast cells, while those on the right depict the relationship between DNA distribution and CBP distribution in Indian muntjac fibroblast cells. The colour panels show projected 3-D images. Unlike the DNA distribution, CBP is found to concentrate in several hundred small foci. We have previously shown that enhanced green fluorescent protein–tagged CBP also enriches in small nuclear foci [18,19]. Hence, this distribution is not a consequence of fixation but reflects the endogenous distribution of CBP. When line scans are used to compare the concentration of DNA with the concentration of CBP, CBP is predominantly found in chromatin-depleted regions.

### Characterization of CBP Distribution Relative to Sites of Histone Modifications that Demarcate Euchromatin

**Figure 2:**
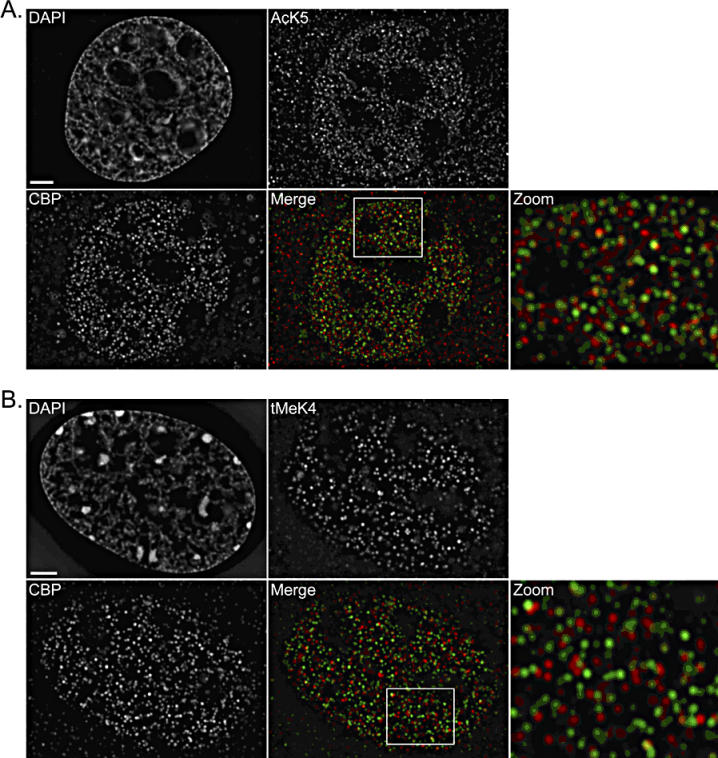
Spatial Relationship between CBP and Specific Histone H3 Post-Translational Modifications The spatial relationship between CBP and a known product of its HAT activity, acetylated K5 (H4) (A), or an unrelated histone H3 modification, trimethylated K4 (H3) (B), was investigated. Representative and deconvolved high-resolution images (100×) of interphase 10T1/2 cells immunofluorescently labeled with anti-CBP and either anti-AcK9 or anti-tMeK4 counterstained with DAPI are presented. The merged image presents a single plane from the 3-D projection where CBP and AcK9 or tMeK4 are shown in green and red, respectively. A region, identified by the white box, is further magnified to show the spatial relationship between CBP and either post-translational histone modification. Scale bars represent 3 μm.

We have previously observed that histone modifications, such as highly acetylated histone H3, are also found in small nuclear foci that enrich in chromatin-depleted regions of the nucleus. Therefore, one potential explanation for this distribution is that it reflects the distribution of transcriptionally active or potentiated regions of the genome (i.e., euchromatic). We have also shown that cells expressing CBP preferentially show increases in Lys 5 acetylation of histone H4 [18]. Hence, our results implicated CBP as a histone H4 K5 acetyltransferase. Because this modification has a high turnover rate, continuous HAT activity is required to maintain K5 acetylation in these regions of the genome. Consequently, we expected that CBP would show a high degree of colocalization with acetylated K5 in these regions. To address this, we performed colocalization experiments to determine whether CBP was associated with regions that were enriched in either acetylated histone species or an unrelated control, trimethylated Lys 4 (tMeK4) of histone H3. Both histone modifications have been reported to be associated with transcriptionally active/competent regions of the genome. Figure 2A depicts deconvolved images obtained from these experiments. As expected, both antibodies show preferential staining of nuclei, and, as with CBP, are enriched in several hundred small nuclear foci. Surprisingly, when CBP images (green in composites) are compared with either acetylated K5 of histone H4 (red in composites), there are very few examples where these foci colocalize—very few foci show the presence of both red and green signals (yellow in composite). Rather, most labeled regions of the nucleoplasm contain either red or green foci, but not both. This is particularly evident in the images/regions presented at higher magnification.

Although we expected CBP to colocalize with K5, it is possible that K5 sites are already fully acetylated at the time of fixation, which would negate any CBP clocalization at these sites. Nonetheless, we expected that CBP would be associated with transcriptionally active/competent regions of the genome, thereby providing the basis for its focal distribution. To further address this possibility, we compared the distribution of CBP with tMeK4 of histone H3. This modification has consistently been found enriched in transcriptionally active regions of the genome [22,23]. Figure 2B compares the distribution of CBP (green in composites) with tMeK4 (red in composites). As expected, both epitopes are concentrated in small nuclear foci. When the distribution of CBP was compared with this modified histone species, however, the results once again revealed very little evidence of colocalization. Instead, these proteins appeared to be enriched in foci that were distinct from each other. Figure S1 shows an example of colocalizing proteins and verifies that the failure to observe colocalization is not due to optical misalignment.

### CBP Distribution Relative to Sites of Transcription

**Figure 3:**
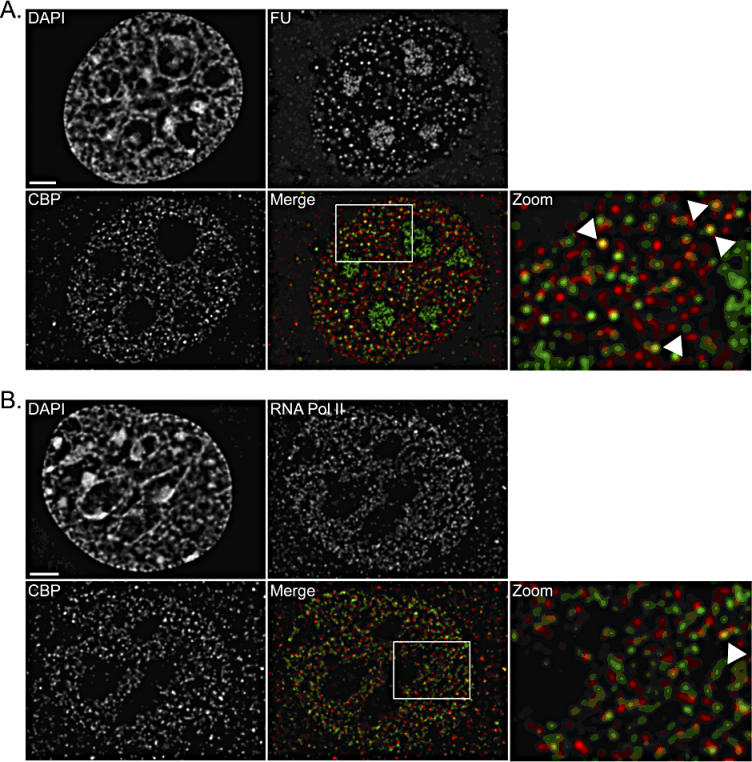
Spatial Relationship between CBP and Transcription The spatial relationship between CBP and transcription, as identified by FU incorporation in nascent RNA transcripts (A) or immunofluorescent labeling of RNA PolII (8WG16) (B), was investigated. Representative and deconvolved high-resolution images (100×) of interphase 10T1/2 cells immunofluorescently labeled with anti-CBP and either anti-FU or anti-8WG16 counterstained with DAPI are presented. The merged image presents a single plane from the 3-D projection where CBP and FU or 8WG16 are shown in green and red, respectively. A region, identified by the white box, is further magnified to show the spatial relationship between CBP and either post-translational histone modification. Scale bars represent 3 μm.

While the colocalization experiments with modified histone species indicated that CBP localization into small nuclear foci was not determined by euchromatin organization, it may be that the association of CBP with chromatin masks the histone epitopes that we used as markers for transcriptionally active chromatin. Therefore, we again tested whether or not CBP was predominantly associated with transcriptionally active regions of the genome. In this instance, however, we used antibodies recognizing a halogenated nucleotide incorporated into nascently synthesized RNA (Figure 3A) using a brief pulse labeling with fluorouridine or an antibody recognizing RNA PolII (Figure 3B). While we observed examples of colocalization at the level of resolution of the fluorescence microscope (see arrows in Figures 3A and 3B), once again the majority of the CBP foci (red in composite images) existed in regions that were independent of the presence of newly synthesized RNA (green in Figure 3A composite) or RNA PolII (green in Figure 3B composite).

### CBP Distribution Relative to CBP-Associated Proteins

**Figure 4:**
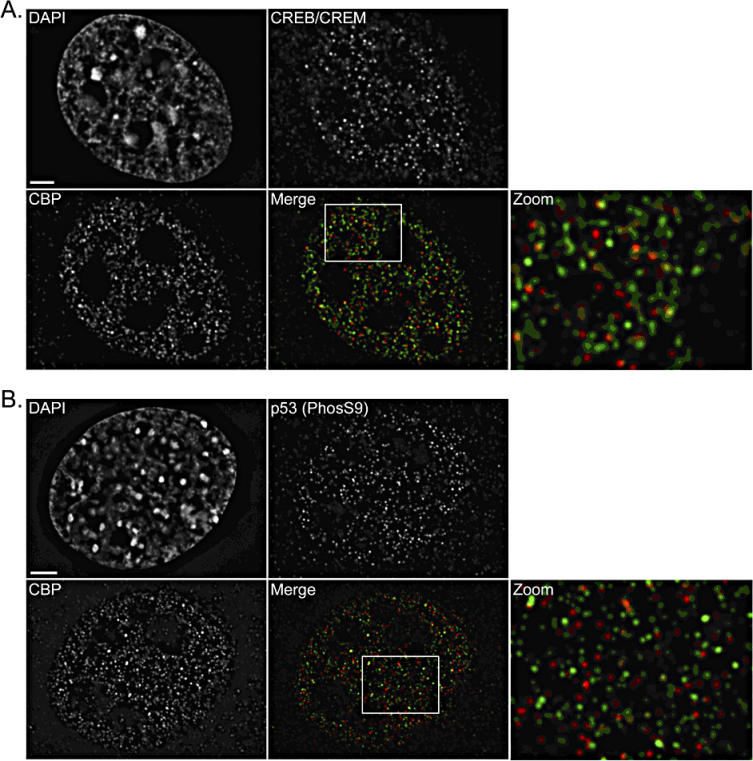
Spatial Relationship between CBP and Regulators of Transcription The spatial relationship between CBP and CREB/CREM (A) and phosphorylated serine 9 of p53 (B) was investigated. Representative and deconvolved high-resolution images (100×) of interphase 10T1/2 cells immunofluorescently labeled with anti-CBP and either anti-CREB/CREM or anti-p53(phosS9) counterstained with DAPI are presented. The merged image presents a single plane from the 3-D projection where CBP and CREB/CREM or p53(phosS9) are shown in green and red, respectively. A region, identified by the white box, is further magnified to show the spatial relationship between CBP and either post-translational histone modification. Scale bars represent 3 μm.

While the results above are not consistent with a euchromatin- or transcription-dependent basis to the organization of CBP foci, proteins have been demonstrated to colocalize within these foci. For example, we have previously demonstrated that histone deacetylase-3 (HDAC3) and HDAC4 colocalize at the level of individual foci [15]. It is possible, therefore, that rather than reflect nuclear sites where these proteins carry out their activities, these nuclear foci may be involved in the assembly of multimolecular complexes that are then available to function in the local environment. To address this, we examined the distribution of CBP relative to proteins that CBP has been previously shown to directly interact with through coimmunoprecipitation experiments. Figure 4 shows examples. Once again, although there were instances of colocalization, these results differed significantly from our previous experiments with HDAC3 and HDAC4, where their subnuclear distributions were very close to identical.

### Statistical Approaches for Defining CBP Spatial Organization

To test the hypothesis that CBP is in spatial proximity to the chromatin that it acetylates, we characterized the spatial relationships between CBP foci and a series of other nuclear foci, including sites enriched in newly synthesized RNA, RNA PolII, p53, CREB, and acetylated histones in the mouse 10T cell line. We were particularly interested in defining the relationship with chromatin enriched in histone H4 acetylated at K5. Lys 5 is the least abundant acetylated species of histone H4 and is a product of CBP acetylation in vivo [18]. In our approach, we extracted Cartesian coordinates for the centroid positions of individual foci and used simple interpoint distances and NN methods, including probability measures (see Table 1 for definition of terminology). Although our approach does not take into account the irregular shape of the nucleus (for individual cells and between cells) or potential exclusion regions (e.g., nucleoli), it does have the advantage that the data can be normalized for nuclear volume across different cell images, allowing multiple observations from different cells to be used in the analysis. Furthermore, the numbers of observed CBP and non-CBP (NCBP) foci are sufficiently large (typically 100–300) and generally equivalent to provide statistically meaningful comparisons.

**Table 1:**
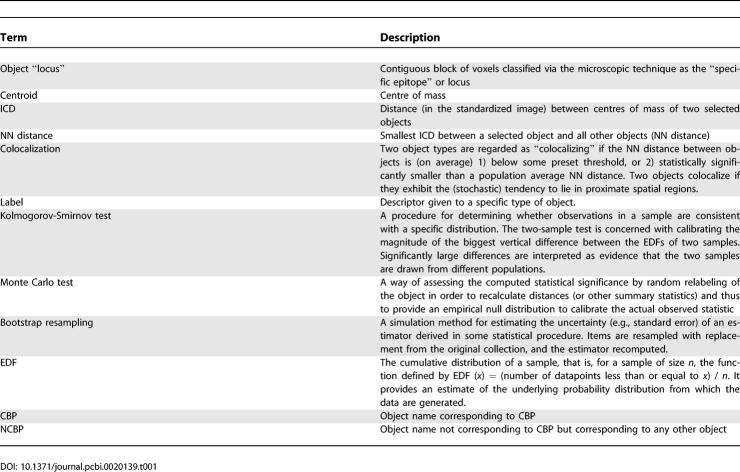
Description of Statistical Terminology

**Figure 5:**
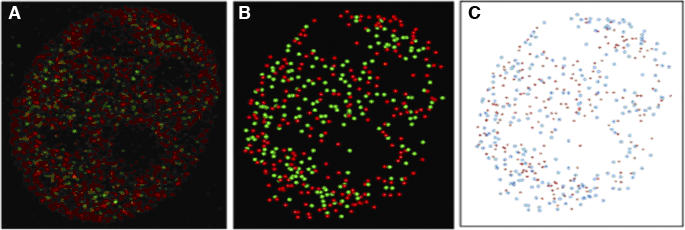
Spatial Analysis of CBP and Acetylated Histones in Preserved 3-D Mouse 10T Fibroblast Nuclei Projected images of CBP (green) and acetylated histone K12 (red) were collected by widefield fluorescence microscopy in 200-nm z-steps followed by deconvolution. (A) The deconvoluted images were processed using the program Image3dV (http://www.sbg.bio.ic.ac.uk/people/suhail/suhail.html) to obtain centroid positions of each fluorescent foci and displayed using the graphics program PREPI (http://www.sbg.bio.ic.ac.uk/prepi). (B) A scatterplot of the extracted centroid positions showing good agreement with the original projected confocal image (C).

The first step of our analyses involves extracting centroid coordinates *(X, Y, Z)* for CBP and NCBP foci from deconvoluted widefield fluorescence images. To do this we carried out a simple image reconstruction procedure of the image data, which essentially involved two steps, namely isosurface generation and foci identification via surface tracking (see Materials and Methods). We used a Marching Cubes technique [24] to construct our isosurfaces, using a number of threshold values to reproduce the characteristics of the 3-D image stack. Using these surface/volume reconstructions, we then identified the centers of individual 3-D volumes (centroids) with a surface-tracking connectivity algorithm and assigned these *X, Y, Z* coordinates using an arbitrary axes system. Figure 5 shows a comparison between an original image (stacked projection) and the extracted 3-D–centroid points in projection showing good agreement with the original image and validating our reconstruction procedure. Each individual point represents the centroid point of the volume attributed to individual CBP-enriched foci and acetylated histone K12–enriched foci shown as a scatterplot (Figure 5). Other approaches for confocal image reconstruction and spot detection have also been reported [21]. The next stage of our approach was to compute NN distances between CBP and NCBP foci. We then carried out pairwise comparisons of these NN distance distributions. Our analysis uses the two-sample Kolmogorov-Smirnov test for point-wise equality of distribution functions (for example, see [25]). This test is preferable to the simpler Mann-Whitney two-sample test for equality of medians, as it is more powerful for detecting small differences underlying distributions, although the Mann-Whitney test is a feasible alternative. To account for nuclei sizes, all distance measurements are on a standardized scale relative to the maximum interobject distance within a nucleus.

**Figure 6:**
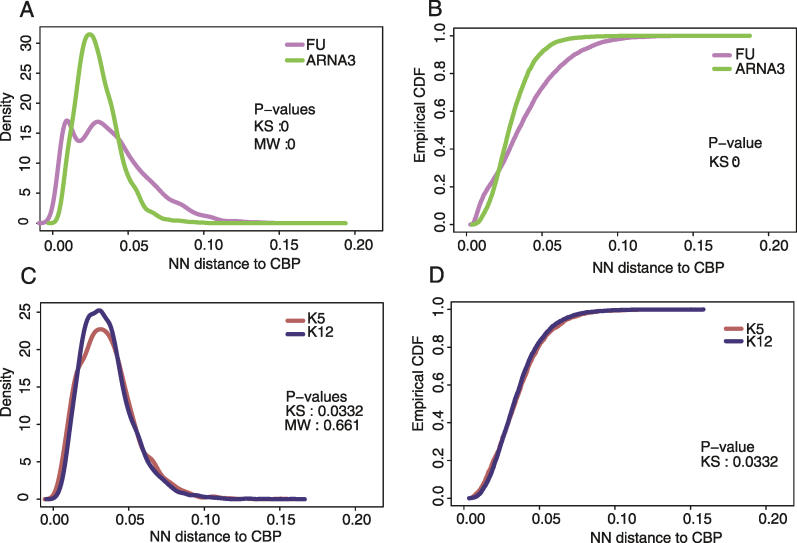
Distribution Plots of NN Distances for NCBP Foci to CBP EDF and density plots are shown for pairwise comparison of NN distances (see text and Table 2 for details). The density plot represents density estimates of the NN distance distribution (NCBP to CBP) for nuclear components taken pairwise. KS is the *p-*value in the Kolmogorov-Smirnov test, while MW refers to the Mann-Whitney statistic. (A) EDF plot comparing acetylated Lys5 (K5) and Lys12 (K12) of histone H4. No statistical difference is observed, suggesting that CBP is as close to both K5 and K12 foci. (B) Density distribution plot for the same pairwise comparisons showing a similar trend as in (A). (C) EDF plot comparing NN distances for CBP to FU and ARNA3. The FU foci distribution is significantly different from the ARNA3 foci, suggesting that CBP is closer to PolII than to nascent mRNA transcripts. Note the shape of the FU distribution at smaller CBP NN distances, indicating that a subpopulation of FU foci lie close to CBP foci. (D) Density distribution plot for the same comparison. Note the bimodal distribution for FU, indicating a population of FU foci that lie closer to CBP than active PolII and could represent sites of active transcription.

Examples of these pairwise comparisons are given in Figure 6 and Figure S1. To assess the validity of our approach in dealing with multiple observations from different cells, we compared CBP–CBP distances from different batches of 10T fibroblasts and found no significant difference between these distributions, which supports the validity of our approach (unpublished data). Figure 6A graphically presents the NN distance distributions for acetylated K5 of histone H4 with acetylated K12 of histone H4. Both distributions are remarkably similar with no significant differences observed, suggesting that CBP is as close to sites enriched in both acetylated lysines. In contrast, a comparison between the NN distance-based distributions of 5-fluro-uridine (FU), identifying nascent mRNA transcripts and active RNA PolII (ARNA3) shows significant differences (Figure 6C). CBP is on average closer to sites enriched in active RNA PolII than sites enriched in nascent transcripts. However, for all the FU pairwise comparisons we did note a small sample of close association to CBP (Figure 6D), which could reflect sites of active transcription.

**Table 2:**
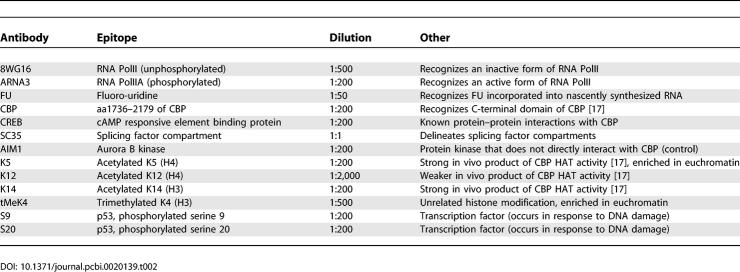
Description of Antibodies and Epitopes

**Figure 7:**
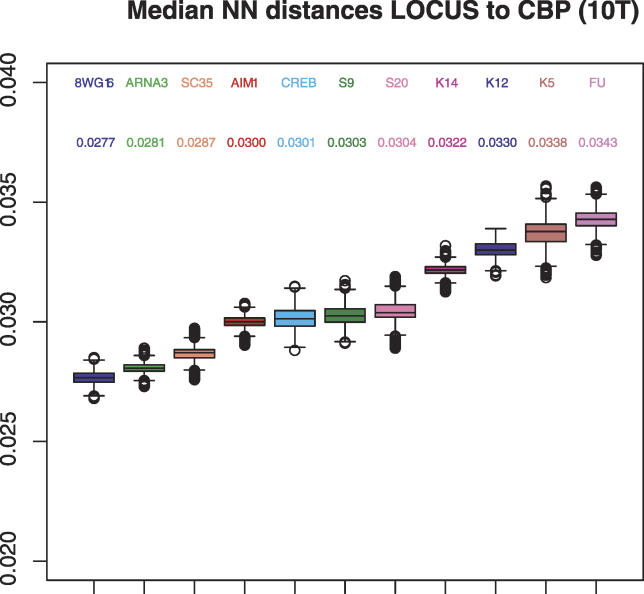
Boxplot of Median NN Distances for Different Nuclear Components to CBP Foci The vertical axis represents relative distances to the maximal possible distance in microns, with the median distances for each focus highlighted. The box represents the interquartile range of these distances. The “whiskers” indicate the 5% and 95% quantiles, and distances observed outside this range are indicated with circles. The nuclear foci analysed for NN distances to CBP are: 8WG16, RNA PolII (hypophosphorylated); ARNA3, PolII (hyperphosphorylated); SC35, pre-mRNA splicing speckles; AIM1, Aurora B kinase; CREB, Creb/Crem transcriptional activator; S9, p53 phosphorylated Ser9; S20, p53 phosphorylated Ser20; K14, acetylated Lys14 histone H4; K12, acetylated Lys12 histone H4; K5, acetylated Lys5 histone H4; FU, fluoro-uridine.

From our NN distance distributions, we could compute median distances as a way of comparing associations of sites enriched in CBP with sites enriched in other NCBP components (Figure 7). These distances can be interpreted as estimated median NN distances with defined uncertainty intervals for each comparison; here the 95% confidence intervals for the median distance were estimated using bootstrap resampling. This general type of procedure has been much used in many fields of application, including biology (for example, see [26]), and involves random relabeling of objects, and recalculation of the test statistic, for a large number of bootstrap resamples, in order to gain an understanding of the variability of the statistic. For example, if an image contains N = N0 + N1 objects, with *N0* labeled 0 (CBP) and *N1* labeled 1 (nuclear component), we may compute a summary statistic *T* that is informative about the positive or negative spatial association between object types. We used the median interobject distance between the nuclear component and CBP computed over all images in the experiment. Suppose that, for the original data, the summary statistic is observed to be *T* = t*. To obtain a standard error or uncertainty interval for the statistic, we formed a pseudo-dataset by resampling with replacement *N0* objects from the list of objects labeled 0, and then inspected the NN distances for this new set of pseudodata to form a new pseudo–summary statistic, *t_1_.* We then repeated this exercise B times to form a sample of pseudostatistics *t_1_*,…, *t_B_,* and reported the standard error (or 95% central range) derived from this sample as the estimated uncertainty measure for the summary statistic concerned. A similar method, in a similar application, was used by Knowles et al. [27]; see also the discussion below.

The results in Figure 7 are shown as boxplots with median NN distance to CBP for different nuclear components shown in ascending order. The scale of these are relative to the maximal possible distance measured across all images and do not reflect actual distances; this standardization is necessary as the images were produced at different magnifications. Interestingly, we observe three apparent groupings with one group of “closer” CBP associations comprising active/inactive PolII and SC35 pre-mRNA splicing sites. A middle grouping comprises known and putative CBP binding proteins, including CREB and phosphorylated p53 (Ser9 and Ser20). Surprisingly, the final group which shows higher median NN distances and therefore less “close” associations, comprises the acetylated histones H4 (Lys 5, Lys 12, and Lys 14) and sites of nascent mRNA transcripts.

### Comparison of Different CBP–NCBP Spatial Distributions

**Figure 8:**
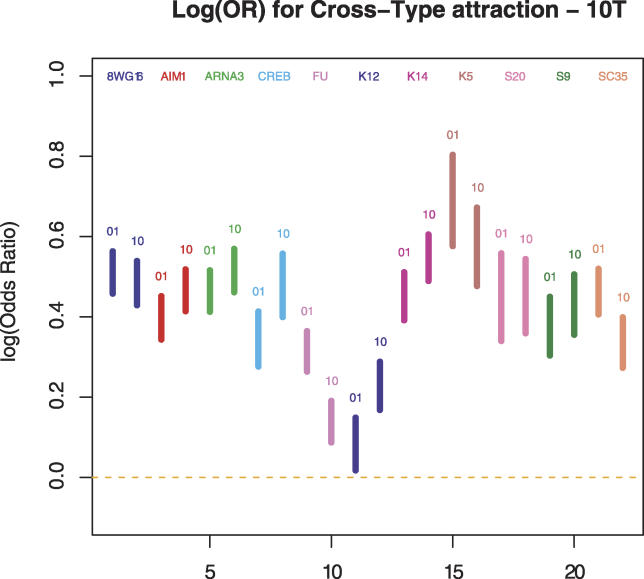
Plot of “Attraction” or Association of Different Nuclear Components with CBP Foci The vertical axis represents the log of the excess odds ratio over randomness, with a value of zero equivalent to random association. The different nuclear body components are labeled as in Figure 3. NCBP “association” to CBP is labeled 01, with CBP “association” to NCBP labeled 10. The bars represent a 95% confidence interval for the mean association (see text for further details). Note the significant difference in “probability of association” between K5 and K12.

To compare quantitatively between different CBP and NCBP associations as well as between different cells and cell lines, we used a simple probability model to determine whether the NN to a CBP is not a CBP body, taking into account the number of both CBPs and NCBPs (see Materials and Methods). If we observed a large value for the probability that the NN to a CBP focus is not a CBP, or vice versa, this suggested that CBP exhibits a spatial “attraction” (association) to the other focal type. By comparing these probability values, we indirectly measured the strengths of “attraction” between CBPs and other nuclear components, the results of which are shown in Figure 8. The strengths of “attraction” (association) are quantified as an excess odds over random between CBP and NCBP (labeled 01) or NCBP to CBP (labeled 10). Specifically, we used a binary generalized linear model (see, for example, [28]), with the response variable being the number of 01 associations, which is modeled as a binomial random variable. For example, suppose there are N0 CBPs, where N01 of them have an NCBP as NNs, and N00 have a CBP as an NN. We modeled N01 ~ binomial (N0, p01), where *p01* is the probability that a randomly selected CBP has an NCBP as an NN. To account for the imbalance in the numbers of CBP/NCBP for different images and nuclear components, we utilized an offset model. That is, a hypothesis of “no association” might naively be thought to correspond to p01 = 0.5, but if, say, there is 60/40 majority of CBPs in an image series, then if there was no association between CBP and NCBP, we would obtain an estimate of p01 near to 40/100 = 0.4, and might infer a negative association which is not supported in reality. The offset model uses the following formula for p01:

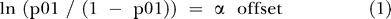
where offset = ln (N01/N00). An estimate of α reveals the nature of the association; α> implies positive association, and α< implies negative association.

In the plots, the zero point of the vertical axis corresponds to a random spatial association. Values above this point indicate an association stronger than expected by chance, and is therefore indicative of association. The figure also includes 95% confidence intervals for each probability. Interestingly, all NCBP foci show differing strengths of CBP association above random apart from acetylated K12 of histone H4 and to some extent, FU. In contrast, the highest probability for CBP association is exhibited by acetylated K5 of histone H4. This is of particular significance given that the boxplots of median distances between K5 and K12 are similar (Figure 7), as are their NN distance distributions (Figure 6A).

## Discussion

In previous studies, we have demonstrated a strong correlation between CBP expression and the amount of histone H4 acetylated at Lys 5 [18]. Lys 5 is the last acetylation site used on histone H4 and, consequently, is the least abundant of histone H4 acetylations found primarily in the tetra-acetylated form of H4 [29–31]. The linear relationship between CBP expression and the amount of nuclear acetylated Lys 5 [18] and the rapid turnover of this acetylated species of histone H4 [32] prompted us to examine whether CBP was specifically enriched in the same regions of chromatin that are acetylated at Lys 5. Surprisingly, we found that this was not the case. Rather, foci enriched in CBP appear to exist independently of the chromatin that it acetylates, which is consistent with the hypothesis that HATs exist in structures that are independent of their chromatin binding sites [14,33,34], perhaps as a mechanism to facilitate the assembly of chromatin-modifying complexes. However, it is worth noting that there is no consensus for the functional significance of CBP or other nuclear foci, which are often found as aggregates associated with an insoluble nuclear fraction. It has been argued that such aggregates may act as storage domains or sites for complex macromolecular assembly or even modifications (reviewed in [30]). Furthermore, it has been shown that the majority of components involved in gene expression are dynamic, with potentially only a small fraction of the total pool of factors active at any one time (reviewed in [31]). Despite these caveats, little is known about the overall spatial relationships of nuclear foci and, in particular, CBP foci. We wanted to test the hypothesis that CBP foci occur preferentially near sites of CBP activity and find out whether there existed a hierarchy of spatial relationships between CBP foci and other nuclear foci involved in transcription and histone modification.

To examine this, we analysed the spatial associations of foci enriched in CBP within the mouse 10T cell line with components of the transcriptional machinery as well as sites of specific chromatin modification. Using a new statistical approach, we find that CBP has a hierarchy of nonrandom spatial relationships with a subset of nuclear compartments maintaining a nonrandom spatial proximity to the chromatin that it preferentially acetylates in vivo. Of particular interest is the comparison of CBP proximity to foci enriched in acetylated H4 Lys 12 with acetylated H4 Lys 5. In terms of NN distance measurements, CBP appears equally close to both sites. However, what is surprising is that when a probability measure is made on the likelihood of being proximal to either site, a striking difference is observed. We find that CBP has a significantly greater chance of being close to acetylated H4 Lys 5 than Lys 12, despite acetylated Lys 12 being more abundant within the genome. We also examined the spatial relationships with a number of additional proteins, including RNA PolII species, sites of RNA transcription, phosphorylated species of p53, and CREB. Each of these nuclear targets are expected to colocalize with CBP if the foci enriched in CBP merely reflect sites of chromatin association rather than nuclear structures that are independent of sites of action within chromatin. Interestingly, with the exception of K12 acetylation, all of the studied examples of nuclear proteins exhibit spatial relationships that are higher than expected from randomly arranged structures. In addition, there are varying degrees of “affinity” between the NCBP foci and the CBP-enriched foci. From these measurements, there does appear to be a hierarchy of association for CBP foci, which is reflecting an underlying functional organisation. However, these data also suggest for the first time that spatial colocalization within the nucleus may not be a complete measure of functional interdependence, and that a measure of likelihood of association may be more revealing. This is perhaps not surprising given the crowded nature of the interchromosomal space, but it does underlie the need for more quantitative estimates in determining functional associations. Although current methods cannot distinguish between functional relationships that drive spatial arrangements versus underlying organisation leading to specific functional outcomes, this study does provide a first attempt at defining observed spatial arrangements within interphase nuclei. It also provides a foundation for the further study of other well-defined nonchromatin nuclear foci (e.g., PML bodies) and compartments (e.g., chromosomes and nucleoli). Furthermore, our NN distance approach will significantly extend the possibilities of analysing spatial associations on smaller scales that result from improvements in optical imaging techniques.

Developing quantitative methods to understand spatial relationships within interphase nuclei has become an important area of study given that strict colocalization analysis, when applied using the highest resolutions obtainable with fluorescence microscopy, often fail to provide meaningful information. Rather, a number of studies have indicated that spatial proximity rather than spatial colocalization may be important in regulating genome function. For example, highly acetylated chromatin [12], some transcriptionally active genes [35–37], and the gene-rich R-band regions of interphase chromosomes [4] have been reported to be juxtaposed to splicing factor compartments. Similarly, the major histocompatibility complex gene locus maintains a spatial relationship with PML bodies [5], as do active regions of the genome in general [38]. Other patterns of nuclear organisation include preferences for chromosomes to occupy specific regions of the nucleus based on both chromosome size and the transcriptional capacity of the chromatin [1,39–42]. In these studies, radial positioning has been established as a reliable method to demonstrate nonrandom distribution patterns. More recently, relative positioning and chromosome cluster analysis have been used to demonstrate tissue specificity in these patterns [42], and modelling of chromosome territory arrangement has suggested that cell type–specific differences are not due solely to nuclear shape differences [39]. Radial positioning has also been applied to nuclear compartments with more complex distribution patterns (e.g., centromeres [43]). However, the ability to compare distribution patterns for two or more compartments simultaneously requires more sophisticated approaches and tools. Such tools are currently not available, and most researchers tend to rely on either pixel overlap of fluorescent intensities [8,12,44,45] or on cross-correlation analysis [9,13,46,47]. Where spatial proximity has been evaluated, current methods are only able to distinguish random from nonrandom relationships, without being able to quantify and compare multiple spatial relationships [9,48]. In this study we have developed an alternative approach for studying nuclear organisation based on NN distance measurements and probability estimates. Our approach is significantly different from previous studies in that it is object-based, whereas most other studies rely on comparing dual-labeled 3-D images in terms of intensity distributions, with cross-correlation as a measure of overlap compared with random. The advantages of an object-based approach is that it allows some direct spatial measure of specific associations and allows probability estimates of associations between different components given the difficulty in delineating such association in a confined nuclear volume.

In summary, we have studied the spatial associations of the CBP transcriptional regulator within interphase nuclei and have developed statistical approaches for characterizing spatial relationships in terms of distances and probability of association. The clear spatial association of CBP-enriched foci to regions of chromatin that are selectively acetylated by CBP in vivo is consistent with CBP-enriched foci playing a role in targeting the enzyme to specific chromatin substrate sites. To our knowledge, these data provide the first statistical demonstration that spatial proximity rather than spatial overlap defines a functional relationship between an enzyme and chromatin substrate.

## Materials and Methods

### Cell culture.

IM (male Indian Muntjac Skin Fibroblast) and 10T1/2 (C3H mouse embryo fibroblast) cells were cultured in Ham's F10 medium plus 20% fetal bovine serum (FBS) and α-MEM plus 10% FBS, respectively, in a 37 °C incubator with 5% CO_2_. Cells were plated onto sterilized glass coverslips so that they were 50% to 80% confluent on the following day. Subsequent to fixation for 5 min at 23 °C with fresh 4.0% paraformaldehyde, cells were permeablized with phosphate-buffered saline (PBS; pH 7.5) containing 0.5% Triton X-100 for 5 min.

### Immunofluorescent labeling.

Cells were washed twice with PBS and subjected to sequential series of 30-min incubations with appropriate primary and secondary antibodies. Wash steps between incubations were performed consisting of a single wash with PBS containing 0.1% Triton X-100 and two washes with PBS. The primary antibodies used fall roughly into three distinct classes of molecules: those recognizing (modified) DNA/chromatin, RNA, and transcriptional regulators (including transcription factors and coactivators); and they are indicated in Table 2. Primary antibodies were recognized with appropriate mouse or rabbit secondary antibodies conjugated with either Alexa-fluor 488 or Cyanin-3 (Cy-3) (Molecular Probes, http://www.invitrogen.com); and The Jackson ImmunoResearch Laboratories, Inc., http://www.jacksonimmuno.com; respectively). Coverslips were mounted onto slides containing approximately 10 μl of a 90% glycerol-PBS–based medium containing 1 mg/mL parapheylenediamine and 0.5 μg/ml DAPI. 8WG16 and ARNA3 were kindly provided by Dr. Charlotte Spencer (University of Alberta, Edmonton, Alberta, Canada), and the CREB antibody was provided by Dr. Cynthia McMurray (Mayo Clinic, Rochester, Minnesota, United States). The remaining antibodies were commercial and obtained from the following sources: anti-CBP (C-terminal) (Upstate Biotechnology, Millipore, http://www.upstate.com), anti-BrdU (Sigma, http://www.sigmaaldrich.com), SC-35 (ATCC, http://www.atcc.org), AIM1 (BD Biosciences, http://www.bdbiosciences.com), anti-acetylated K5 histone H4 and anti-acetylated K14 histone H3 (Serotec, http://www.serotec.com), anti-acetylated K12 histone H4 (Upstate Biotechnology), and p53 S9 and p53 S20 phospho-specific antibodies (Serotec).

### RNA labeling.

To label nascent transcripts, cycling cells were incubated with 2 mM FU for 20 min. Cells were fixed and permeabilized as indicated above, and nascent transcripts with incorporated FU were identified with an anti-bromodeoxyuridine (Boehringer, http://www.boehringer-ingelheim.com) primary antibody at 1:50 that is cross-reactive with FU.

### Image acquisition.

3-D optical series (z-series) were collected using a Zeiss Axioplan 2 digital imaging microscope (Carl Zeiss, http://www.zeiss.com) equipped with a 100 × (1.4 numerical aperture) plan-apochromat lens and a Coolsnap HQ cooled charge-coupled device camera (Roper Scientific, http://www.roperscientific.com). Z-series extending above and below individual nuclei were collected at 200-nm intervals with a motorized z-motor. Metamorph version 4.5r9 (Universal Imaging, http://www.moleculardevices.com) was employed for computer-based acquisition of 16-bit images comprising of three (DAPI, Alexa-488, and Cy3) individual channels per image. Composite montages of collected images were assembled in Adobe Photoshop 7.0 (Adobe, http://www.adobe.com).

### Image processing and deconvolution.

Individual channels from collected Z-series were imported into SoftWoRx (Applied Precision, http://www.api.com) and converted into DeltaVision files. Converted files were subjected to maximum likelihood expectation deconvolution processing using a constrained iterative algorithm and theoretical optical transfer files generated in SoftWoRx for DAPI (485 nm), Alexa-488 (535 nm), and Cy3 (610 nm). Resulting deconvolved images were used in subsequent 3-D modeling. Images were then assembled in Imaris (Bitplane, http://www.bitplane.com), and 3-D projection images were generated and saved as 12-bit Tiff files.

### 3-D image reconstruction.

Our procedure for the 3-D reconstruction of the image data essentially involved two steps. Step 1) Generation of isosurfaces. Treating the image stack as a 3-D field of values (R, G, B components), we used and implemented the Marching Cubes algorithm [24] to construct isosurfaces. A number of threshold values (i.e., values of R, G, B components) were used to reproduce the correct number of individual bodies or main characteristics of a given image stack or experimental dataset. This procedure was carried out for each dataset manually. No statistical evaluation of the thresholding was carried out other than to compare centroid coordinates at different thresholds, which did not change. Step 2) Foci identification via surface tracking. The results from step 1) resulted in the creation of a series of triangles from the 3-D volume data. A surface-tracking connectivity program was developed and implemented to isolate and identify individual bodies within the volume data. This program takes a triangle as a starting “seed point” and marks all other triangles that touch it within neighboring voxels (using a small distance threshold). The resulting group of triangles then defines an individual “body.” This procedure is repeated until all triangles have been grouped into individual bodies. Geometric parameters (e.g., centroid points) for individual bodies are computed from the coordinates of the constituting group of triangles.

### NN assignment and distances.

See Table 1 for details of the mathematical terminology. Interpoint distances between centroids were computed as Euclidean distances. NN distances are on a standardized scale as measured relative to the maximal possible inert-object distance, taking into account variations in nuclei size. The algorithm for computing median NN distances from inter-centroids is as follows. For all objects in each image in a subgroup: 1) compute the collection of inter-centroid distances (ICDs) for all objects; 2) for each object *i* compute the smallest ICD Mi (NN distance); and 3) compute the median Mi across the collection of objects.

The label of the NN to each CBP centroid focus was then obtained. In situations where ties were observed for the NN, the tie was broken randomly. NN labels were aggregated across all cell images, with every CBP observed retaining a label identifying the body type of its NN. For each NCBP type, the required probability was estimated as a constant term in a logistic regression generalized linear model [28], where the binomial response datum for each cell image is the number of NCBPs that had a CBP as its NN. An offset term was included in the generalized linear model for each cell image that accounted for the total number of CBPs and NCBPs observed in the image. Analytic confidence intervals for the log–odds ratio association parameters were verified using bootstrap resampling. Excess log–odds values were computed and compared against the same quantities computed under random relabeling of objects within an image. All computations were conducted in the R and SPLUS statistics systems; R code is available from DAS.

### Distance-based assessment of CBP spatial organization.

In our previous studies [5,38], we described the assessment of PML nuclear body spatial organization in relation to specific genomic loci via statistical hypothesis tests (specifically, using parametric *t* tests). Here we use nonparametric alternatives to these tests, and Monte Carlo exact methods (see [26]) to assess statistical significance. The extracted 3-D coordinates for CBP and NCBP foci were used to provide empirical distribution functions (EDFs) of NN distances from each NCBP focus to the nearest CBP location. Differences between the distributions for different foci were then tested using a two-sample Kolmogorov-Smirnov statistic (e.g., see [25]), utilizing randomization procedures (calibration against randomly relabeled datasets) where necessary, under the assumption that the distribution of NCBP–CBP distances is not different for different foci. Computation of *p-*values were performed using standard asymptotic reasoning, verified using randomization procedures. See the extended discussion under “Comparison of Different CBP–NCBP Spatial Distributions” in the Results section. Detailed formulae can be found at http://stats.ma.ic.ac.uk/das01/public_html/BioSPP.

## Supporting Information

Figure S1 EDF Plots of NN Distances for NCBP Foci to CBP Kolmogorov-Smirnov statistics were used to compare EDFs for CBP with NCBP NN distances, with computation of *p-*values performed using standard asymptotic reasoning and verified using randomization procedures. Each pairwise plot compares the named components in terms of their NN distances to CBP foci. The labels for each NCBP component is as follows: WG16, RNA PolII (hypophosphorylated); ARNA3, PolII (hyperphosphorylated); SC35, pre-mRNA splicing speckles; AIM1, Aurora B kinase; CREB, Creb/Crem transcriptional activator; S9, p53 phosphorylated Ser9; S20, p53 phosphorylated Ser20; K14, acetylated Lys 14 histone H4; K12, acetylated Lys 12 histone H4; K5, acetylated Lys5 histone H4; FU, fluoro-uridine. Distance have been normalised and are therefore relative distances. The Ks statistic is given in the box and refers to how significant the observed differences are.
(2.8 MB PDF)

Figure S2 Example of Colocalization HeLa cells were irradiated with 10 Gy, fixed with paraformaldehyde, and stained with antibodies recognizing 53BP1 and phosphorylated histone H2AX. 3-D projections of deconvolved image sets are shown.
(88 KB JPG)

## Abbreviations

ARNA3: active RNA PolII
CBP: CREB–binding protein
EDF: empirical distribution function
FU: 5-fluro-uridine
HDAC: histone deacetylase
HAT: histone acetyltransferase
ICD: inter-centroid distance
Lys: lysine
NCBP: non-CBP
NN: nearest-neighbor
PML: promyelocytic leukemia
RNA PolII: RNA polymerase II

## References

[pcbi-0020139-b001] Croft JA, Bridger JM, Boyle S, Perry P, Teague P (1999). Differences in the localization and morphology of chromosomes in the human nucleus. J Cell Biol.

[pcbi-0020139-b002] Tanabe H, Muller S, Neusser M, von Hase J, Calcagno E (2002). Evolutionary conservation of chromosome territory arrangements in cell nuclei from higher primates. Proc Natl Acad Sci U S A.

[pcbi-0020139-b003] Sadoni N, Langer S, Fauth C, Bernardi G, Cremer T (1999). Nuclear organization of mammalian genomes. Polar chromosome territories build up functionally distinct higher order compartments. J Cell Biol.

[pcbi-0020139-b004] Shopland LS, Johnson CV, Byron M, McNeil J, Lawrence JB (2003). Clustering of multiple specific genes and gene-rich R-bands around SC-35 domains: Evidence for local euchromatic neighborhoods. J Cell Biol.

[pcbi-0020139-b005] Shiels C, Islam SA, Vatcheva R, Sasieni P, Sternberg MJ (2001). PML bodies associate specifically with the MHC gene cluster in interphase nuclei. J Cell Sci.

[pcbi-0020139-b006] Shopland LS, Byron M, Stein JL, Lian JB, Stein GS (2001). Replication-dependent histone gene expression is related to Cajal body (CB) association but does not require sustained CB contact. Mol Biol Cell.

[pcbi-0020139-b007] Smith KP, Lawrence JB (2000). Interactions of U2 gene loci and their nuclear transcripts with Cajal (coiled) bodies: Evidence for PreU2 within Cajal bodies. Mol Biol Cell.

[pcbi-0020139-b008] Verschure PJ, Van Der Kraan I, Enserink JM, Mone MJ, Manders EM (2002). Large-scale chromatin organization and the localization of proteins involved in gene expression in human cells. J Histochem Cytochem.

[pcbi-0020139-b009] Grande MA, van der Kraan I, de Jong L, van Driel R (1997). Nuclear distribution of transcription factors in relation to sites of transcription and RNA polymerase II. J Cell Sci.

[pcbi-0020139-b010] Pendergrast PS, Wang C, Hernandez N, Huang S (2002). FBI-1 can stimulate HIV-1 Tat activity and is targeted to a novel subnuclear domain that includes the Tat-P-TEFb–containing nuclear speckles. Mol Biol Cell.

[pcbi-0020139-b011] van Steensel B, Brink M, van der Meulen K, van Binnendijk EP, Wansink DG (1995). Localization of the glucocorticoid receptor in discrete clusters in the cell nucleus. J Cell Sci.

[pcbi-0020139-b012] Hendzel MJ, Kruhlak MJ, Bazett-Jones DP (1998). Organization of highly acetylated chromatin around sites of heterogeneous nuclear RNA accumulation. Mol Biol Cell.

[pcbi-0020139-b013] Mattern KA, van der Kraan I, Schul W, de Jong L, van Driel R (1999). Spatial organization of four hnRNP proteins in relation to sites of transcription, to nuclear speckles, and to each other in interphase nuclei and nuclear matrices of HeLa cells. Exp Cell Res.

[pcbi-0020139-b014] Hendzel MJ, Kruhlak MJ, MacLean NA, Boisvert F, Lever MA (2001). Compartmentalization of regulatory proteins in the cell nucleus. J Steroid Biochem Mol Biol.

[pcbi-0020139-b015] Fischle W, Dequiedt F, Hendzel MJ, Guenther MG, Lazar MA (2002). Enzymatic activity associated with class II HDACs is dependent on a multiprotein complex containing HDAC3 and SMRT/N-CoR. Mol Cell.

[pcbi-0020139-b016] He S, Davie JR (2006). Sp1 and Sp3 foci distribution throughout mitosis. J Cell Sci.

[pcbi-0020139-b017] Schul W, de Jong L, van Driel R (1998). Nuclear neighbours: The spatial and functional organization of genes and nuclear domains. J Cell Biochem.

[pcbi-0020139-b018] McManus KJ, Hendzel MJ (2003). Quantitative analysis of CBP- and P300-induced histone acetylations in vivo using native chromatin. Mol Cell Biol.

[pcbi-0020139-b019] McManus KJ, Hendzel MJ (2001). CBP, a transcriptional coactivator and acetyltransferase. Biochem Cell Biol.

[pcbi-0020139-b020] Diggle PJ (2003). Statistical analysis of spatial point patterns.

[pcbi-0020139-b021] Noordmans HJ, van der Kraan K, van Driel R, Smeulders AW (1998). Randomness of spatial distributions of two proteins in the cell nucleus involved in mRNA synthesis and their relationship. Cytometry.

[pcbi-0020139-b022] Schneider R, Bannister AJ, Myers FA, Thorne AW, Crane-Robinson C (2004). Histone H3 lysine 4 methylation patterns in higher eukaryotic genes. Nat Cell Biol.

[pcbi-0020139-b023] Santos-Rosa H, Schneider R, Bannister AJ, Sherriff J, Bernstein BE (2002). Active genes are tri-methylated at K4 of histone H3. Nature.

[pcbi-0020139-b024] Lorensen WE, Cline HE (1987). Marching cubes: A high resolution 3D surface reconstruction algorithm. Comput Graph.

[pcbi-0020139-b025] Conover WJ (1999). Practical nonparametric statistics.

[pcbi-0020139-b026] Manly BFJ (1997). Randomization, bootstrap, and Monte Carlo methods in biology.

[pcbi-0020139-b027] Knowles DW, Ortiz de Solorzano C, Jones A (2000). Analysis of the 3D spatial organization of cells and subcellular structures in tissue. Farkas DL, Leif RC, editors. Proc SPIE.

[pcbi-0020139-b028] McCullagh P, Nelder JA (1989). Generalized linear models. 2nd edition.

[pcbi-0020139-b029] Thorne AW, Kmiciek D, Mitchelson K, Sautiere P, Crane-Robinson C (1990). Patterns of histone acetylation. Eur J Biochem.

[pcbi-0020139-b030] Turner BM, O'Neill LP, Allan IM (1989). Histone H4 acetylation in human cells. Frequency of acetylation at different sites defined by immunolabeling with site-specific antibodies. FEBS Lett.

[pcbi-0020139-b031] Zhang K, Williams KE, Huang L, Yau P, Siino JS (2002). Histone acetylation and deacetylation: Identification of acetylation and methylation sites of HeLa histone H4 by mass spectrometry. Mol Cell Proteomics.

[pcbi-0020139-b032] Zhang DE, Nelson DA (1988). Histone acetylation in chicken erythrocytes. Rates of deacetylation in immature and mature red blood cells. Biochem J.

[pcbi-0020139-b033] Davie JR, Hendzel MJ (1994). Multiple functions of dynamic histone acetylation. J Cell Biochem.

[pcbi-0020139-b034] Hendzel MJ, Sun JM, Chen HY, Rattner JB, Davie JR (1994). Histone acetyltransferase is associated with the nuclear matrix. J Biol Chem.

[pcbi-0020139-b035] Moen PT, Smith KP, Lawrence JB (1995). Compartmentalization of specific pre-mRNA metabolism: An emerging view. Hum Mol Genet.

[pcbi-0020139-b036] Xing Y, Johnson CV, Dobner PR, Lawrence JB (1993). Higher level organization of individual gene transcription and RNA splicing. Science.

[pcbi-0020139-b037] Xing Y, Johnson CV, Moen PT, McNeil JA, Lawrence J (1995). Nonrandom gene organization: Structural arrangements of specific pre-mRNA transcription and splicing with SC-35 domains. J Cell Biol.

[pcbi-0020139-b038] Wang J, Shiels C, Sasieni P, Wu PJ, Islam SA (2004). Promyelocytic leukemia nuclear bodies associate with transcriptionally active genomic regions. J Cell Biol.

[pcbi-0020139-b039] Bolzer A, Kreth G, Solovei I, Koehler D, Saracoglu K (2005). Three-dimensional maps of all chromosomes in human male fibroblast nuclei and prometaphase rosettes. PLoS Biol.

[pcbi-0020139-b040] Boyle S, Gilchrist S, Bridger JM, Mahy NL, Ellis JA (2001). The spatial organization of human chromosomes within the nuclei of normal and emerin-mutant cells. Hum Mol Genet.

[pcbi-0020139-b041] Cremer T, Cremer C (2001). Chromosome territories, nuclear architecture and gene regulation in mammalian cells. Nat Rev Genet.

[pcbi-0020139-b042] Parada LA, Sotiriou S, Misteli T (2004). Spatial genome organization. Exp Cell Res.

[pcbi-0020139-b043] Solovei I, Schermelleh L, During K, Engelhardt A, Stein S (2004). Differences in centromere positioning of cycling and postmitotic human cell types. Chromosoma.

[pcbi-0020139-b044] Fuchsova B, Hozak P (2002). The localization of nuclear DNA helicase II in different nuclear compartments is linked to transcription. Exp Cell Res.

[pcbi-0020139-b045] Nielsen JA, Hudson LD, Armstrong RC (2002). Nuclear organization in differentiating oligodendrocytes. J Cell Sci.

[pcbi-0020139-b046] Grande MA, van der Kraan I, van Steensel B, Schul W, de The H (1996). PML-containing nuclear bodies: Their spatial distribution in relation to other nuclear components. J Cell Biochem.

[pcbi-0020139-b047] van Steensel B, van Binnendijk EP, Hornsby CD, van der Voort HT, Krozowski ZS (1996). Partial colocalization of glucocorticoid and mineralocorticoid receptors in discrete compartments in nuclei of rat hippocampus neurons. J Cell Sci.

[pcbi-0020139-b048] Bleoo S, Sun X, Hendzel MJ, Rowe JM, Packer M (2001). Association of human DEAD box protein DDX1 with a cleavage stimulation factor involved in 3′-end processing of pre-MRNA. Mol Biol Cell.

