# Supplementary material for: The Transcriptional Regulator CBP Has Defined Spatial Associations within Interphase Nuclei
Source: PLoS Comput Biol. 2006 Oct 20;2(10):e139. doi: 10.1371/journal.pcbi.0020139 (PMC1617132; doi:10.1371/journal.pcbi.0020139)
Supplement: Supplementary file 1 [file pcbi.0020139.sg001.pdf]

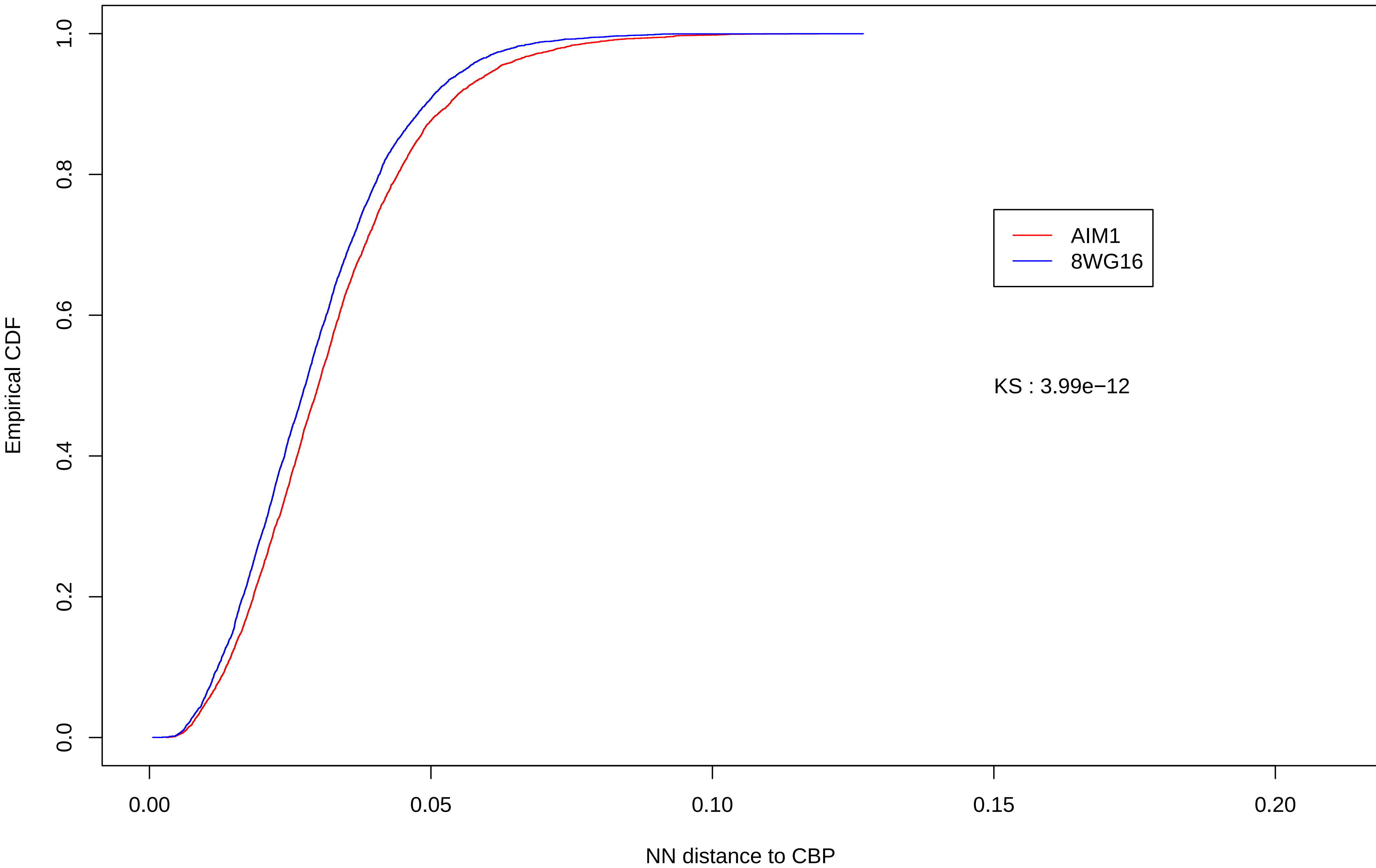

Empirical CDF
0.0
0.2
0.4
0.6
0.8
1.0
0.00
0.05
0.10
0.15
0.20
NN distance to CBP
AIM1
8WG16
KS : 3.99e−12

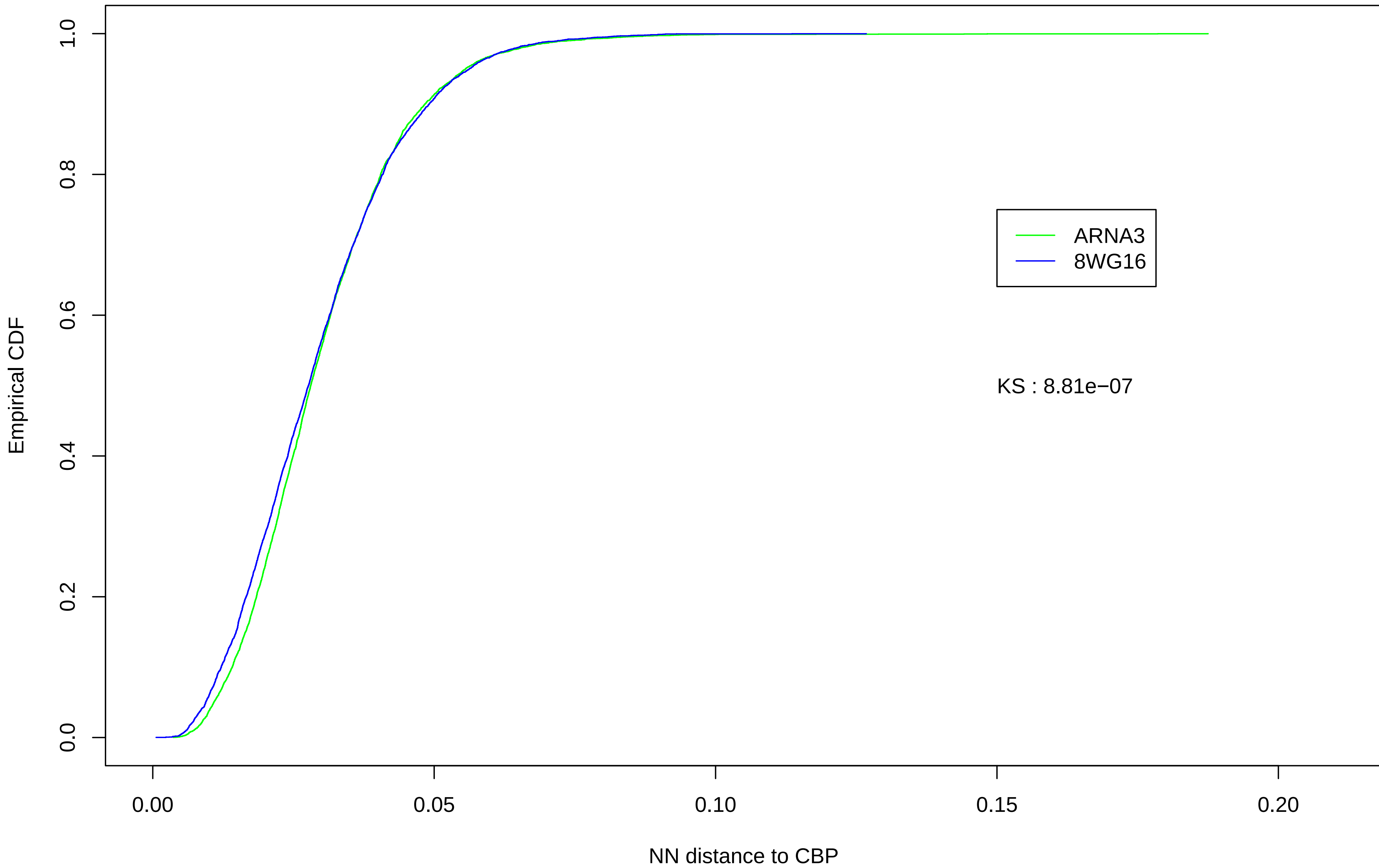

Empirical CDF
0.0
0.2
0.4
0.6
0.8
1.0
0.00
0.05
0.10
0.15
0.20
NN distance to CBP
ARNA3
8WG16
KS : 8.81e−07

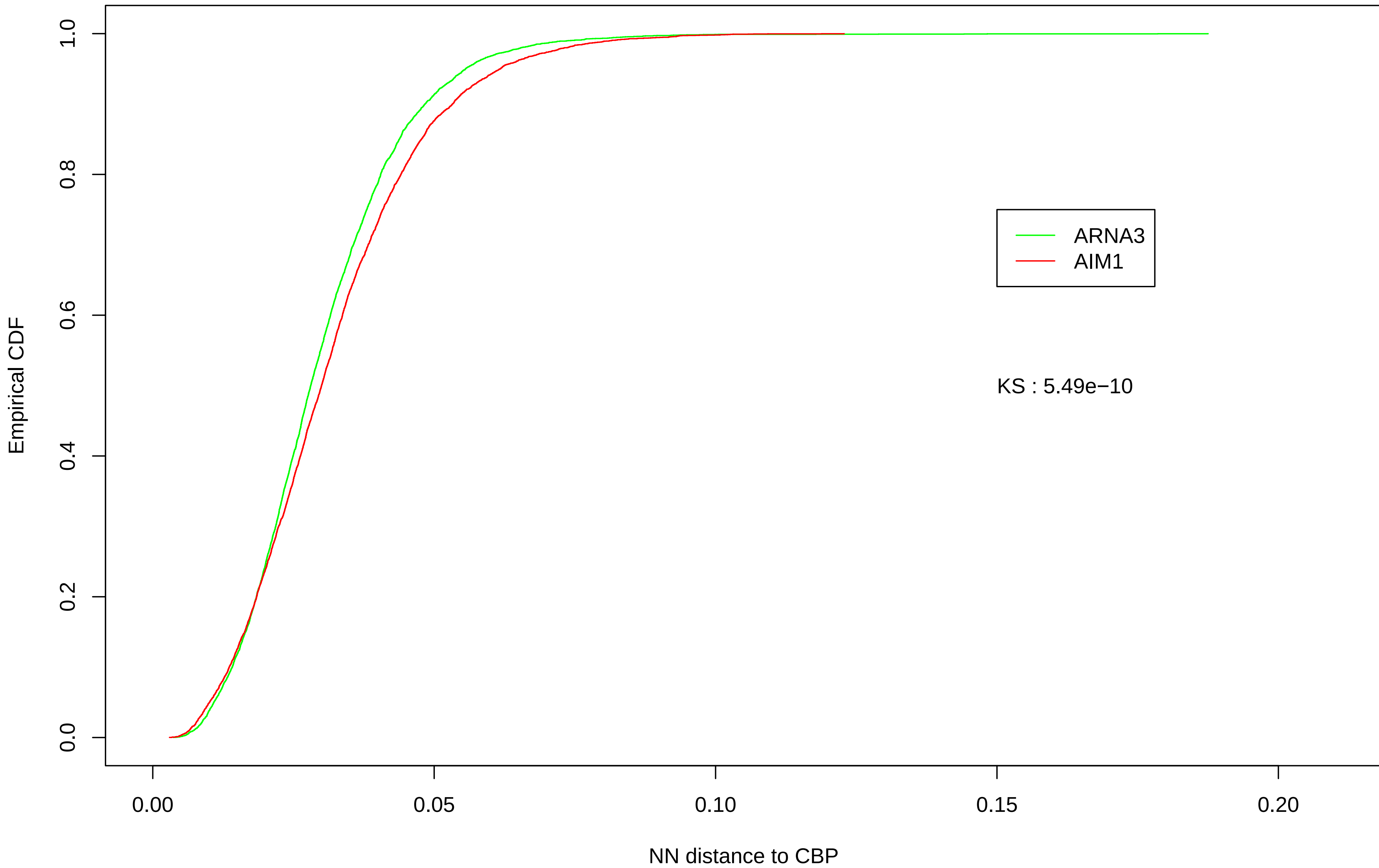

ARNA3
AIM1
KS : 5.49e−10
Empirical CDF
0.0
0.2
0.4
0.6
0.8
1.0
0.00
0.05
0.10
0.15
0.20
NN distance to CBP

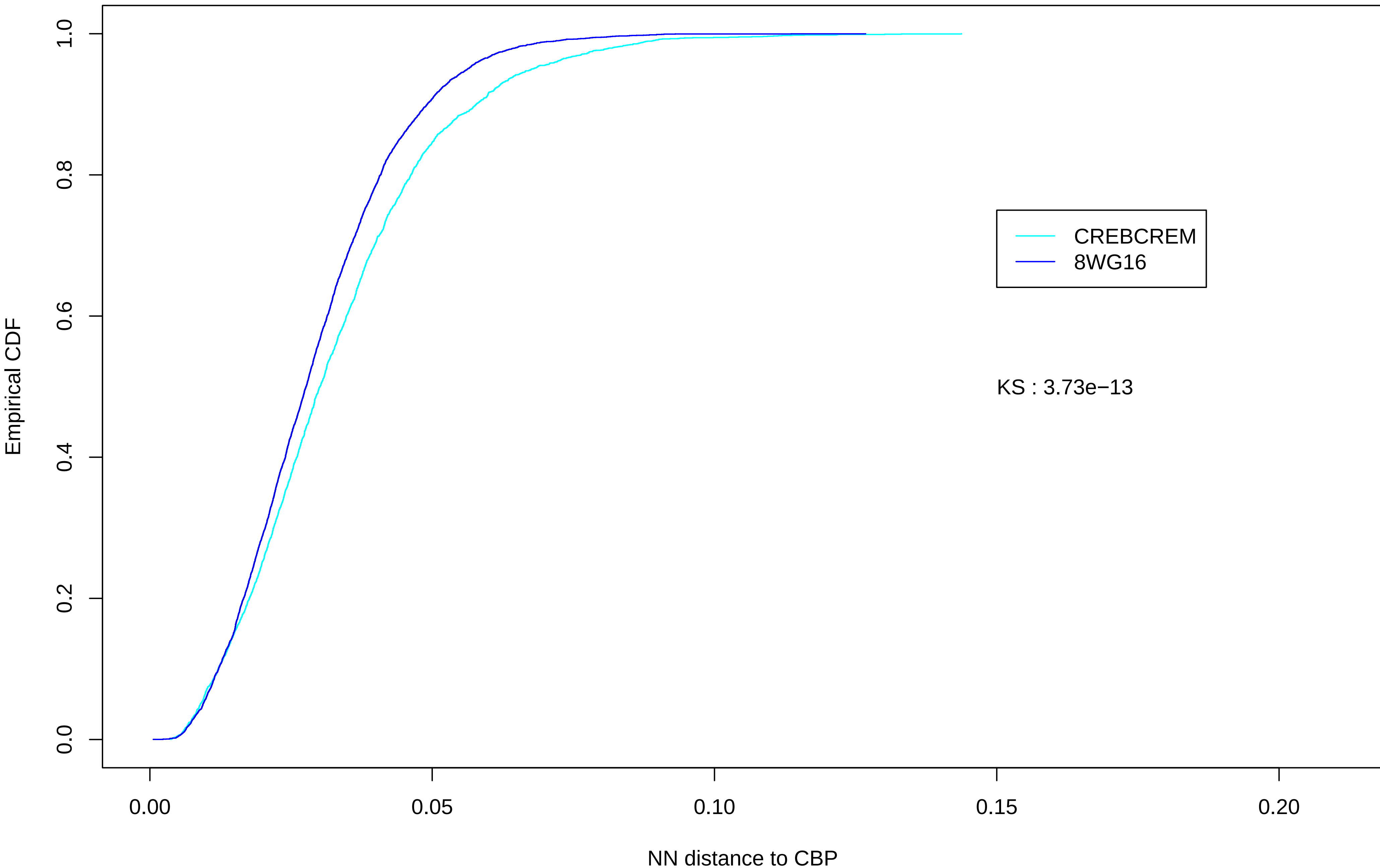

Empirical CDF
NN distance to CBP
CREBCREM
8WG16
KS : 3.73e−13
0.0
0.2
0.4
0.6
0.8
1.0
0.00
0.05
0.10
0.15
0.20

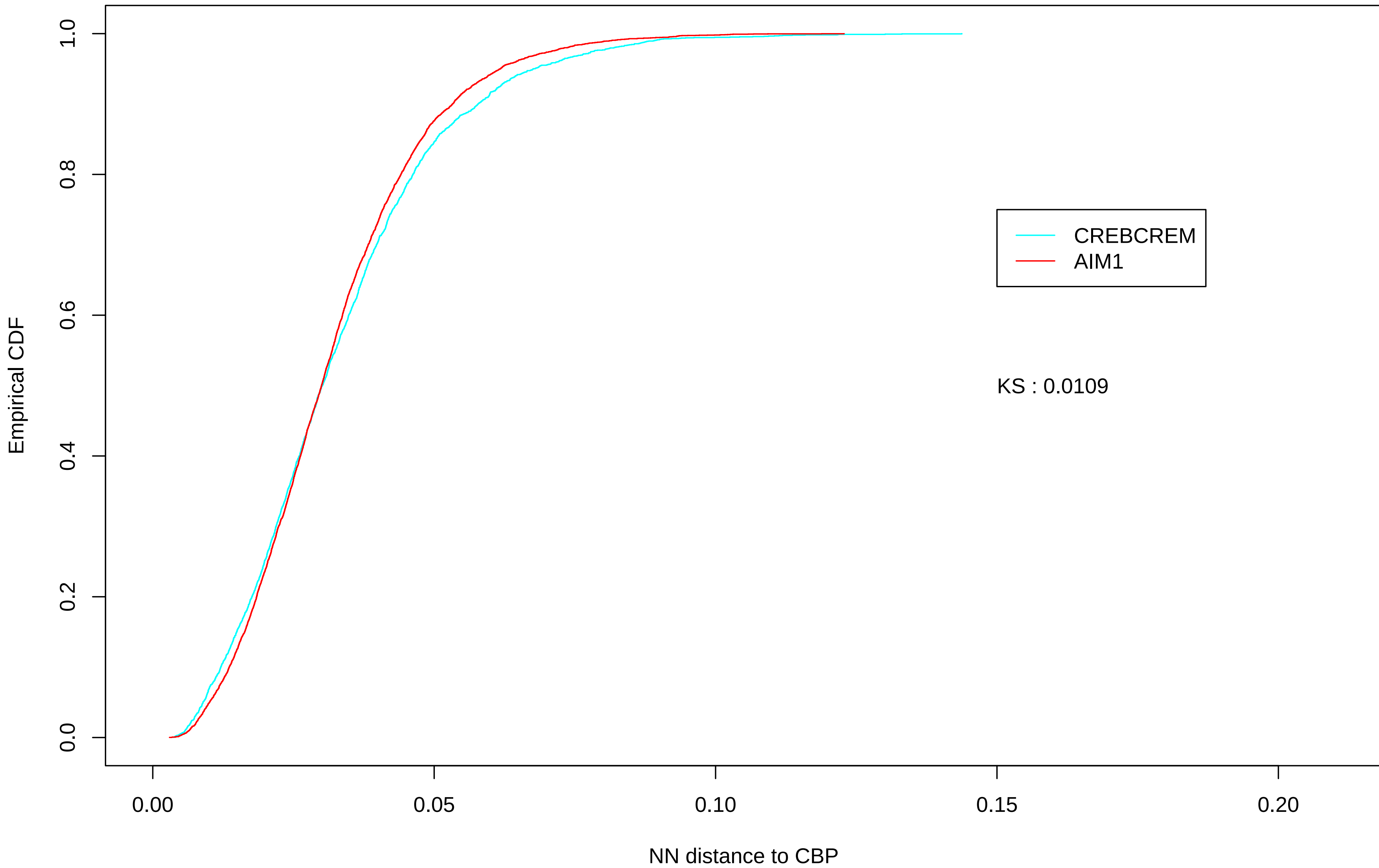
CREBCREM
AIM1
KS : 0.0109
Empirical CDF
NN distance to CBP
0.0
0.2
0.4
0.6
0.8
1.0
0.00
0.05
0.10
0.15
0.20

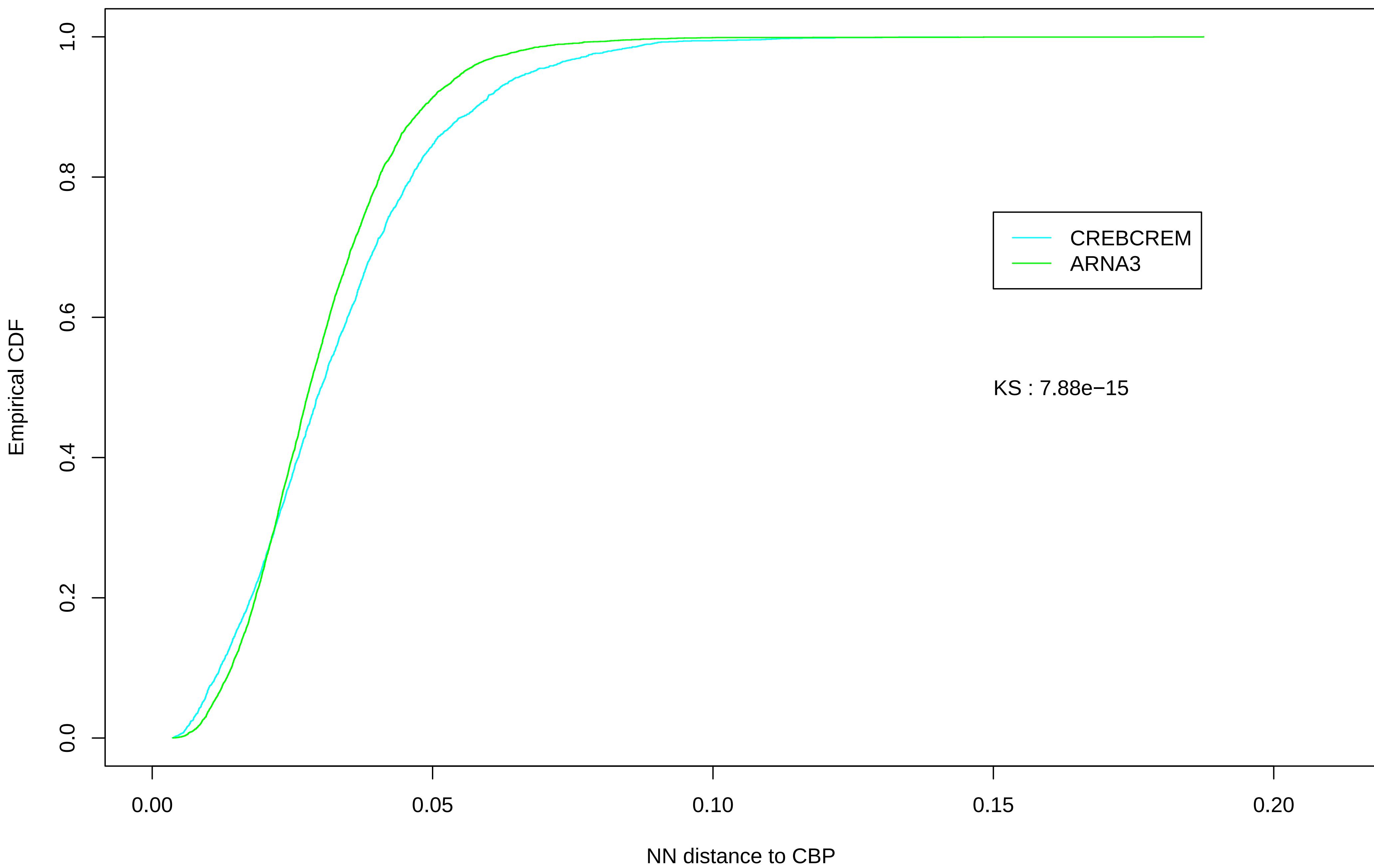

Empirical CDF
0.0
0.2
0.4
0.6
0.8
1.0
0.00
0.05
0.10
0.15
0.20
NN distance to CBP
CREBCREM
ARNA3
KS : 7.88e−15

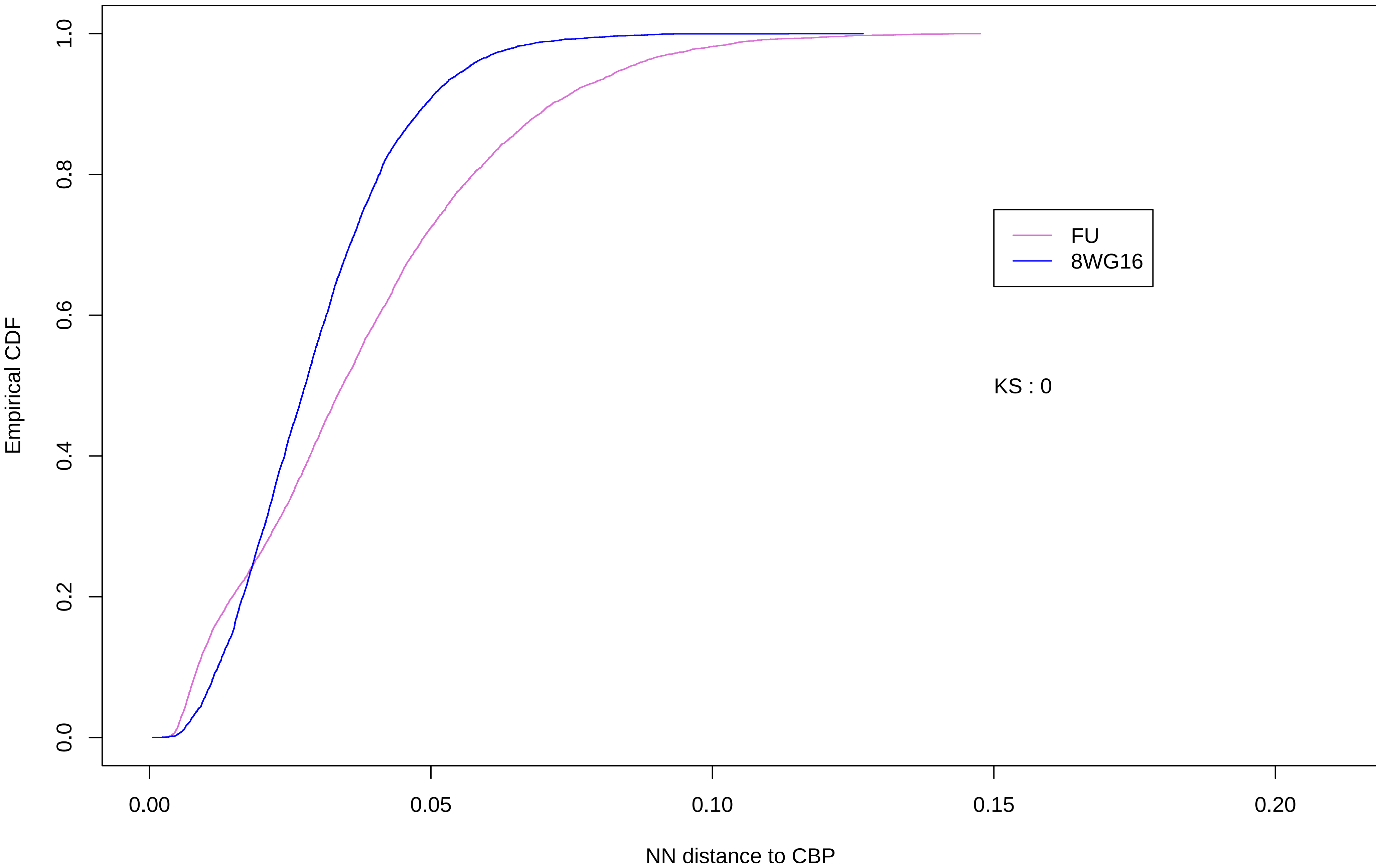

Empirical CDF
0.0
0.2
0.4
0.6
0.8
1.0
0.00
0.05
0.10
0.15
0.20
NN distance to CBP
FU
8WG16
KS : 0

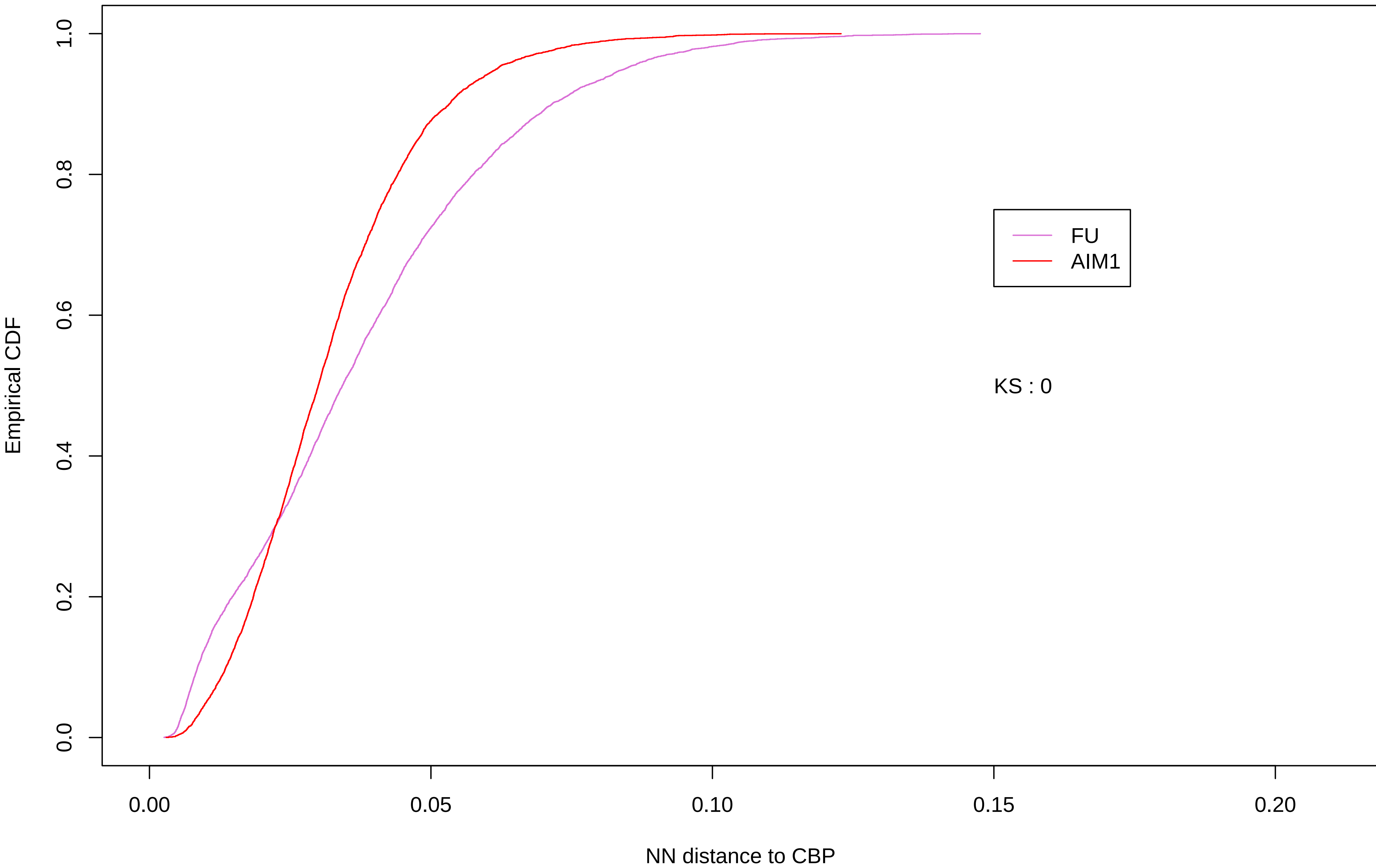
Empirical CDF
0.0
0.2
0.4
0.6
0.8
1.0
0.00
0.05
0.10
0.15
0.20
NN distance to CBP
FU
AIM1
KS : 0

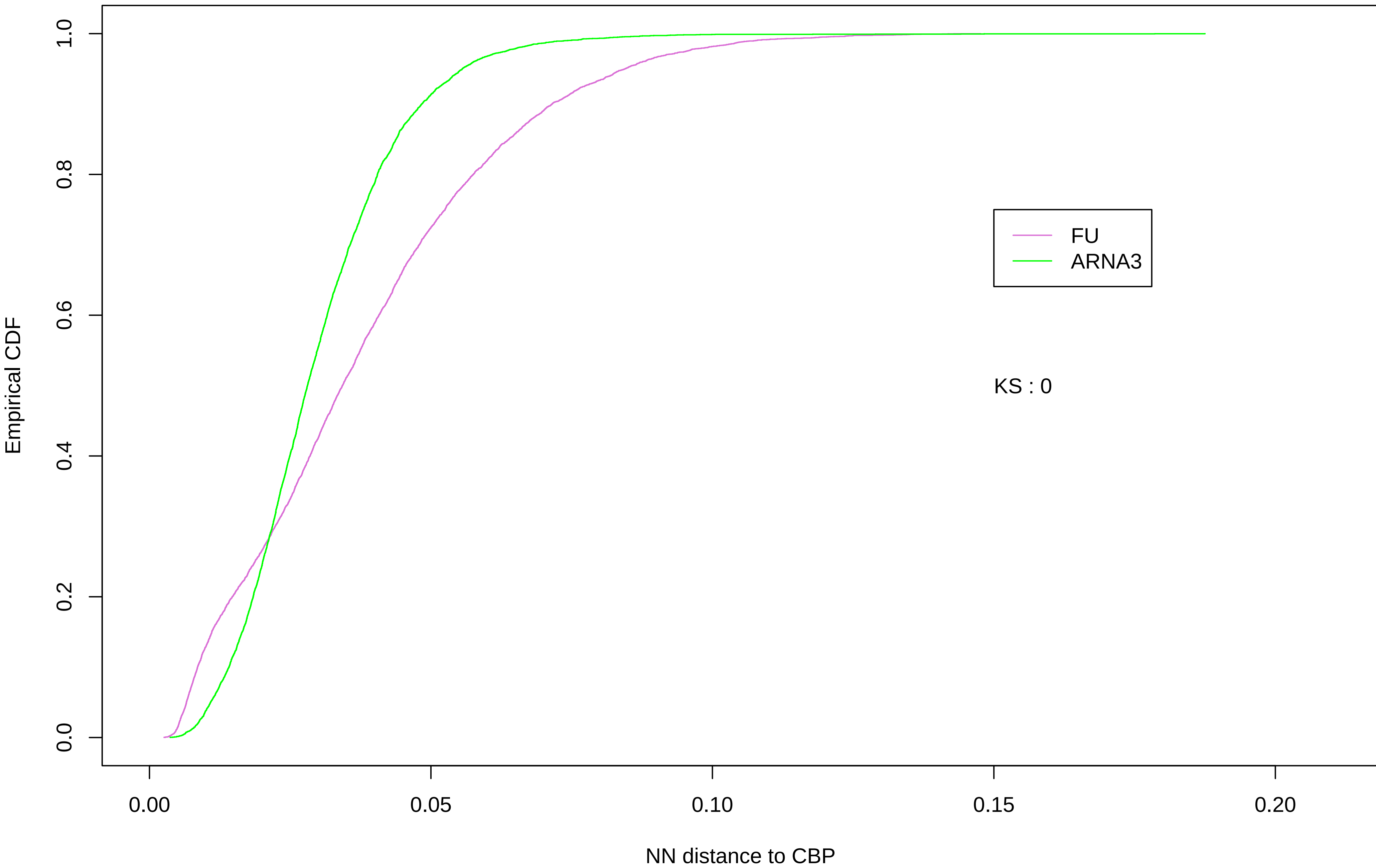
Empirical CDF
NN distance to CBP
0.0
0.2
0.4
0.6
0.8
1.0
0.00
0.05
0.10
0.15
0.20
FU
ARNA3
KS : 0

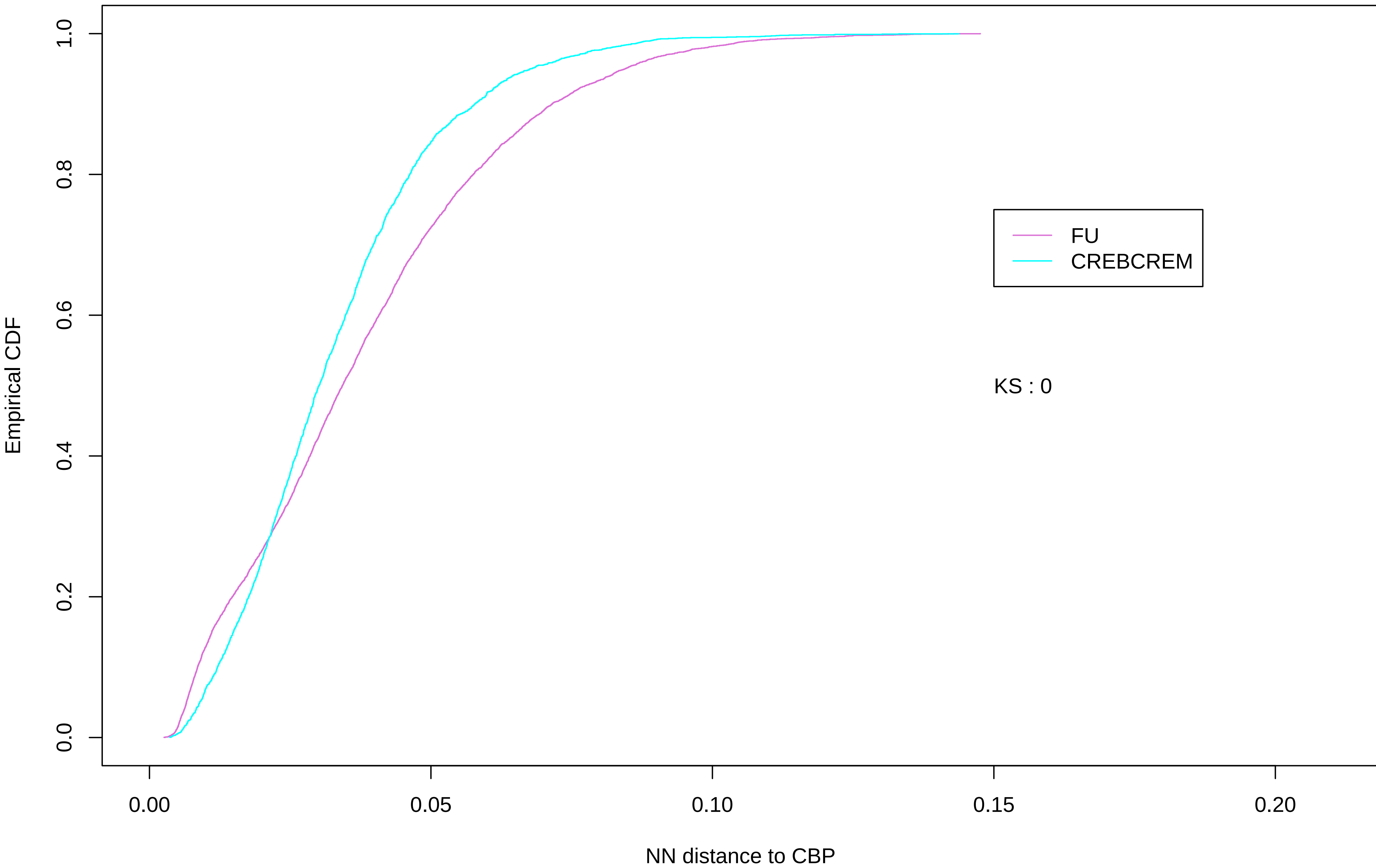
FU
CREBCREM
KS : 0
Empirical CDF
1.0
0.8
0.6
0.4
0.2
0.0
0.00
0.05
0.10
0.15
0.20
NN distance to CBP

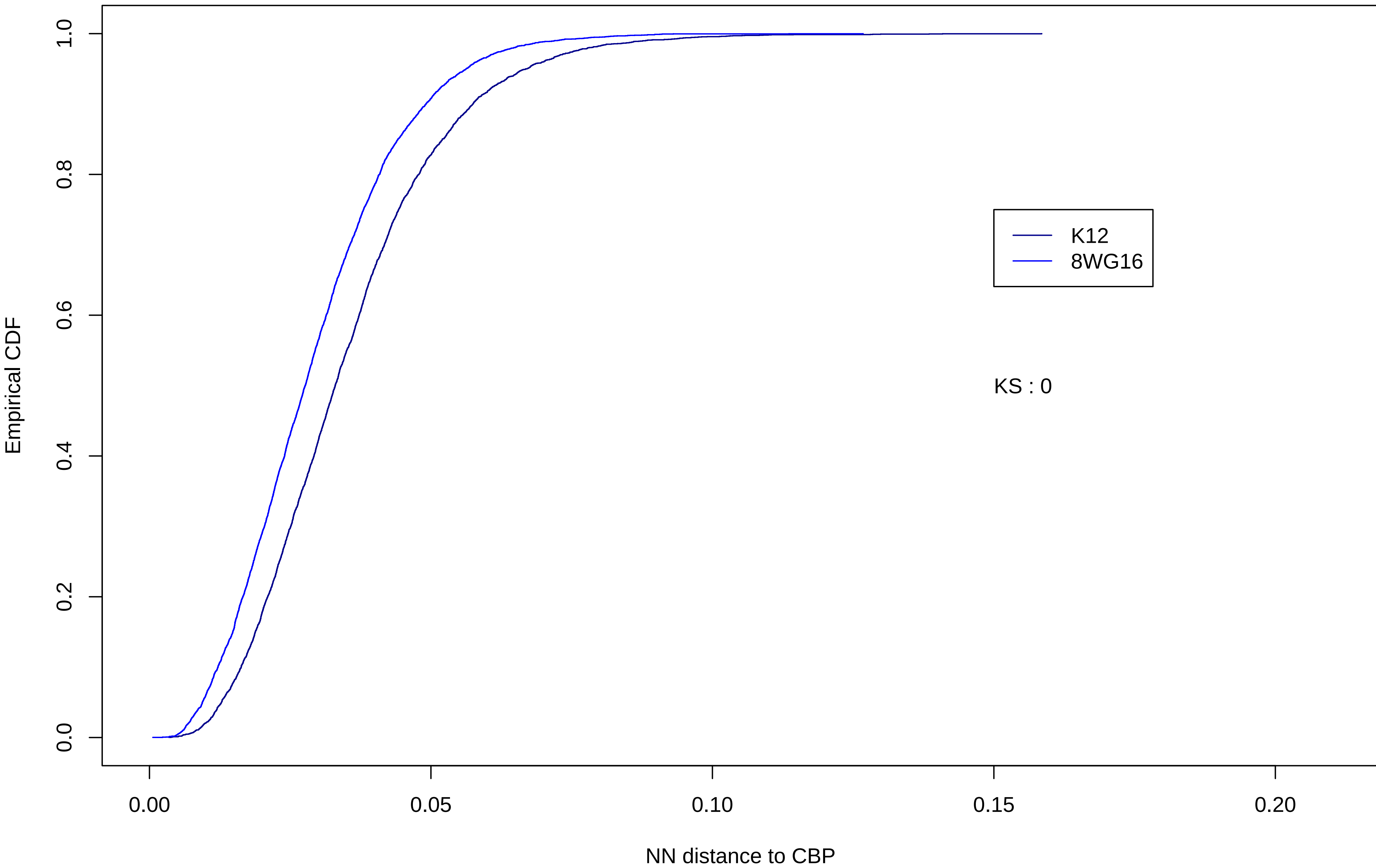

Empirical CDF
0.0
0.2
0.4
0.6
0.8
1.0
0.00
0.05
0.10
0.15
0.20
NN distance to CBP
K12
8WG16
KS : 0

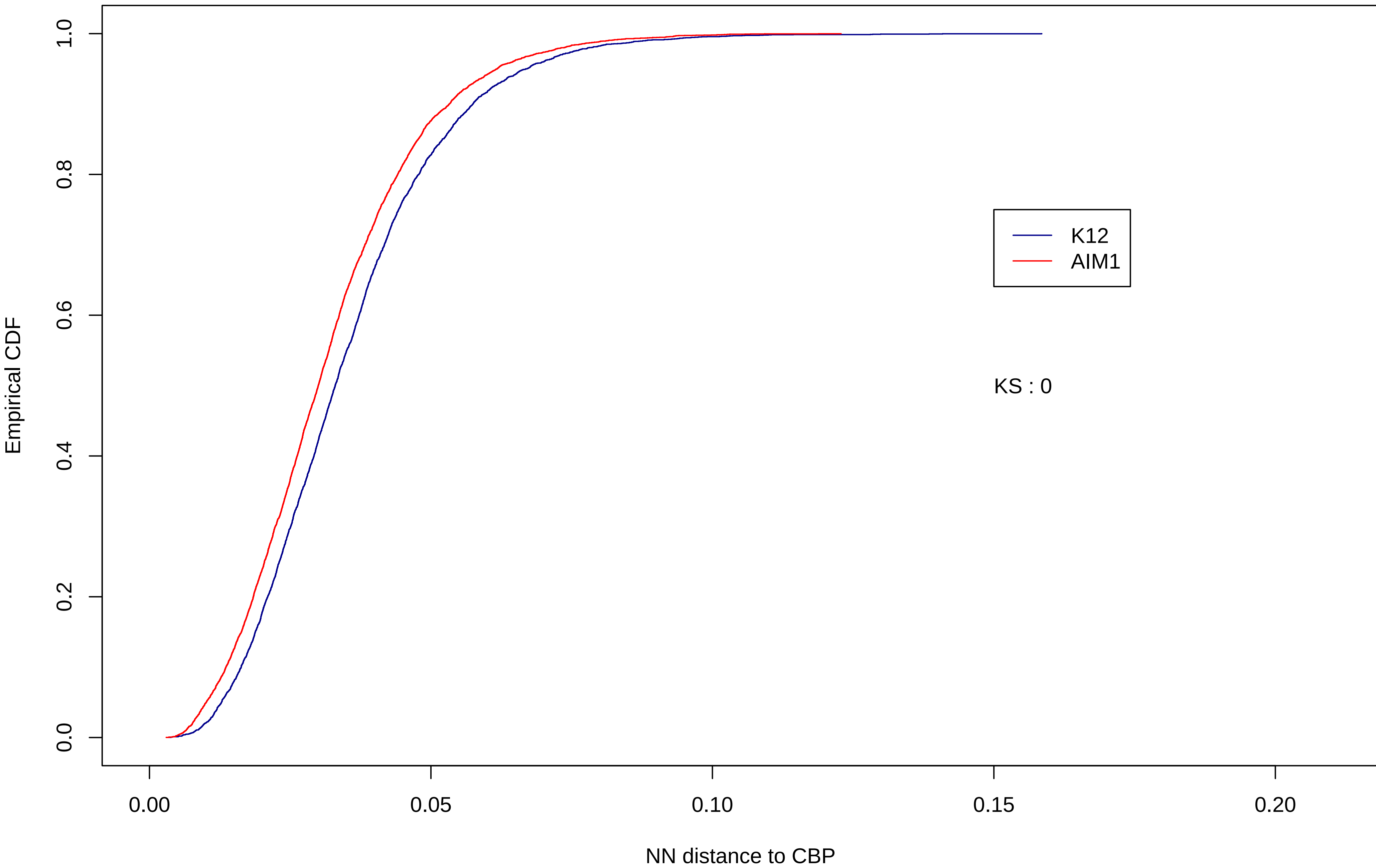
K12
AIM1
KS : 0
Empirical CDF
NN distance to CBP
0.0
0.2
0.4
0.6
0.8
1.0
0.00
0.05
0.10
0.15
0.20

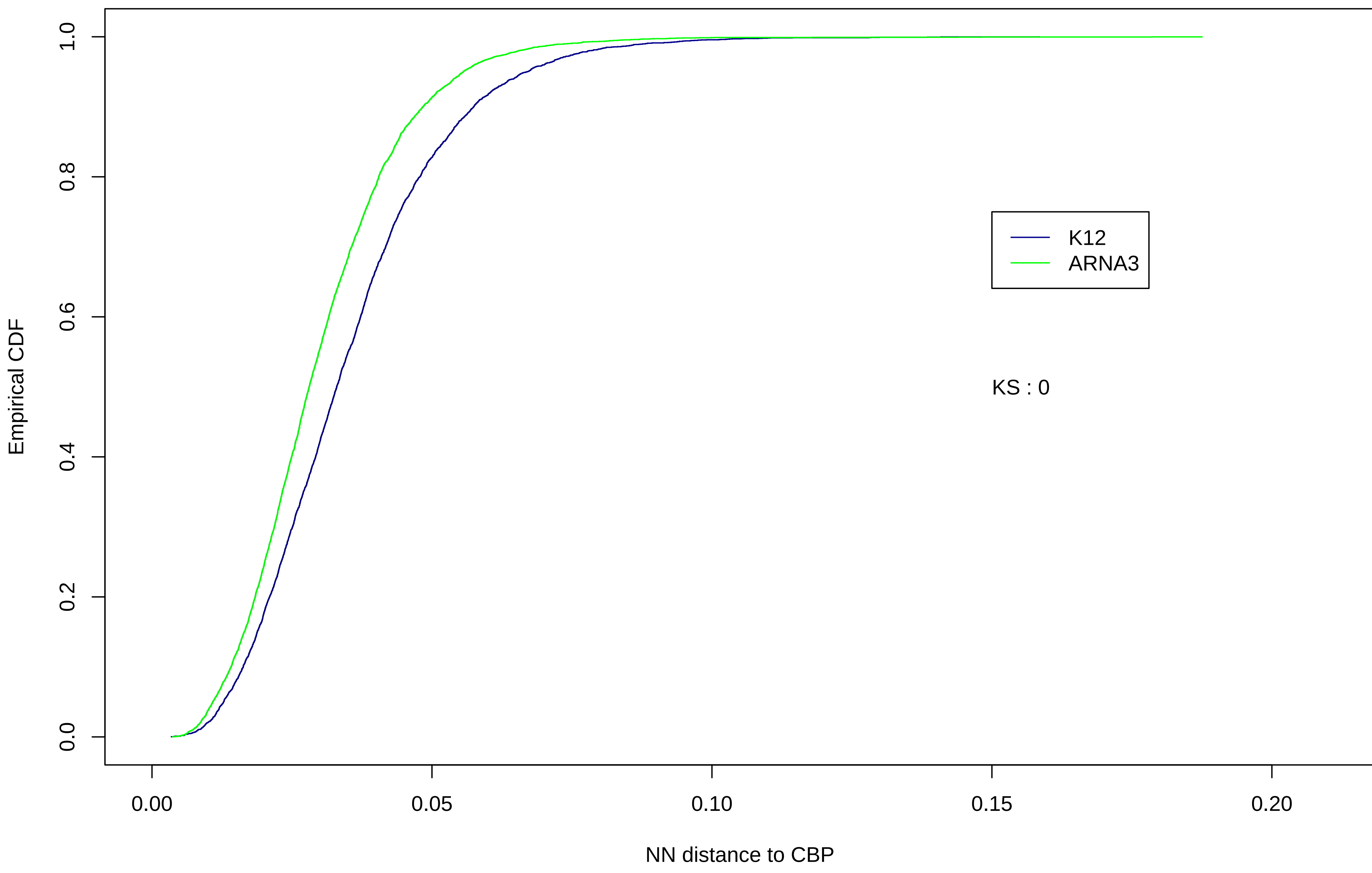

Empirical CDF
NN distance to CBP
0.0
0.2
0.4
0.6
0.8
1.0
0.00
0.05
0.10
0.15
0.20
K12
ARNA3
KS : 0

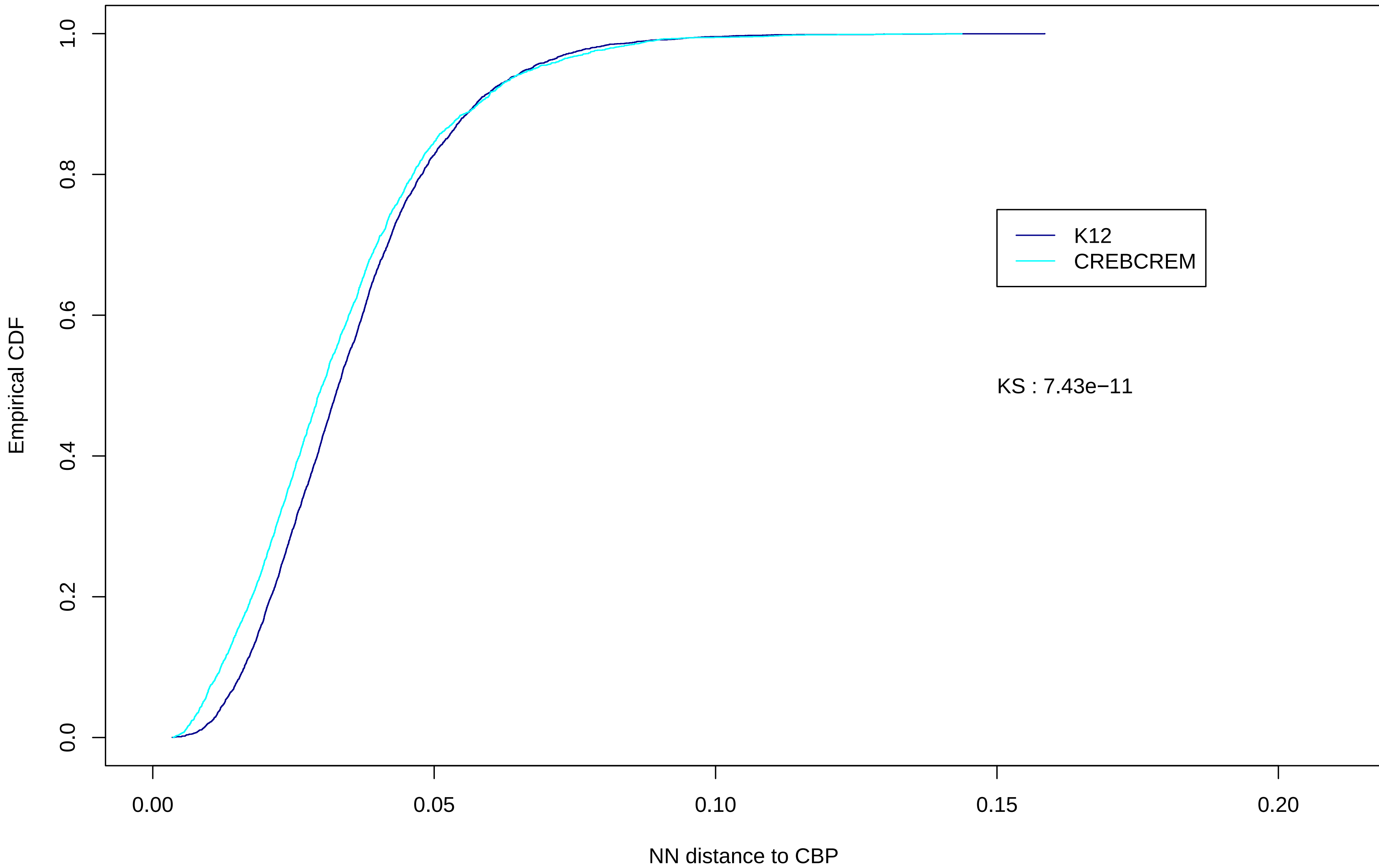

K12
CREBCREM
KS : 7.43e−11
Empirical CDF
NN distance to CBP
0.0
0.2
0.4
0.6
0.8
1.0
0.00
0.05
0.10
0.15
0.20

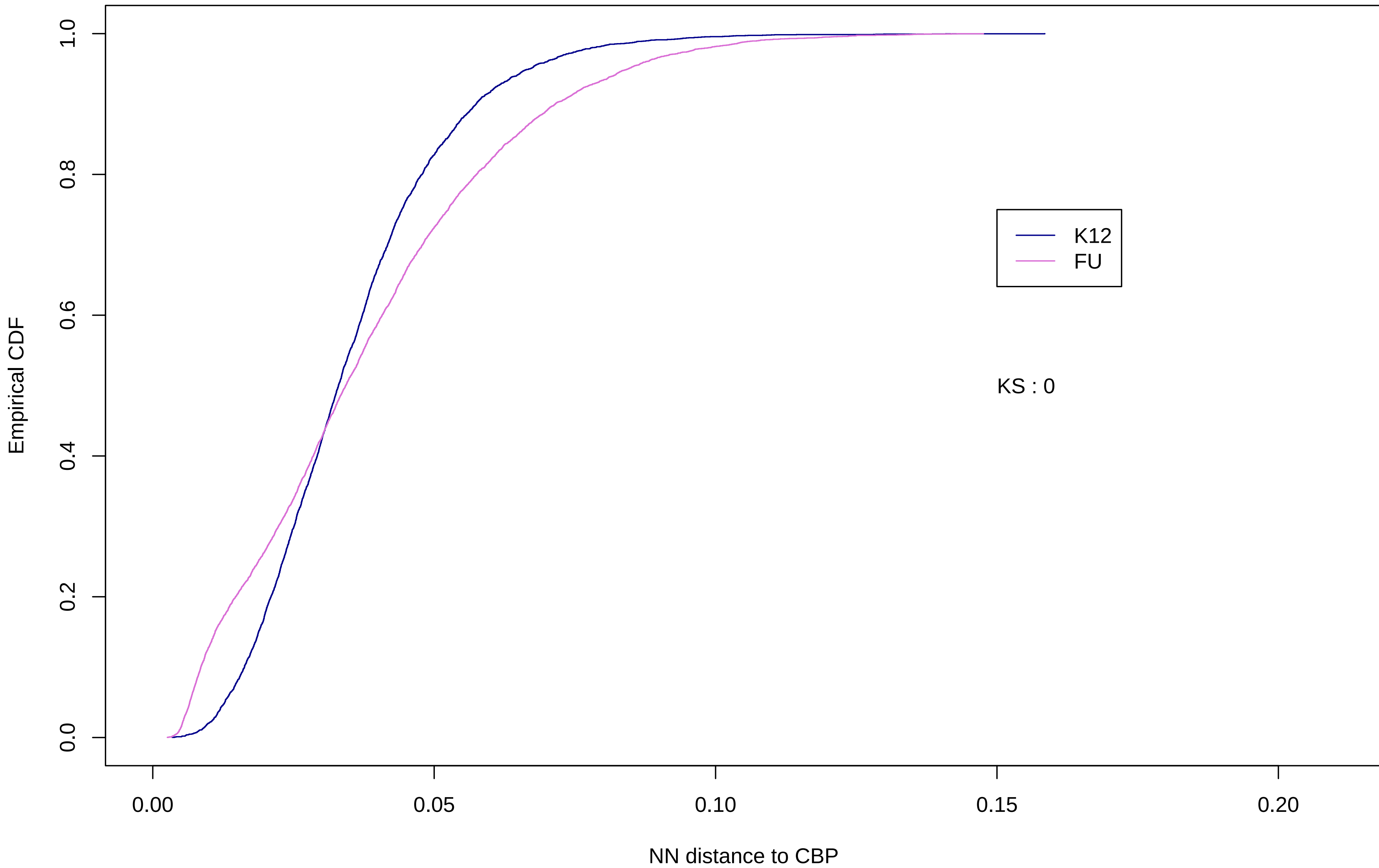

Empirical CDF
NN distance to CBP
0.0
0.2
0.4
0.6
0.8
1.0
0.00
0.05
0.10
0.15
0.20
K12
FU
KS : 0

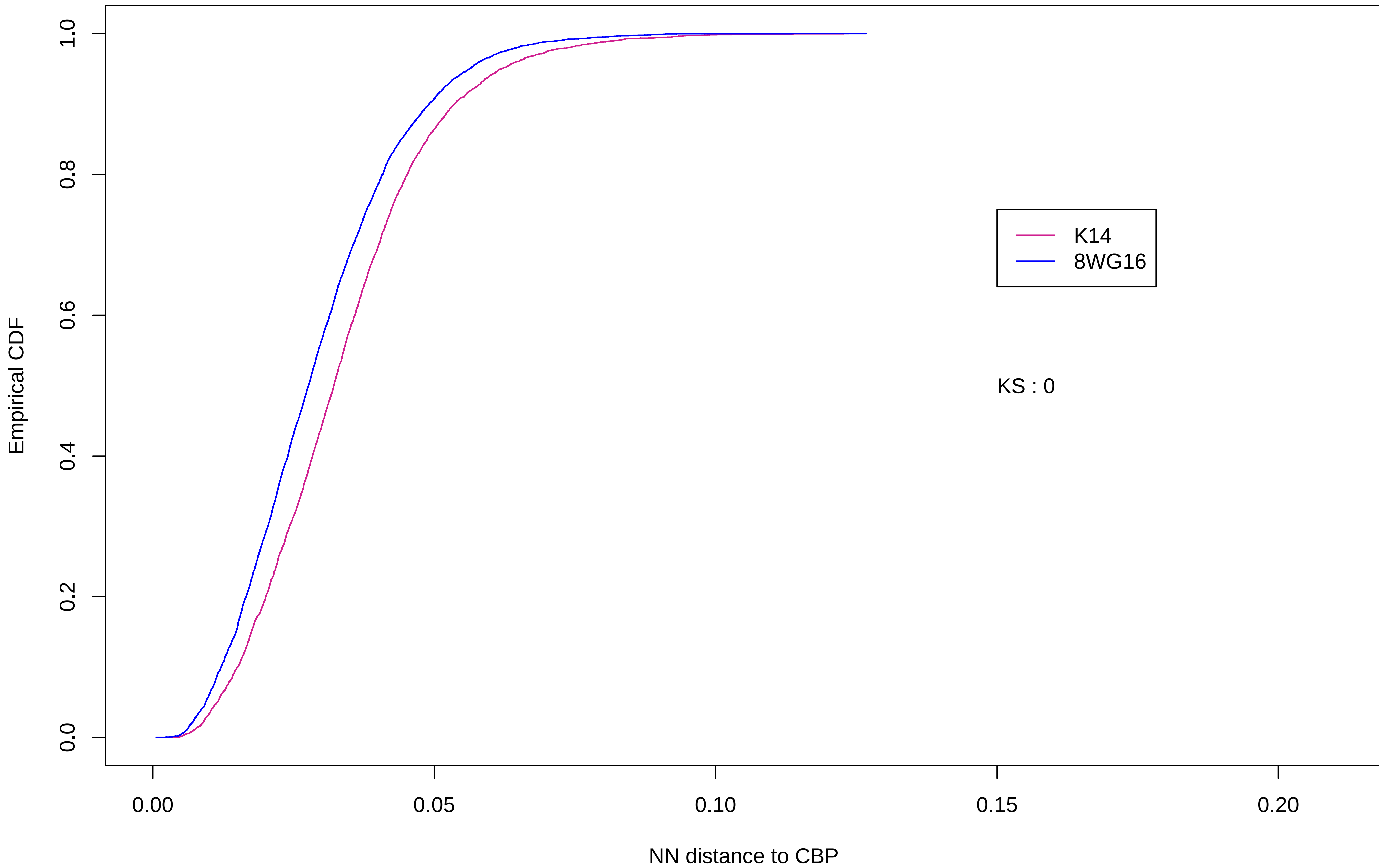

Empirical CDF
0.0
0.2
0.4
0.6
0.8
1.0
0.00
0.05
0.10
0.15
0.20
NN distance to CBP
K14
8WG16
KS : 0

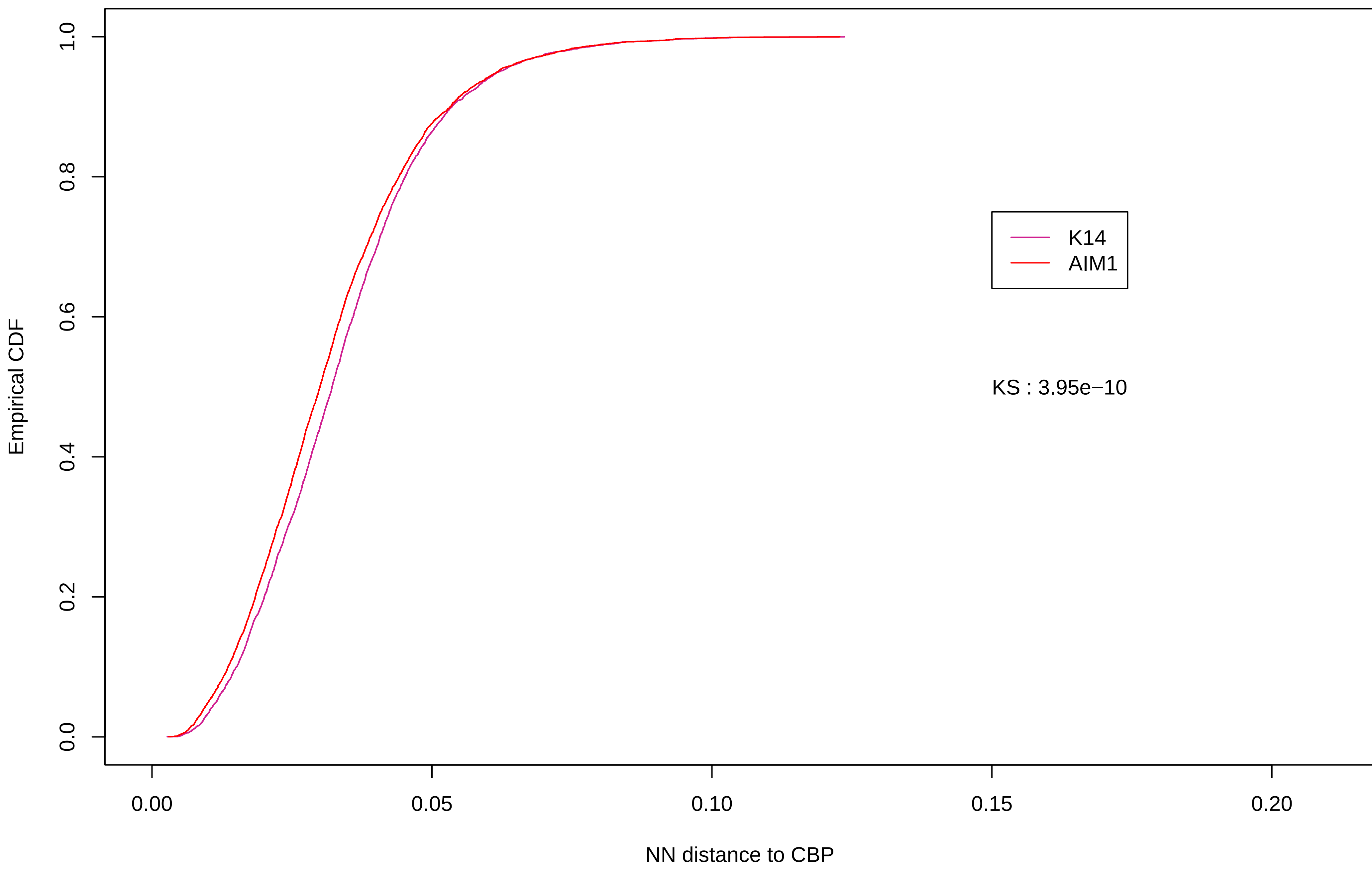

K14
AIM1
KS : 3.95e−10
Empirical CDF
0.0
0.2
0.4
0.6
0.8
1.0
0.00
0.05
0.10
0.15
0.20
NN distance to CBP

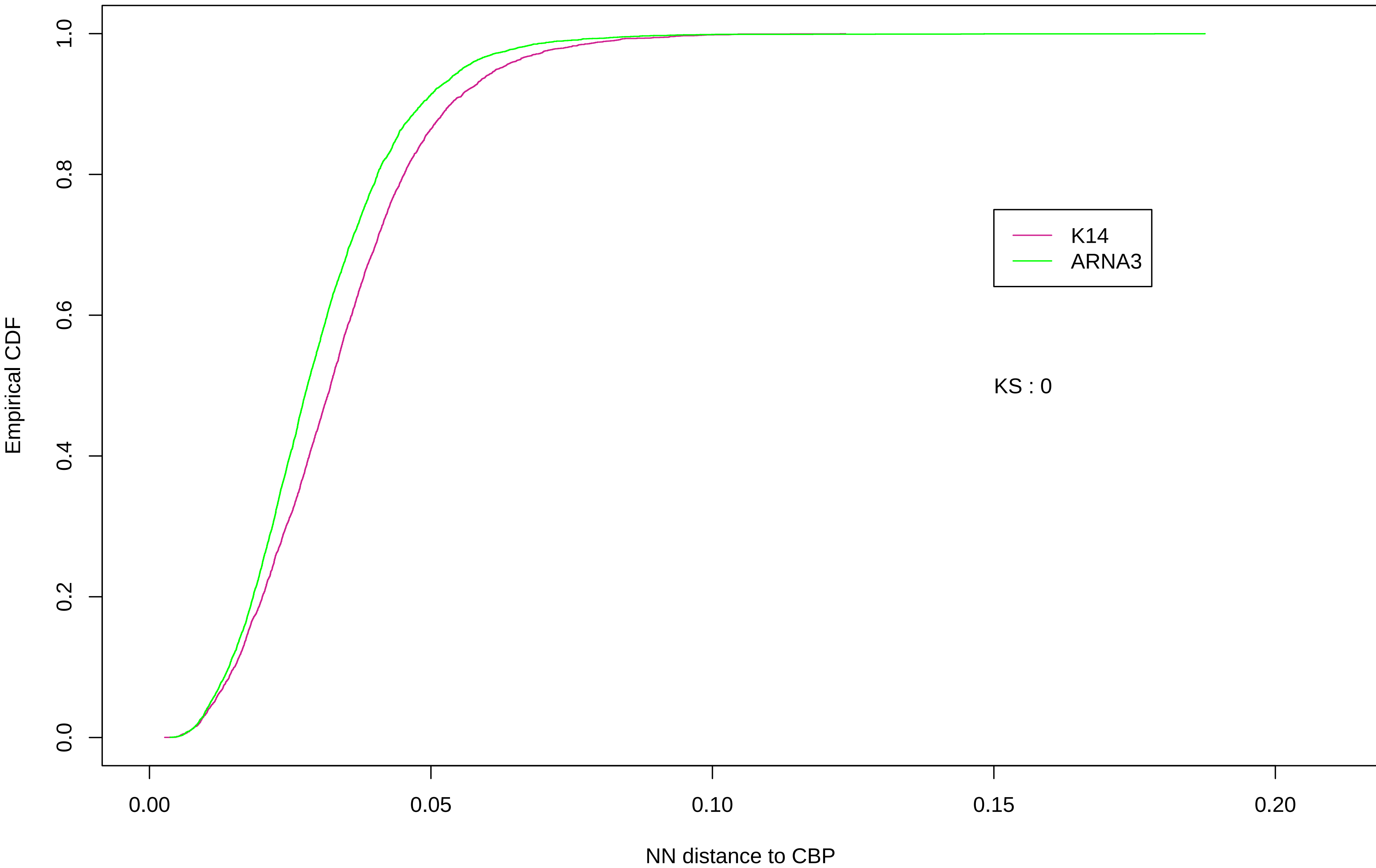

Empirical CDF
NN distance to CBP
0.0
0.2
0.4
0.6
0.8
1.0
0.00
0.05
0.10
0.15
0.20
K14
ARNA3
KS : 0

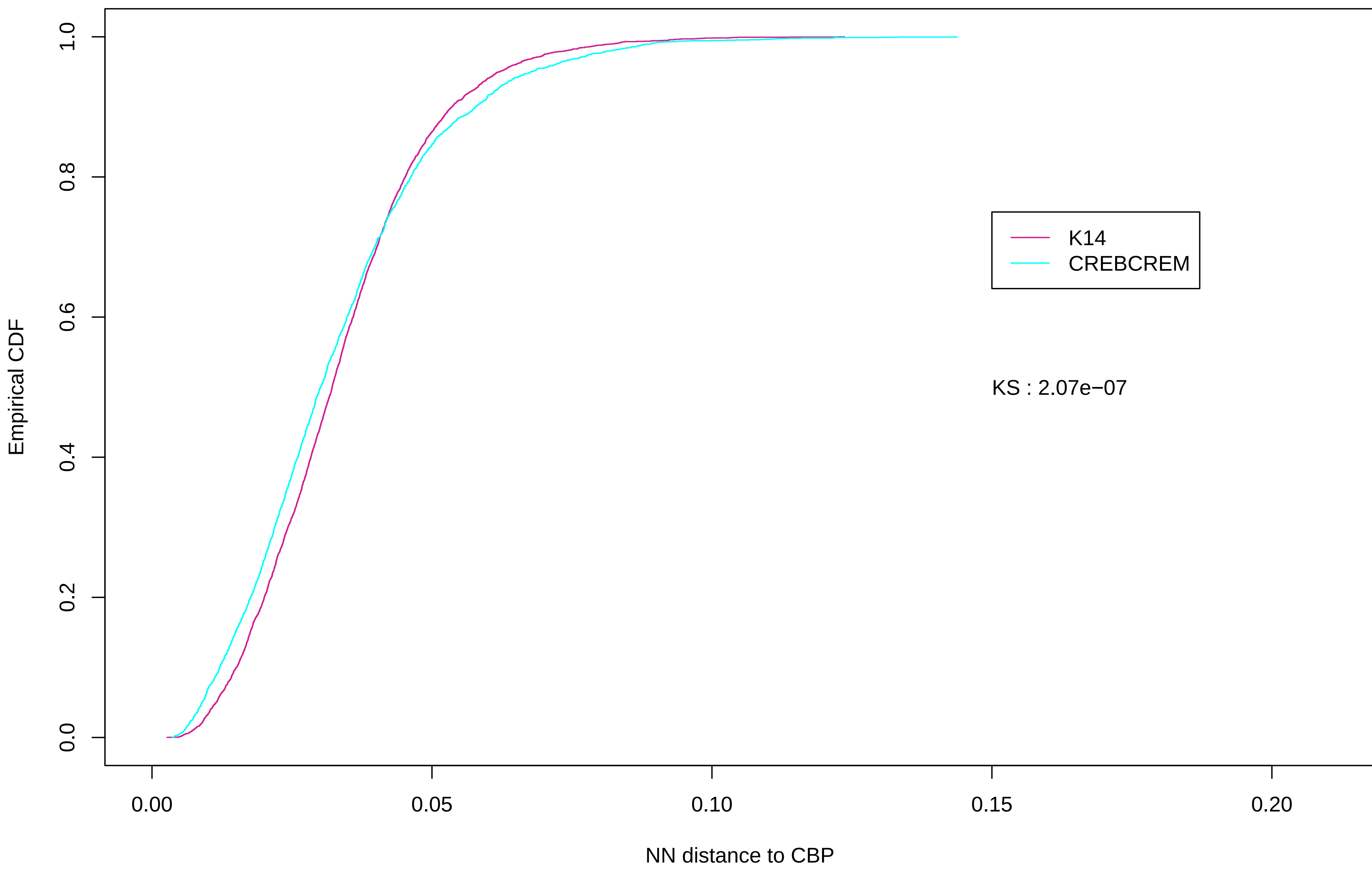

K14
CREBCREM
KS : 2.07e−07
Empirical CDF
NN distance to CBP
0.0
0.2
0.4
0.6
0.8
1.0
0.00
0.05
0.10
0.15
0.20

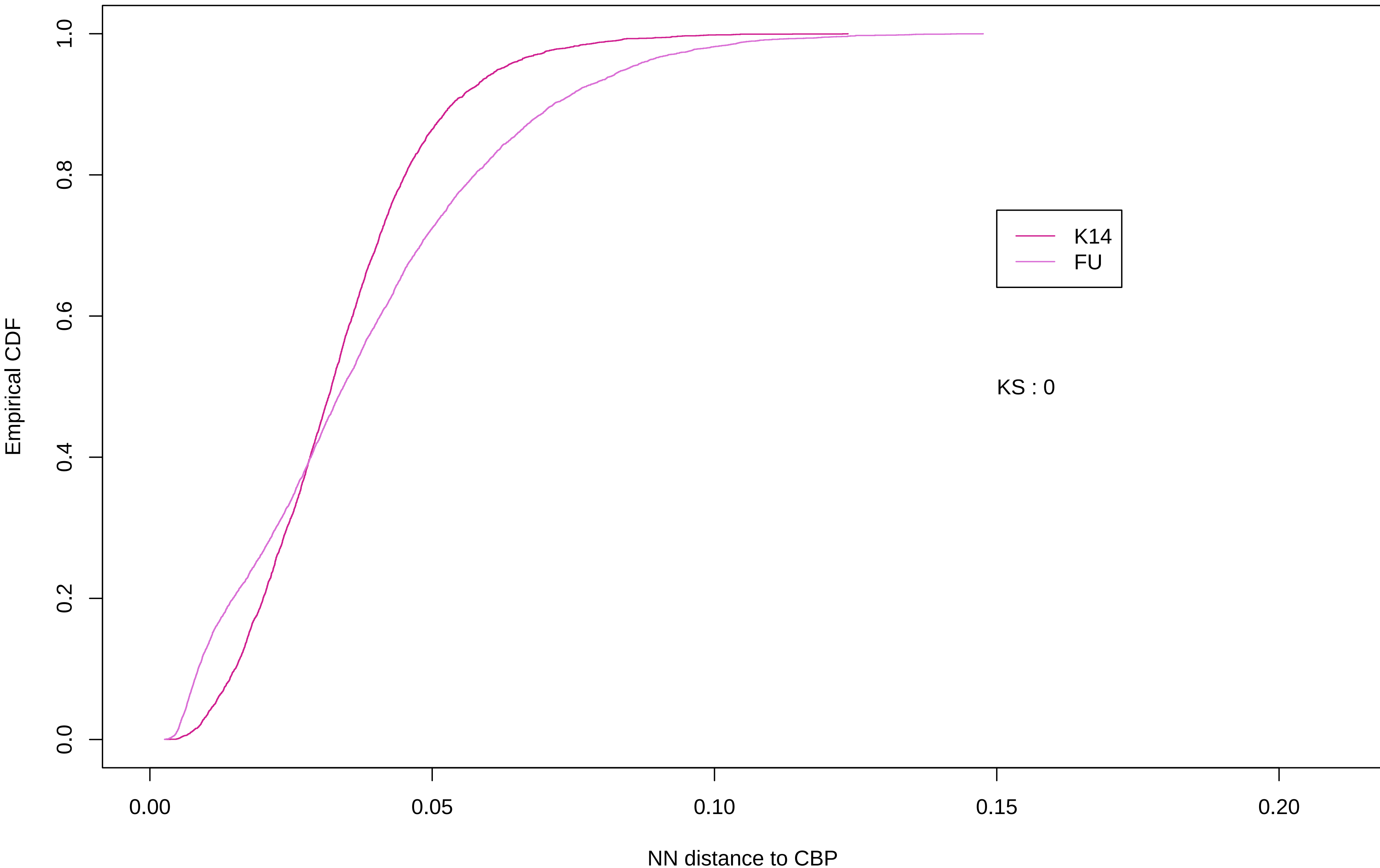

Empirical CDF
NN distance to CBP
K14
FU
KS : 0
0.0
0.2
0.4
0.6
0.8
1.0
0.00
0.05
0.10
0.15
0.20

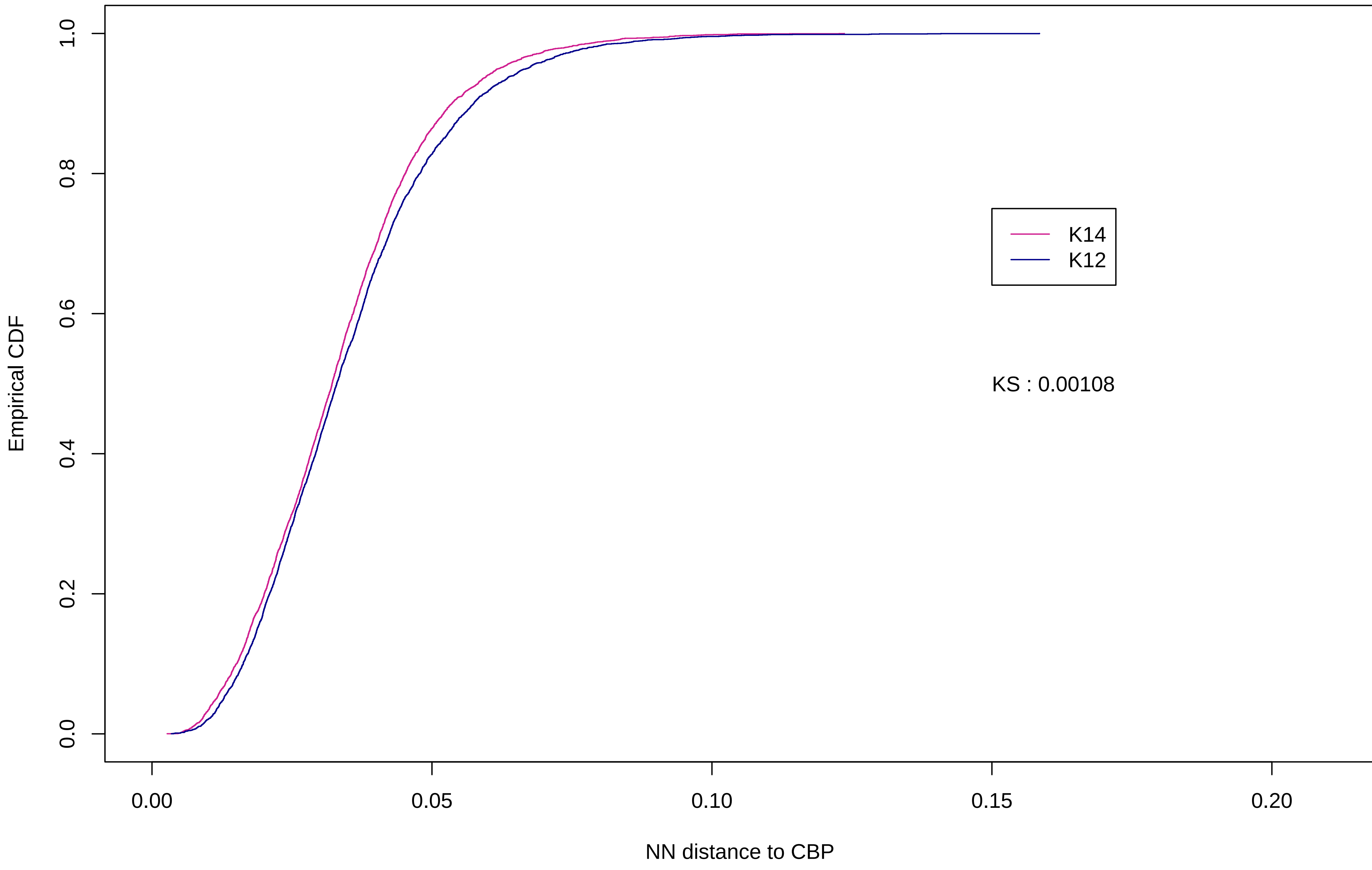

K14
K12
KS : 0.00108
Empirical CDF
0.0
0.2
0.4
0.6
0.8
1.0
0.00
0.05
0.10
0.15
0.20
NN distance to CBP

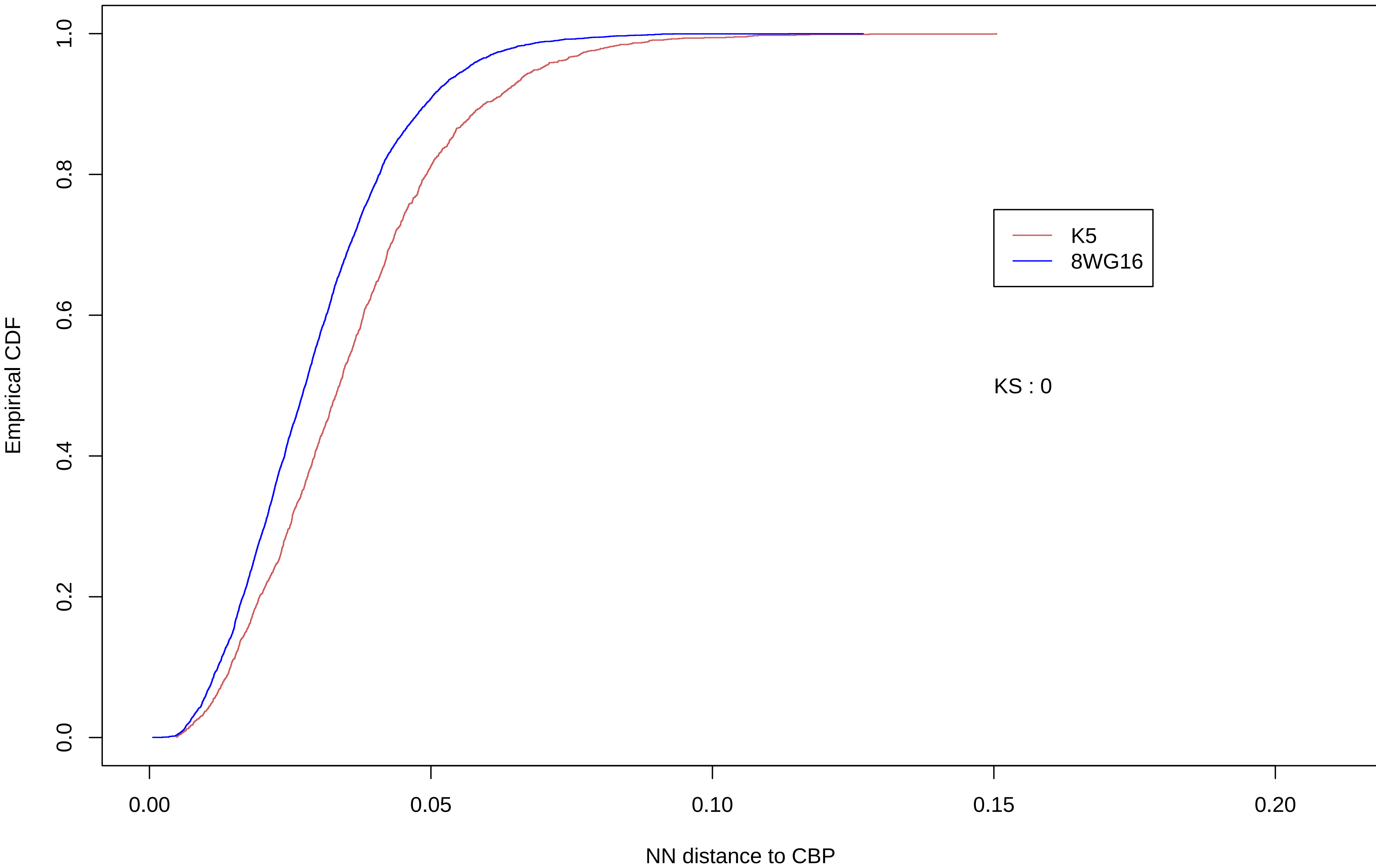

Empirical CDF
0.0
0.2
0.4
0.6
0.8
1.0
0.00
0.05
0.10
0.15
0.20
NN distance to CBP
K5
8WG16
KS : 0

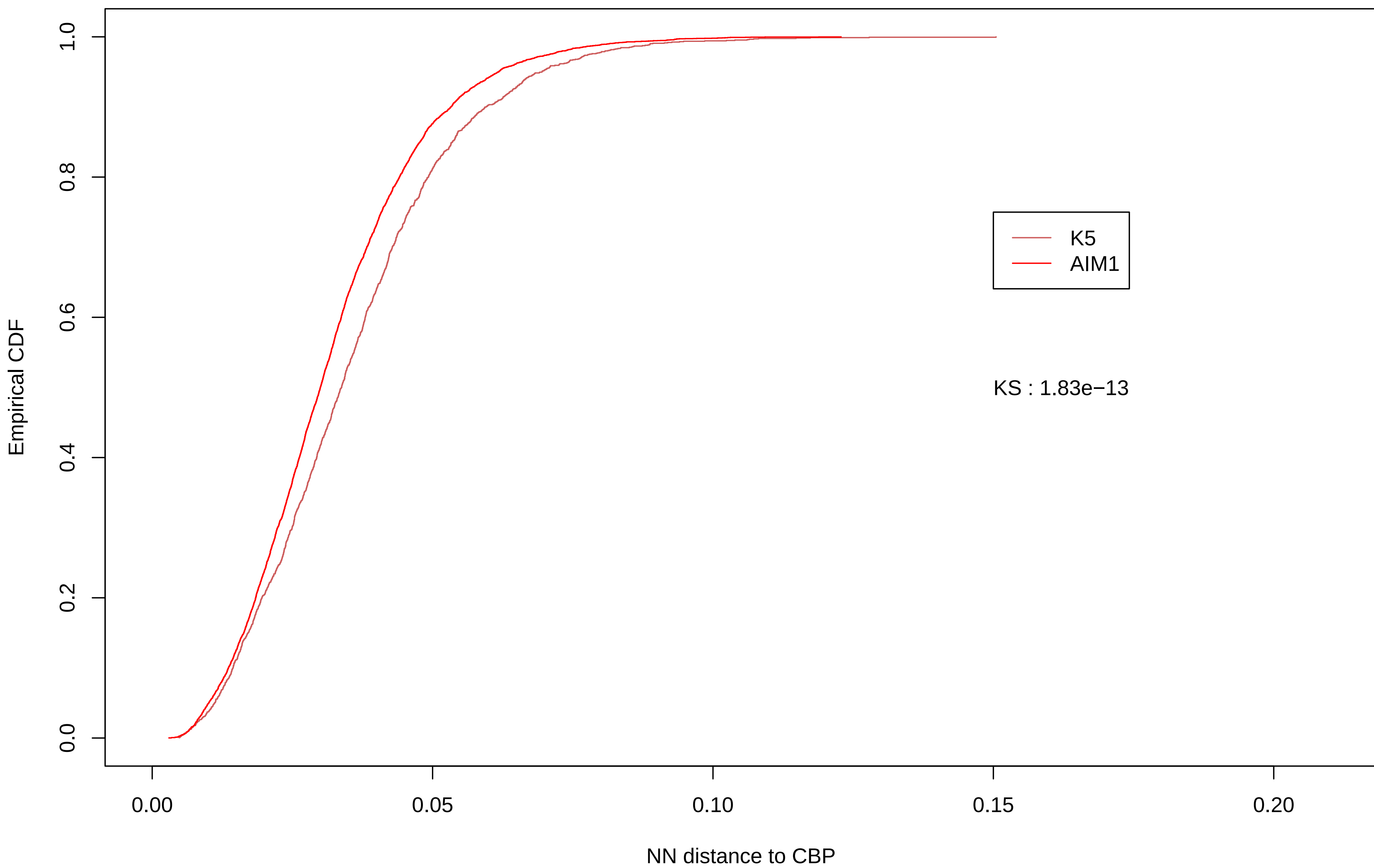
Empirical CDF
0.0
0.2
0.4
0.6
0.8
1.0
0.00
0.05
0.10
0.15
0.20
NN distance to CBP
K5
AIM1
KS : 1.83e−13

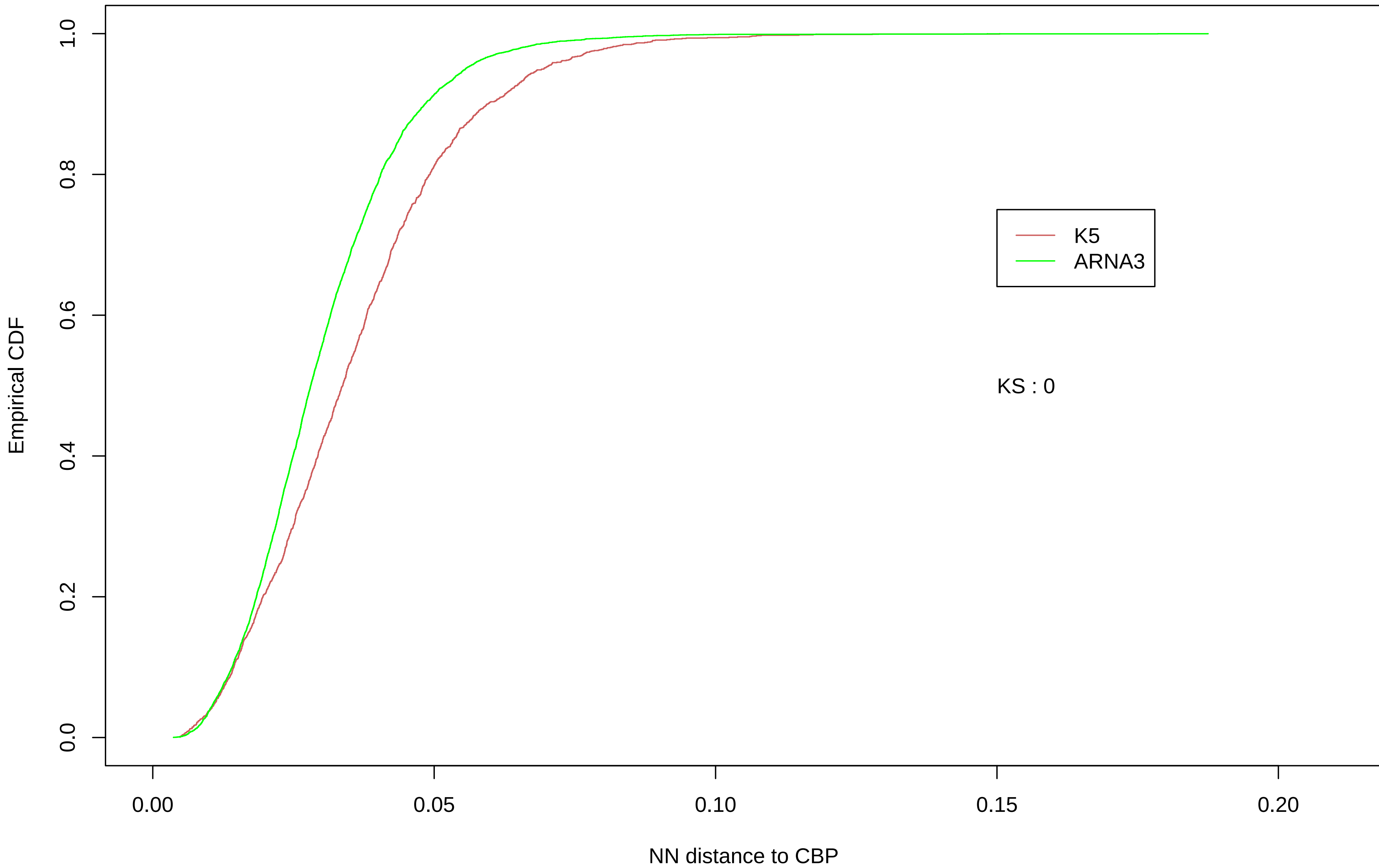

Empirical CDF
NN distance to CBP
K5
ARNA3
KS : 0
0.0
0.2
0.4
0.6
0.8
1.0
0.00
0.05
0.10
0.15
0.20

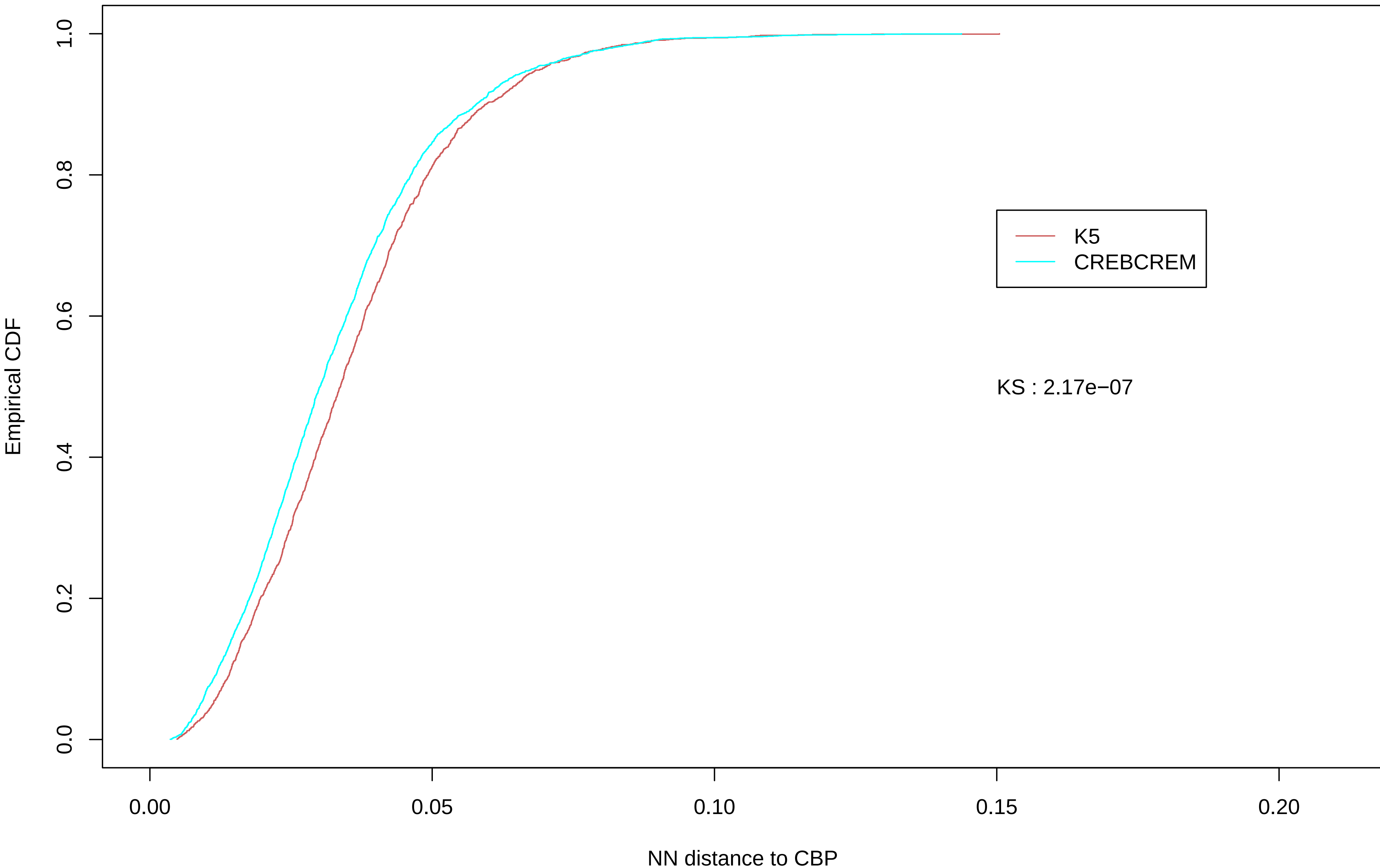

Empirical CDF
0.0
0.2
0.4
0.6
0.8
1.0
0.00
0.05
0.10
0.15
0.20
NN distance to CBP
K5
CREBCREM
KS : 2.17e−07

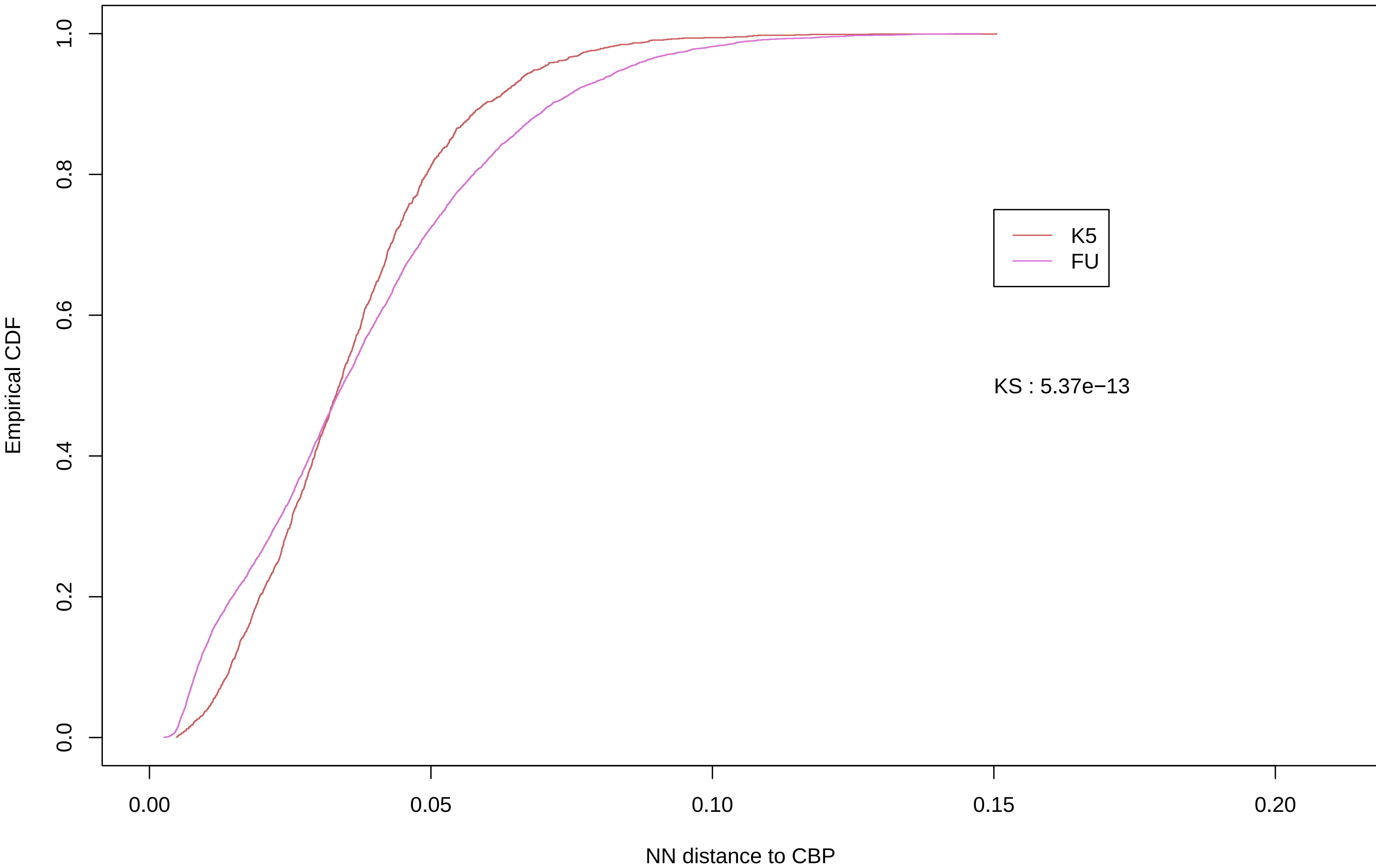
Empirical CDF
0.0
0.2
0.4
0.6
0.8
1.0
0.00
0.05
0.10
0.15
0.20
NN distance to CBP
K5
FU
KS : 5.37e−13

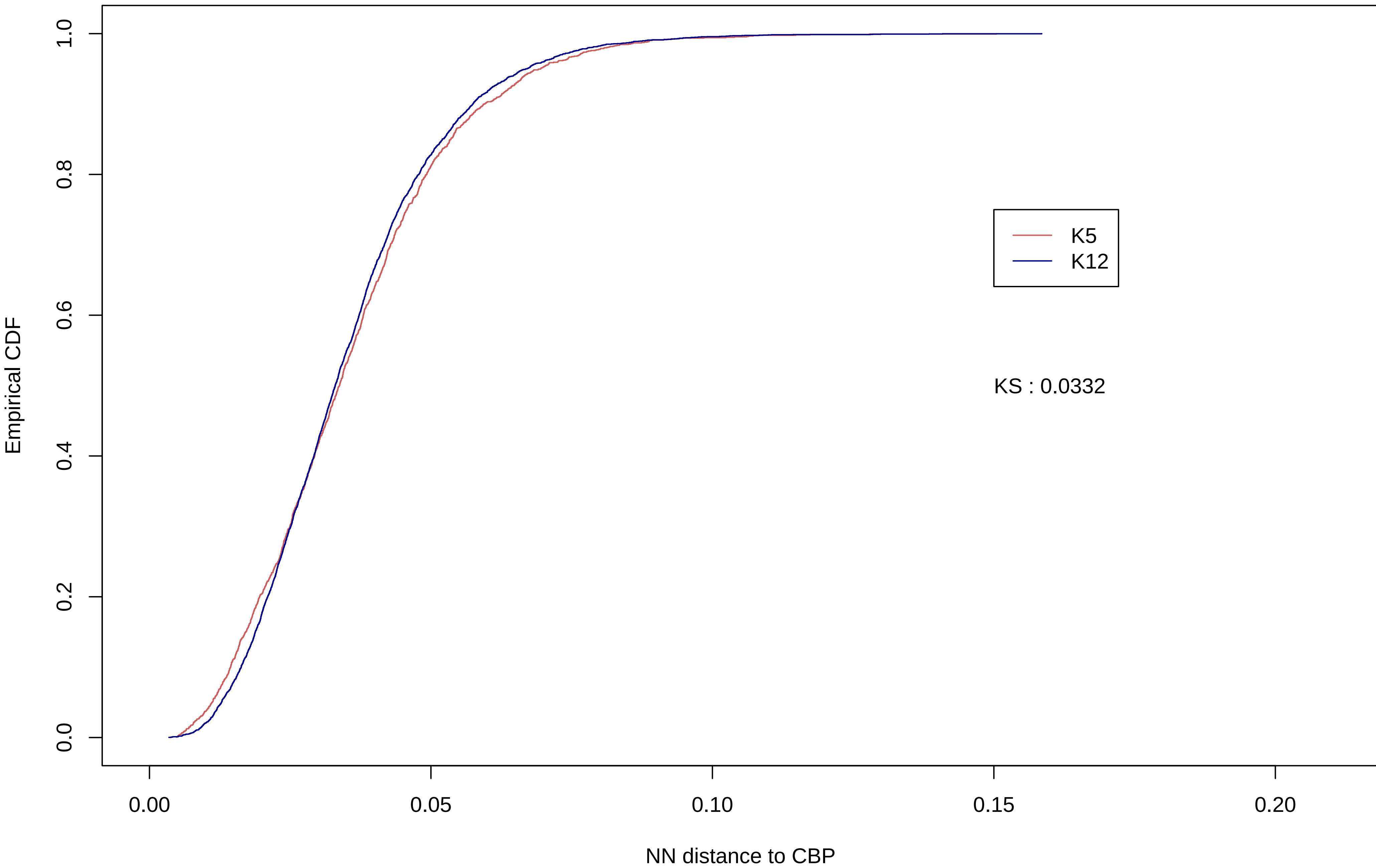
Empirical CDF
NN distance to CBP
K5
K12
KS : 0.0332
0.0
0.2
0.4
0.6
0.8
1.0
0.00
0.05
0.10
0.15
0.20

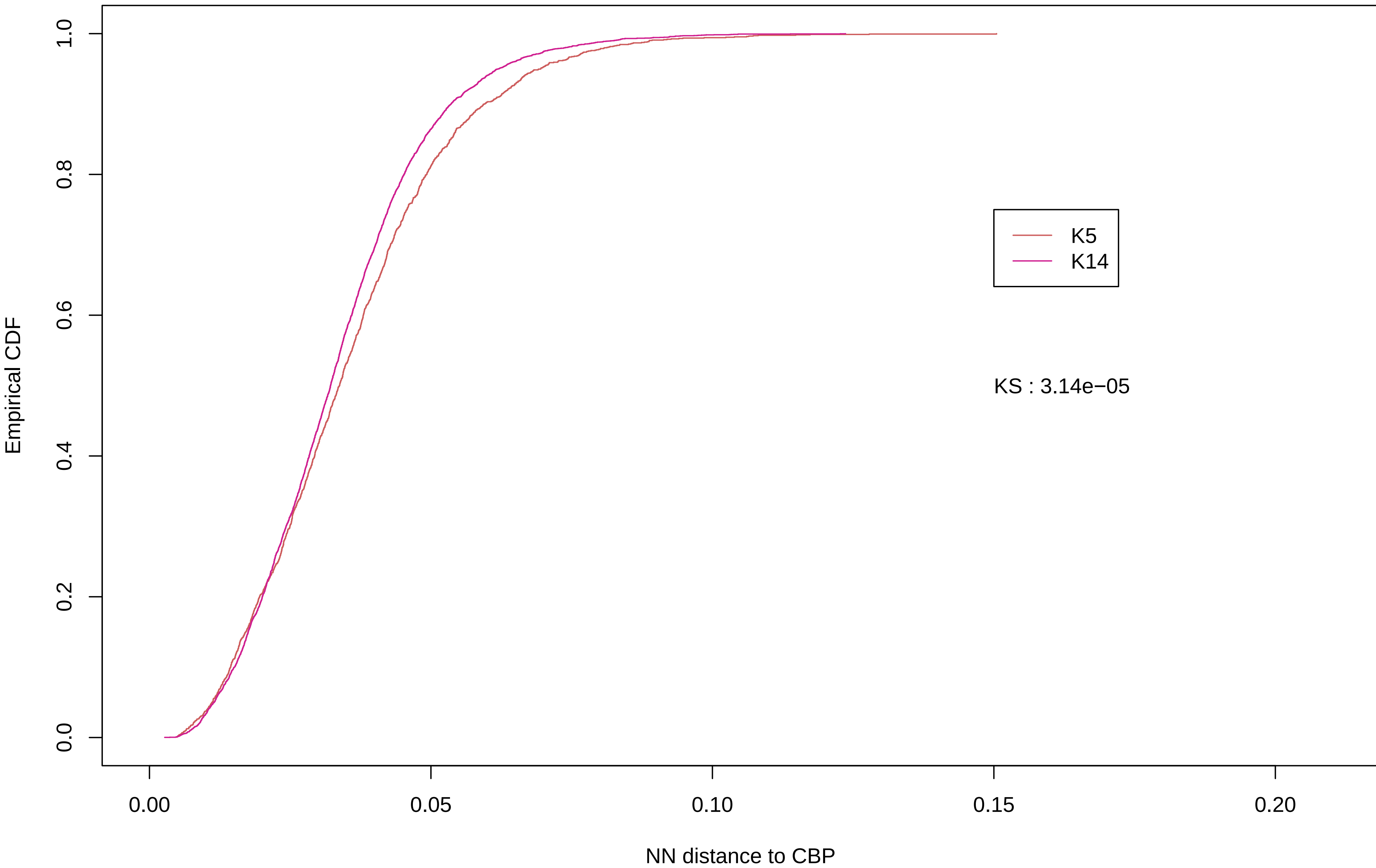

Empirical CDF
0.0
0.2
0.4
0.6
0.8
1.0
0.00
0.05
0.10
0.15
0.20
NN distance to CBP
K5
K14
KS : 3.14e−05

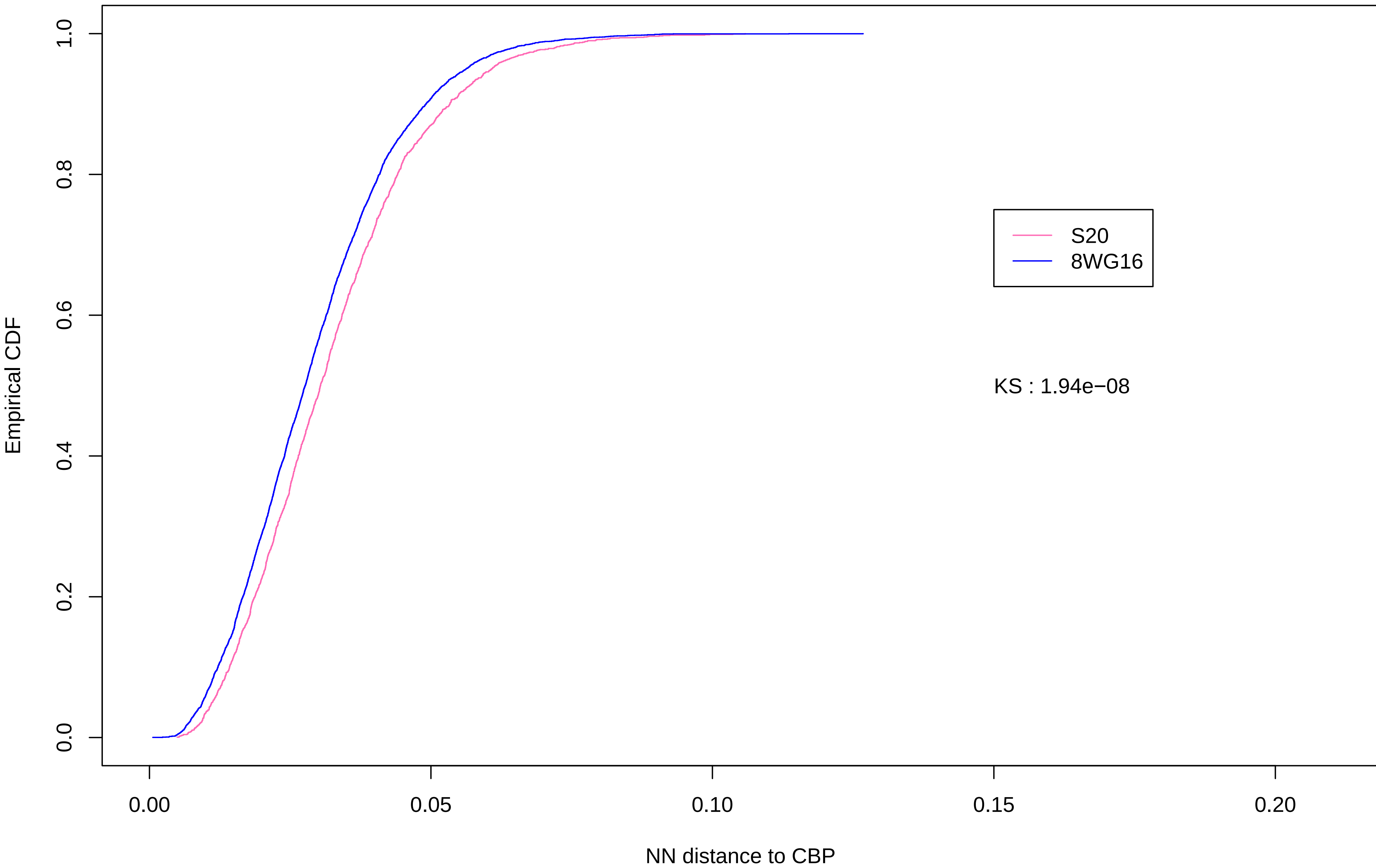

Empirical CDF
0.0
0.2
0.4
0.6
0.8
1.0
0.00
0.05
0.10
0.15
0.20
NN distance to CBP
S20
8WG16
KS : 1.94e−08

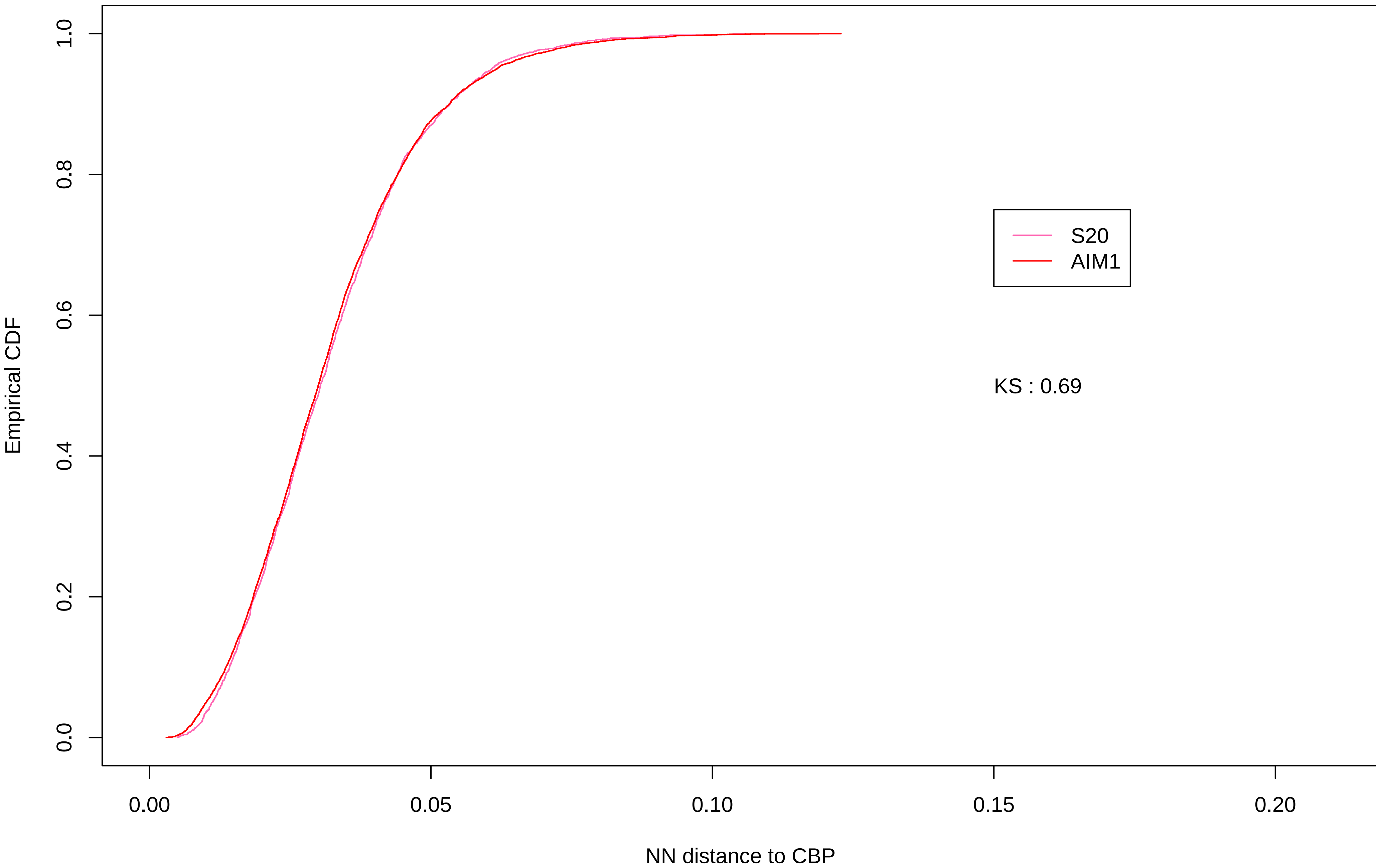

Empirical CDF
NN distance to CBP
0.0
0.2
0.4
0.6
0.8
1.0
0.00
0.05
0.10
0.15
0.20
S20
AIM1
KS : 0.69

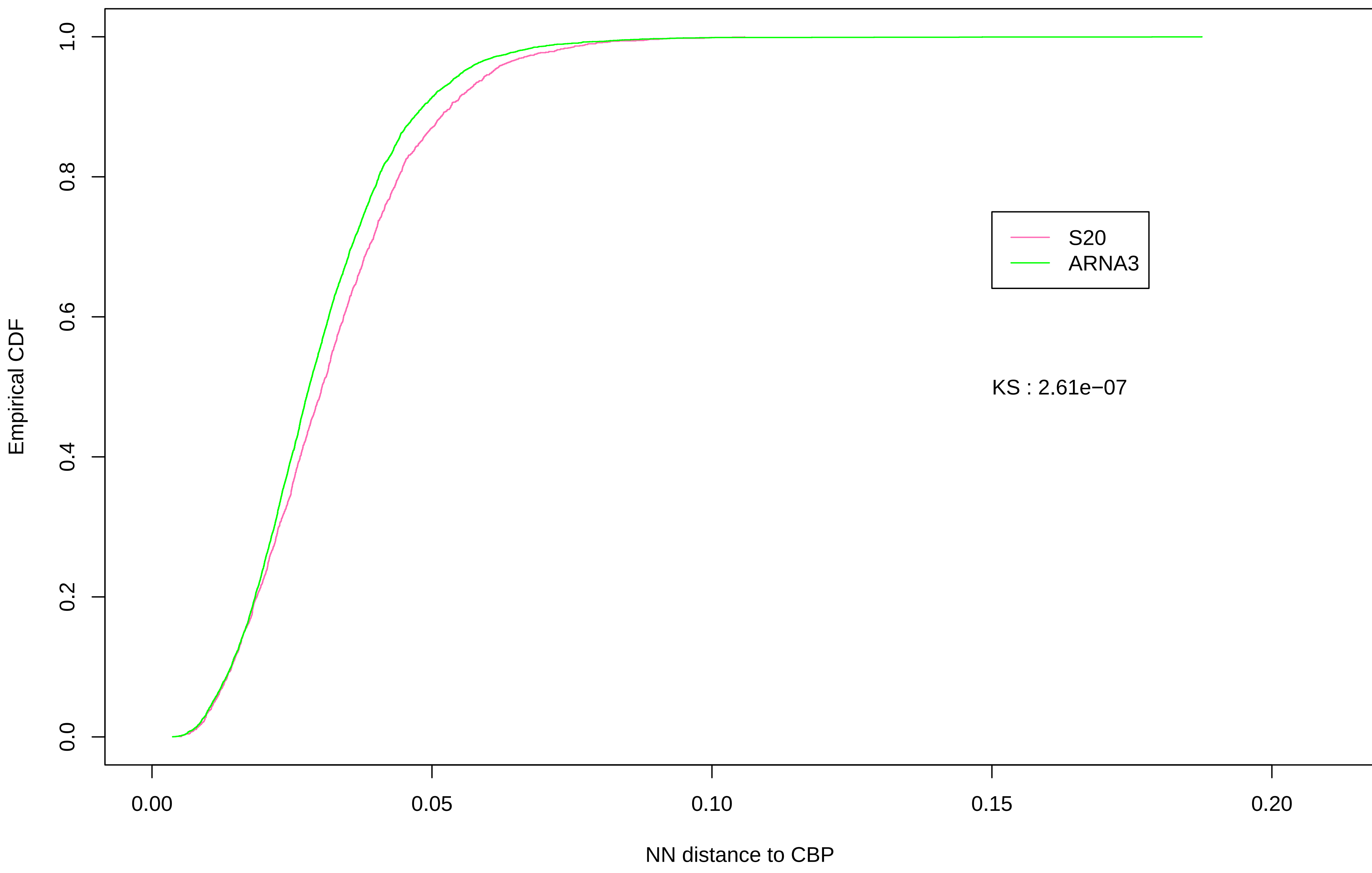

Empirical CDF
NN distance to CBP
S20
ARNA3
KS : 2.61e−07
0.0
0.2
0.4
0.6
0.8
1.0
0.00
0.05
0.10
0.15
0.20

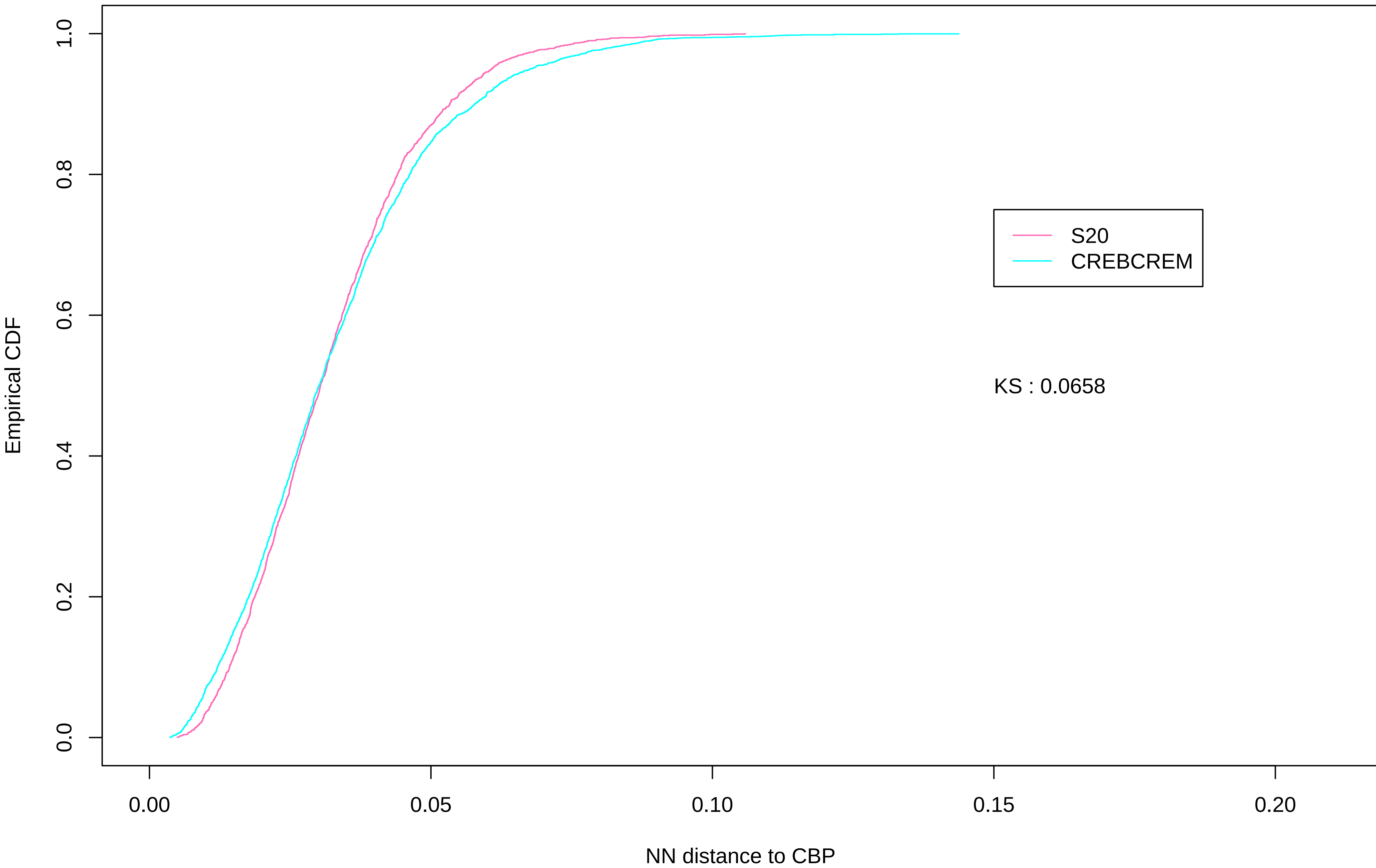

S20
CREBCREM
KS : 0.0658
Empirical CDF
0.0
0.2
0.4
0.6
0.8
1.0
0.00
0.05
0.10
0.15
0.20
NN distance to CBP

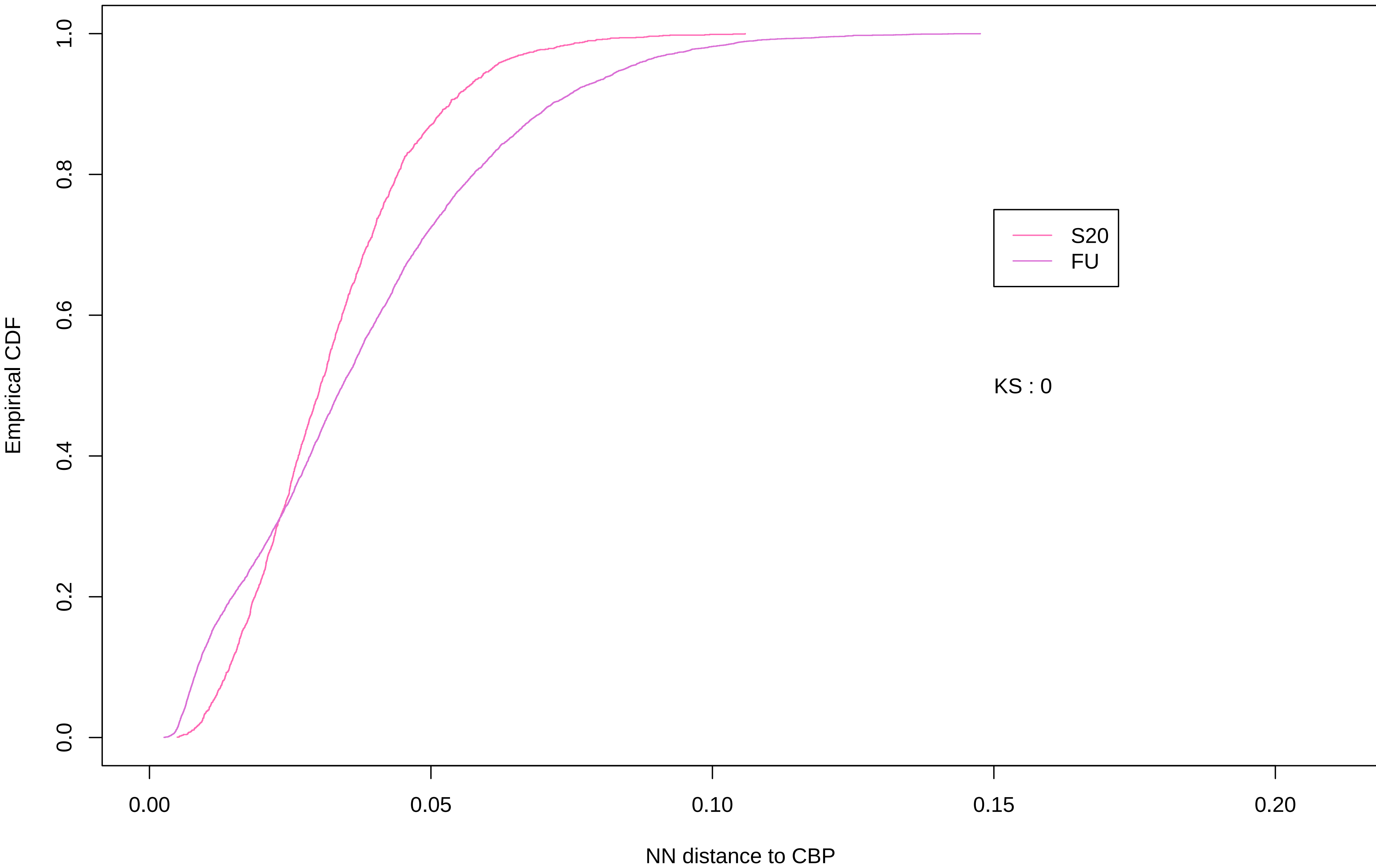

Empirical CDF
0.0
0.2
0.4
0.6
0.8
1.0
0.00
0.05
0.10
0.15
0.20
NN distance to CBP
S20
FU
KS : 0

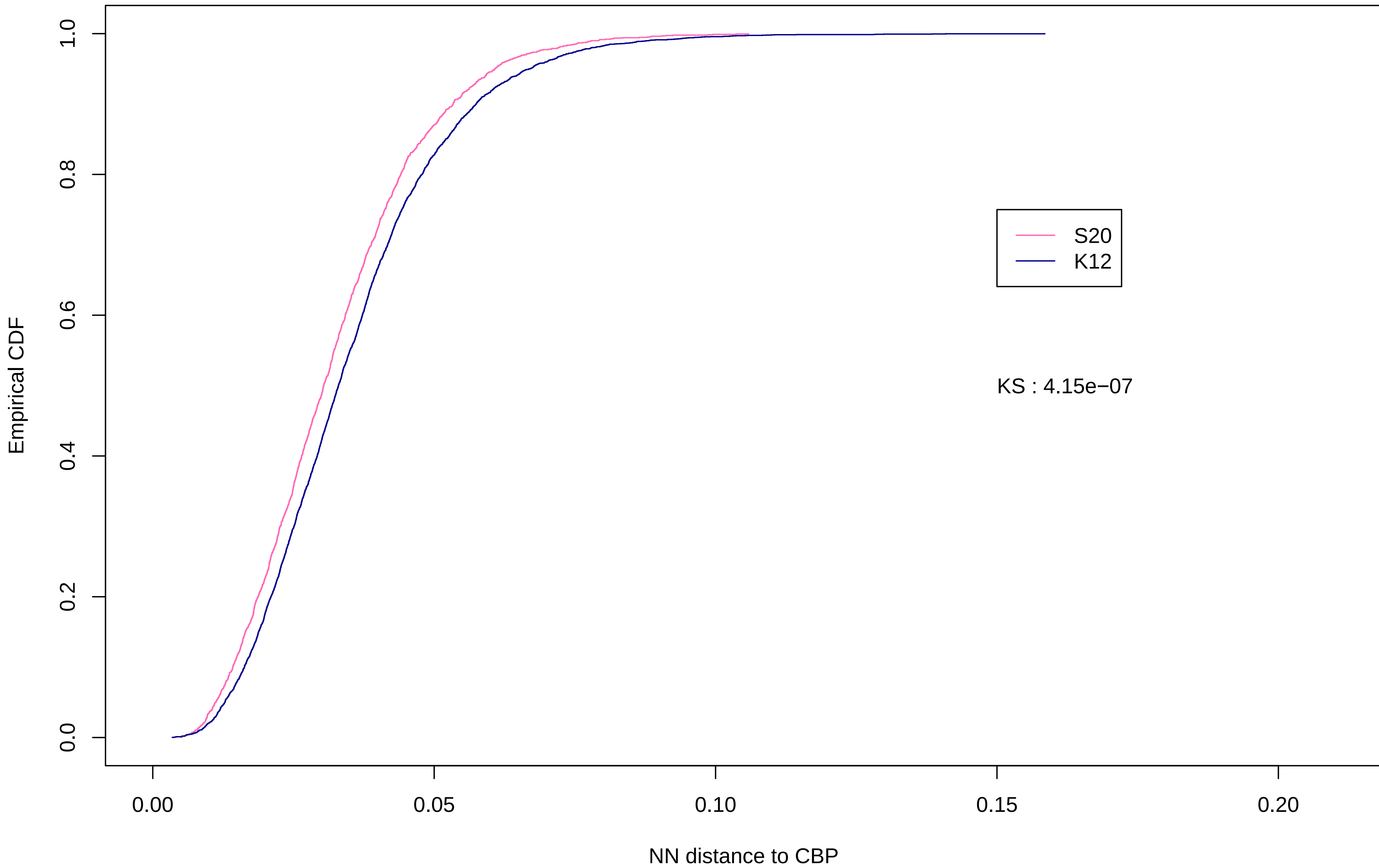

Empirical CDF
0.0
0.2
0.4
0.6
0.8
1.0
0.00
0.05
0.10
0.15
0.20
NN distance to CBP
S20
K12
KS : 4.15e−07

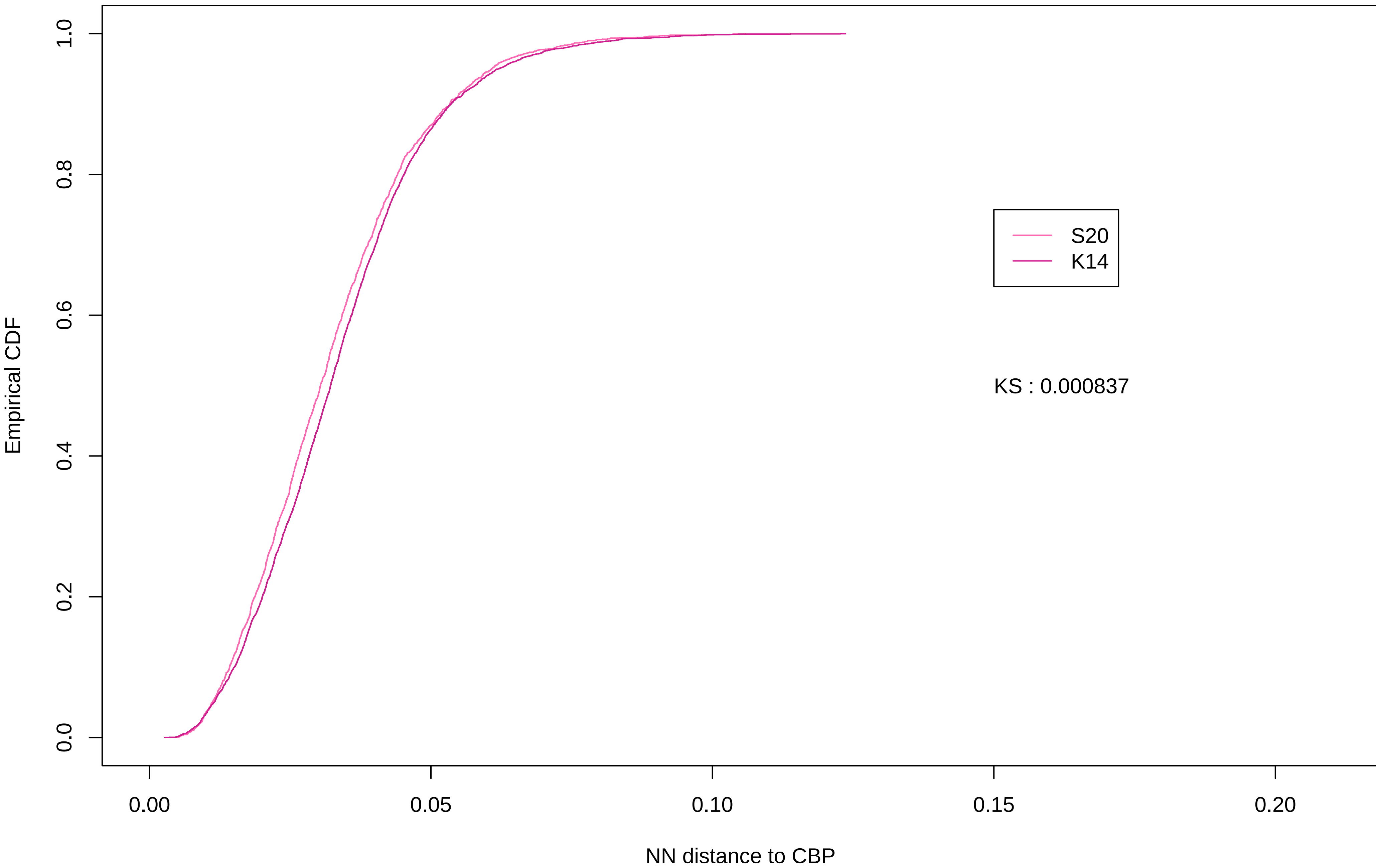

Empirical CDF
0.0
0.2
0.4
0.6
0.8
1.0
0.00
0.05
0.10
0.15
0.20
NN distance to CBP
S20
K14
KS : 0.000837

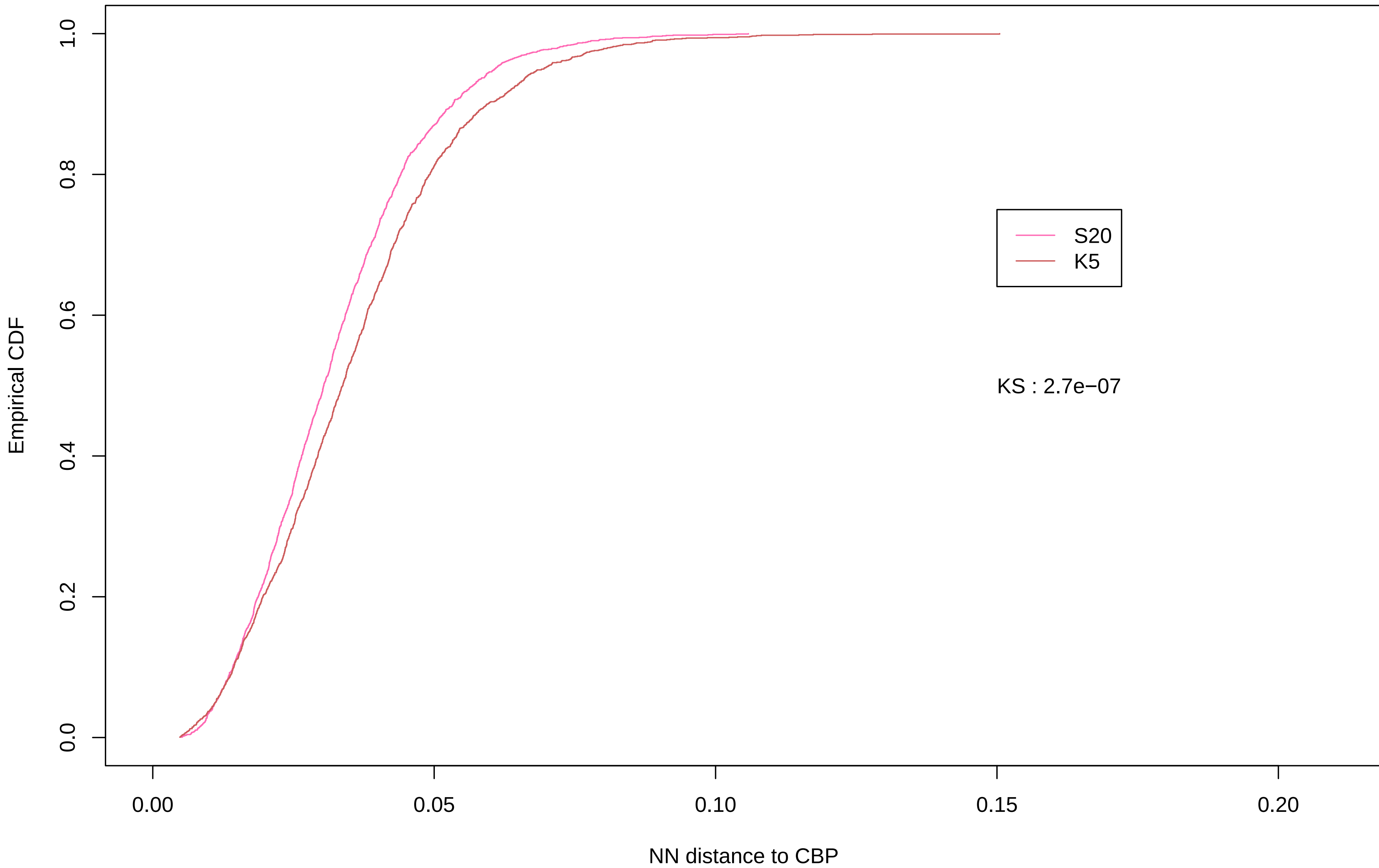

Empirical CDF
0.0
0.2
0.4
0.6
0.8
1.0
0.00
0.05
0.10
0.15
0.20
NN distance to CBP
S20
K5
KS : 2.7e−07

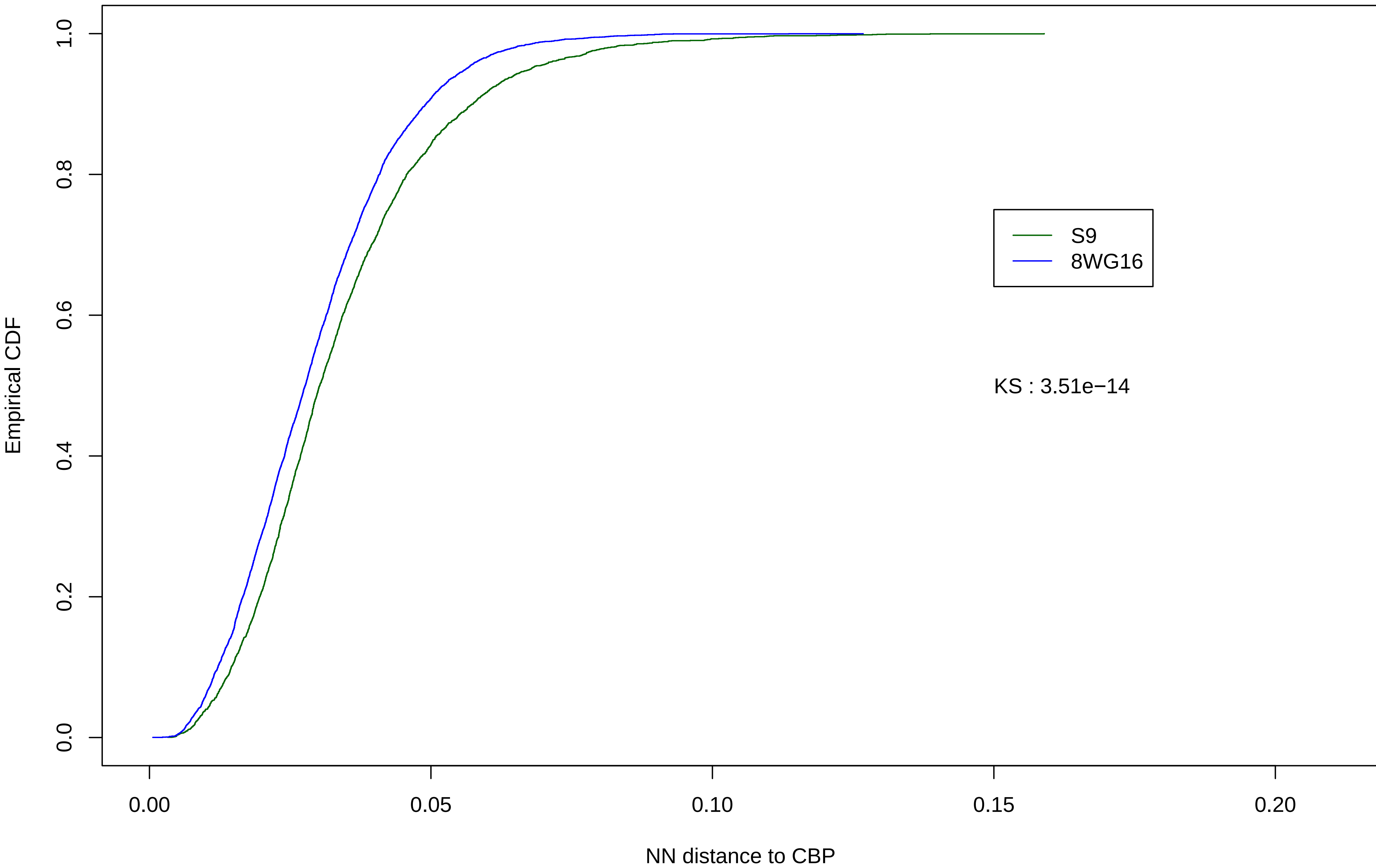

Empirical CDF
0.0
0.2
0.4
0.6
0.8
1.0
0.00
0.05
0.10
0.15
0.20
NN distance to CBP
S9
8WG16
KS : 3.51e−14

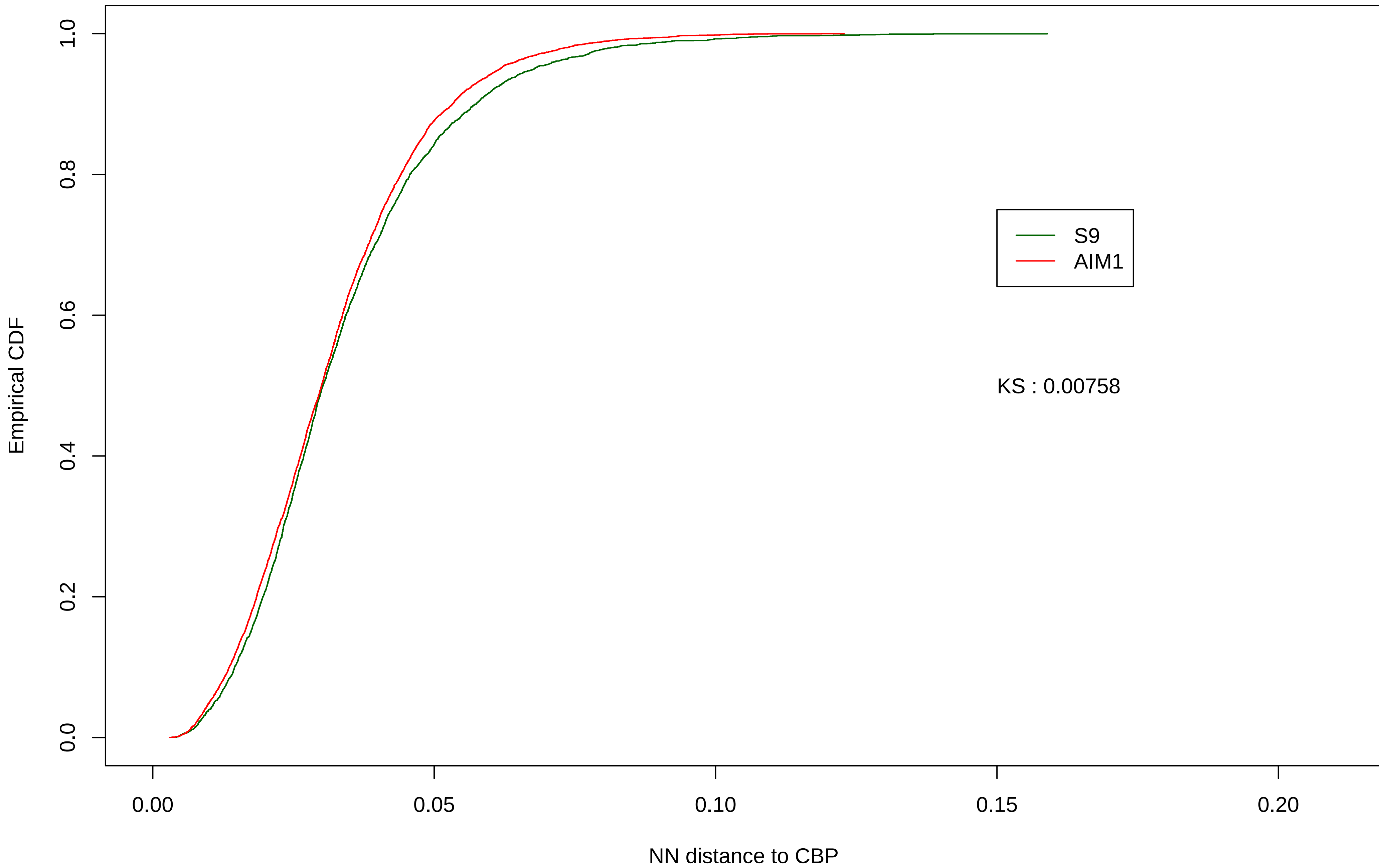
S9
AIM1
KS : 0.00758
Empirical CDF
NN distance to CBP
0.0
0.2
0.4
0.6
0.8
1.0
0.00
0.05
0.10
0.15
0.20

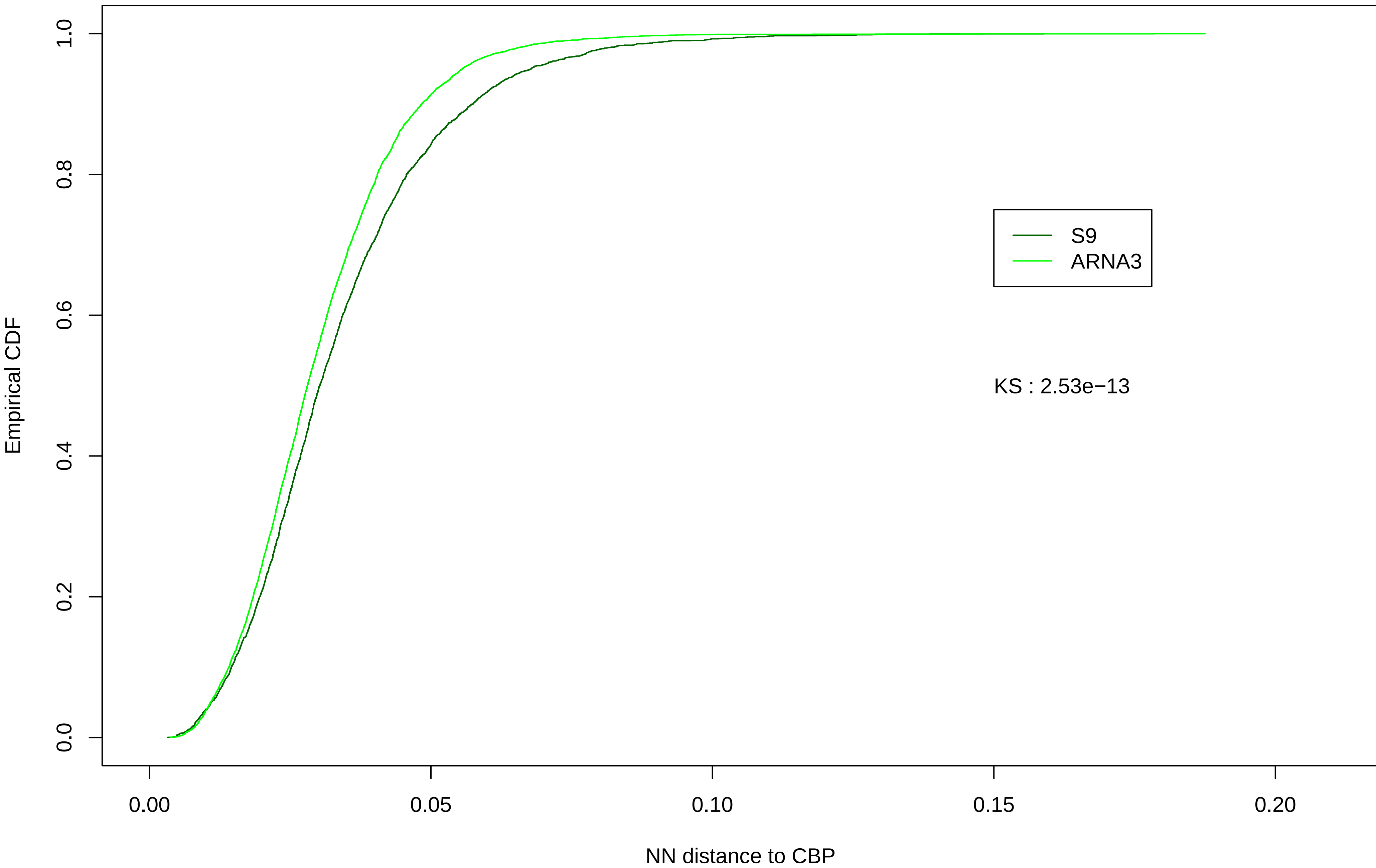

Empirical CDF
0.0
0.2
0.4
0.6
0.8
1.0
0.00
0.05
0.10
0.15
0.20
NN distance to CBP
S9
ARNA3
KS : 2.53e−13

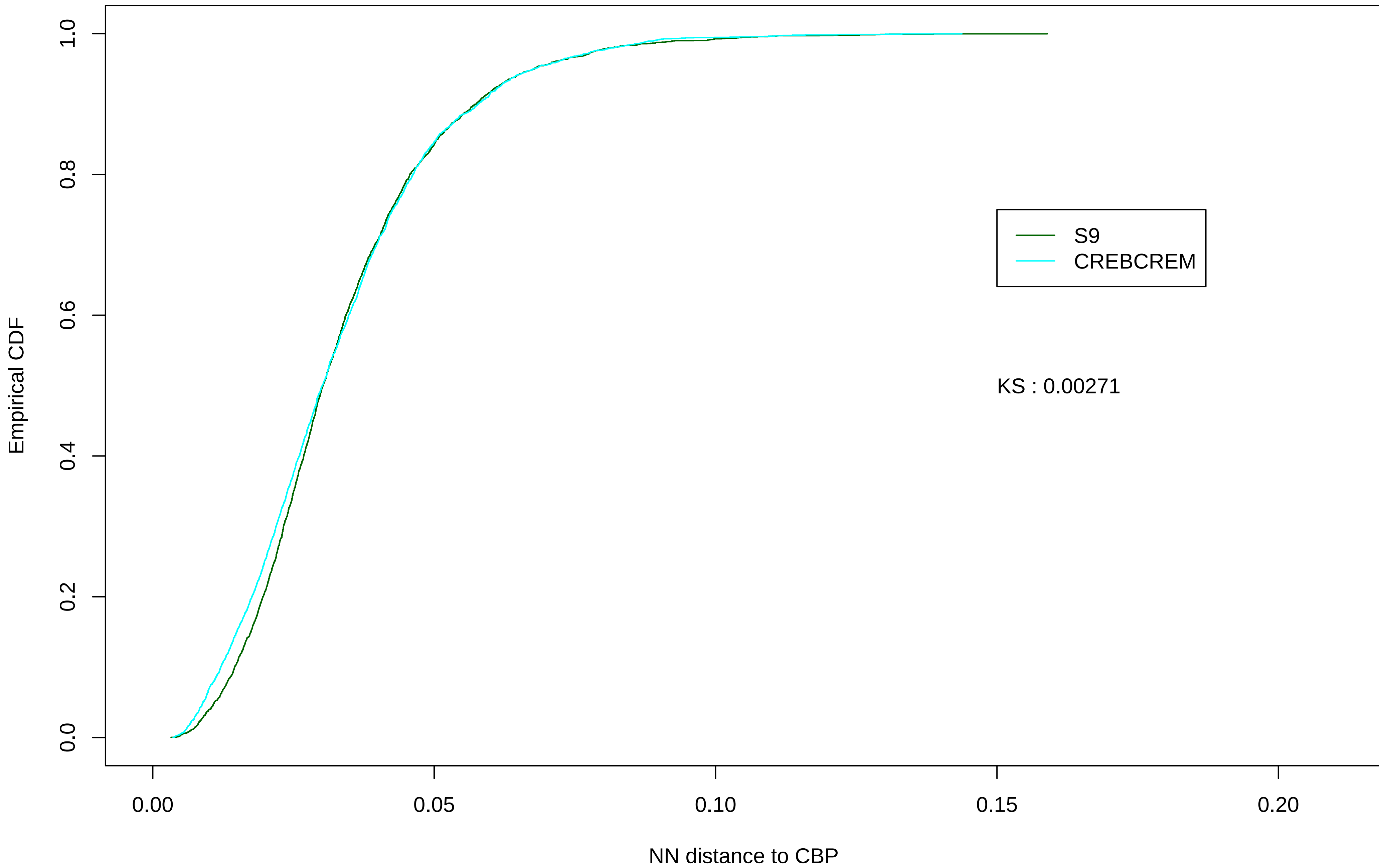

Empirical CDF
0.0
0.2
0.4
0.6
0.8
1.0
0.00
0.05
0.10
0.15
0.20
NN distance to CBP
S9
CREBCREM
KS : 0.00271

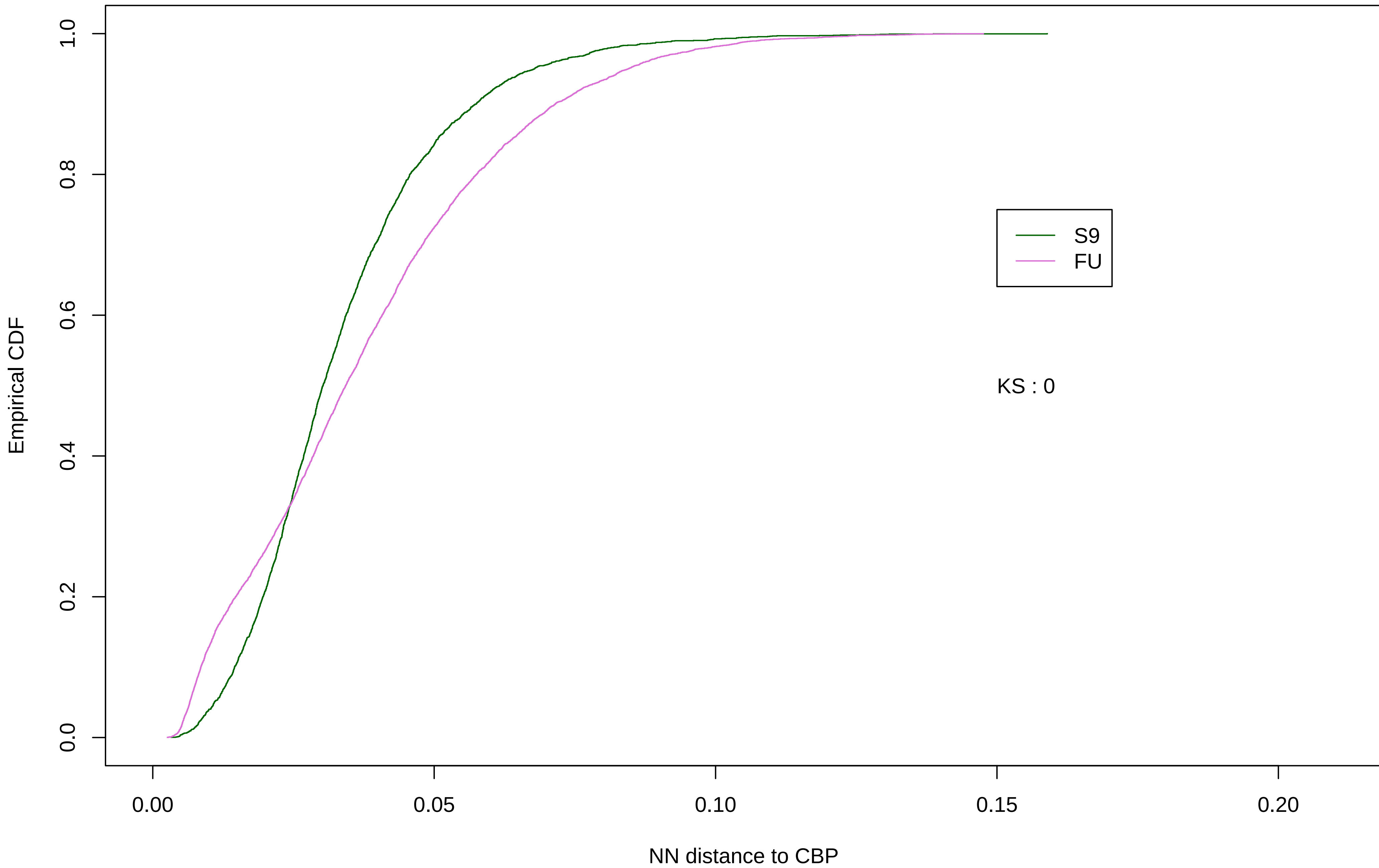
Empirical CDF
0.0
0.2
0.4
0.6
0.8
1.0
0.00
0.05
0.10
0.15
0.20
NN distance to CBP
S9
FU
KS : 0

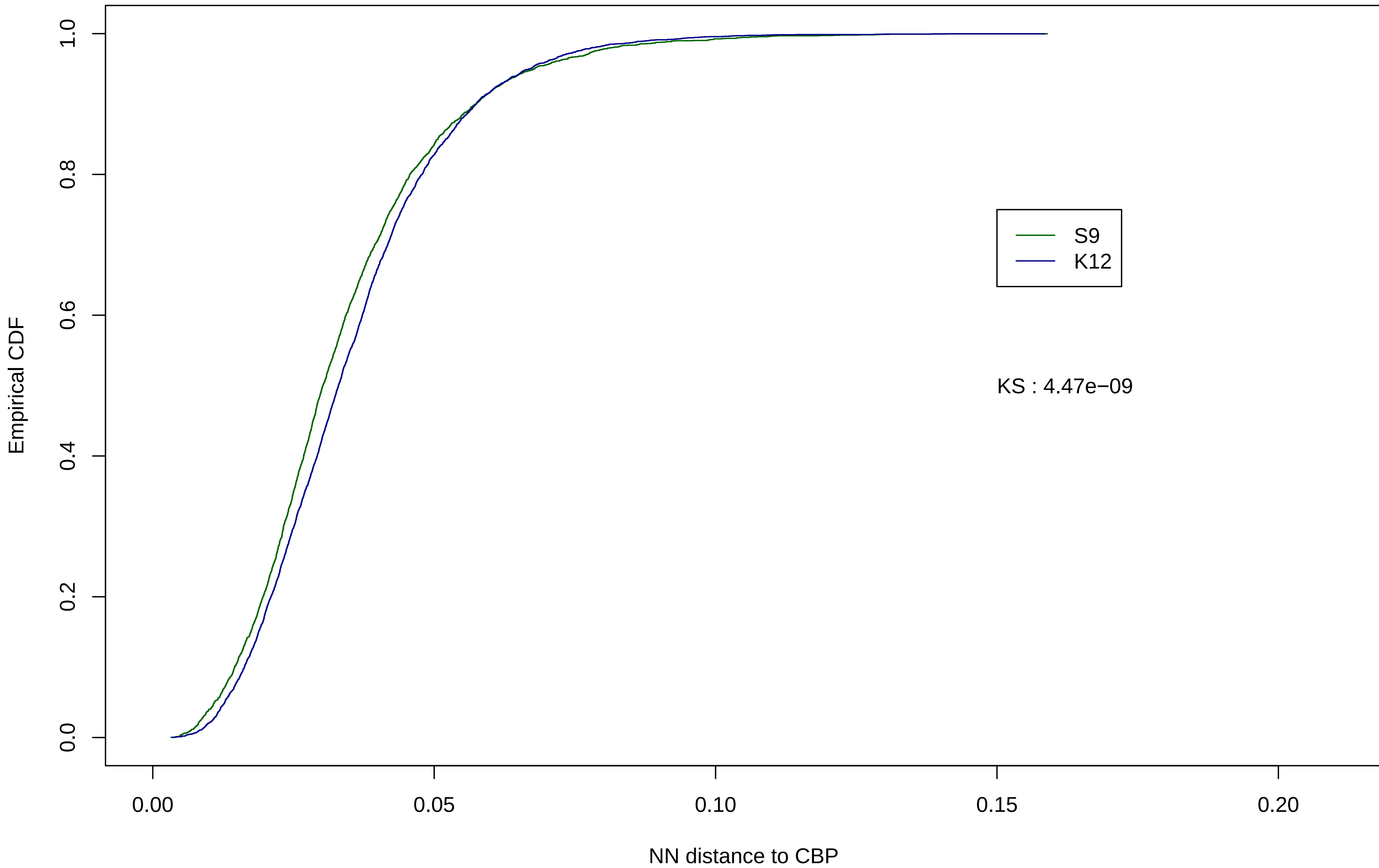

Empirical CDF
1.0
0.8
0.6
0.4
0.2
0.0
0.00
0.05
0.10
0.15
0.20
NN distance to CBP
S9
K12
KS : 4.47e−09

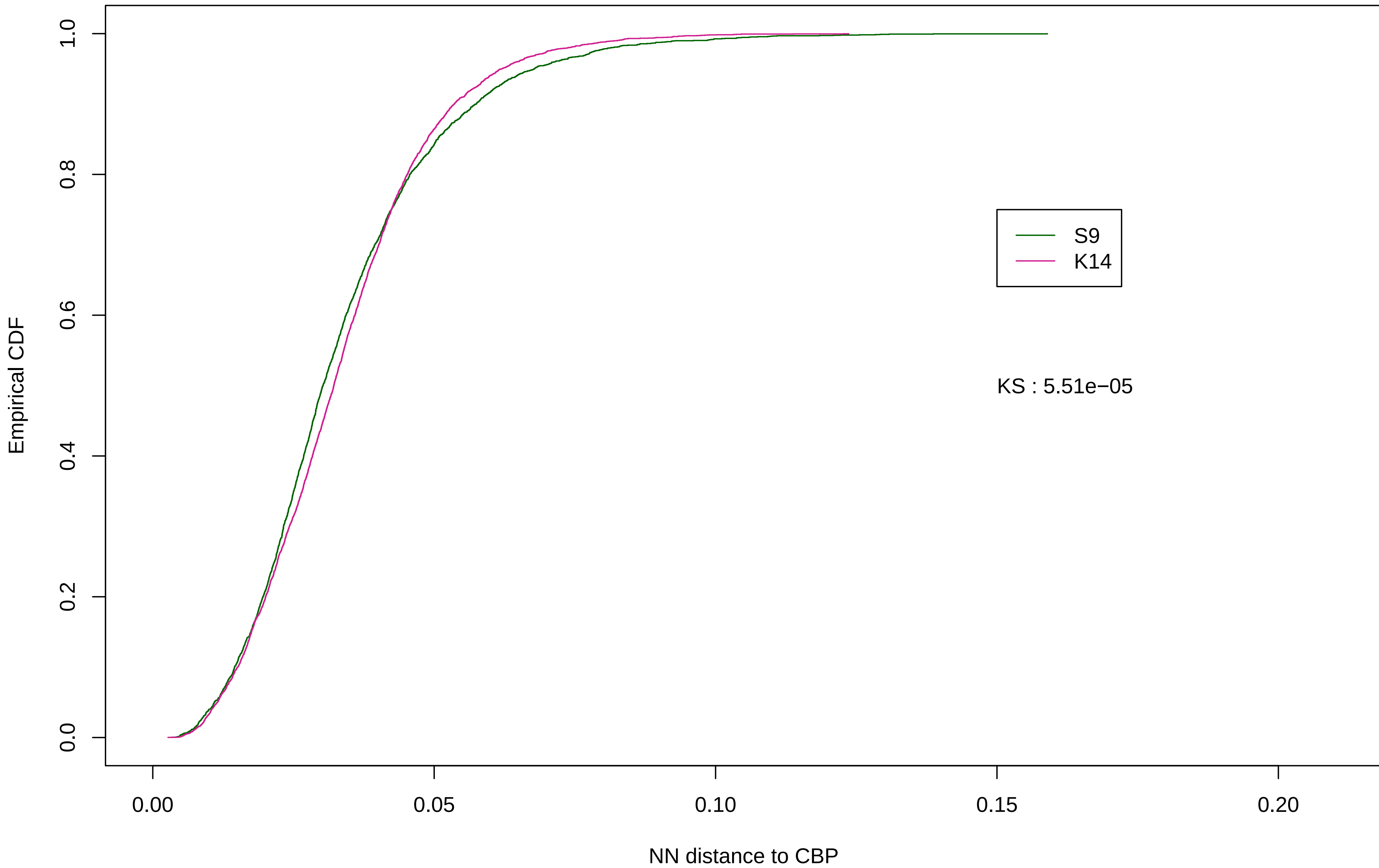

Empirical CDF
0.0
0.2
0.4
0.6
0.8
1.0
0.00
0.05
0.10
0.15
0.20
NN distance to CBP
S9
K14
KS : 5.51e−05

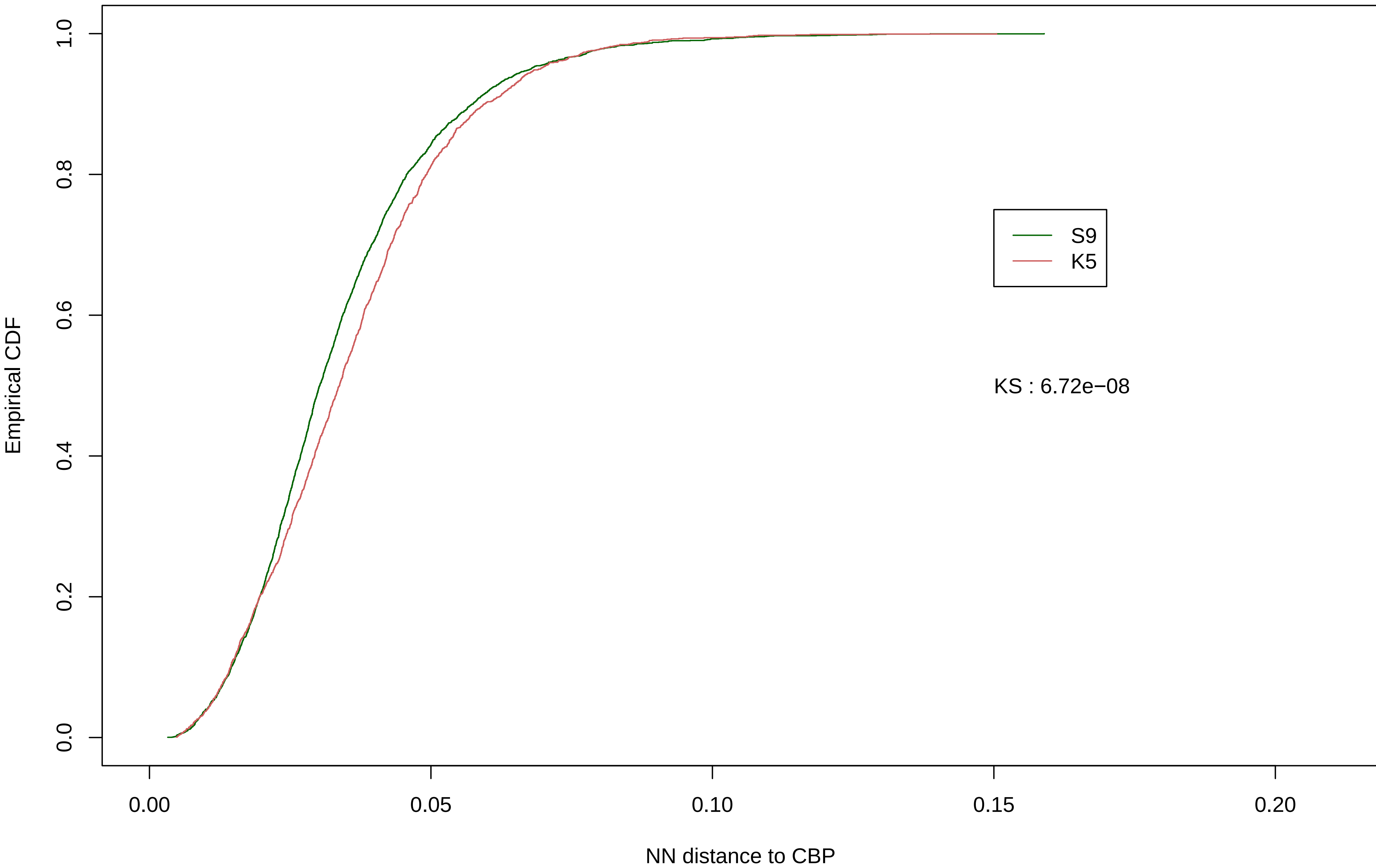

S9
K5
KS : 6.72e−08
Empirical CDF
NN distance to CBP
0.0
0.2
0.4
0.6
0.8
1.0
0.00
0.05
0.10
0.15
0.20

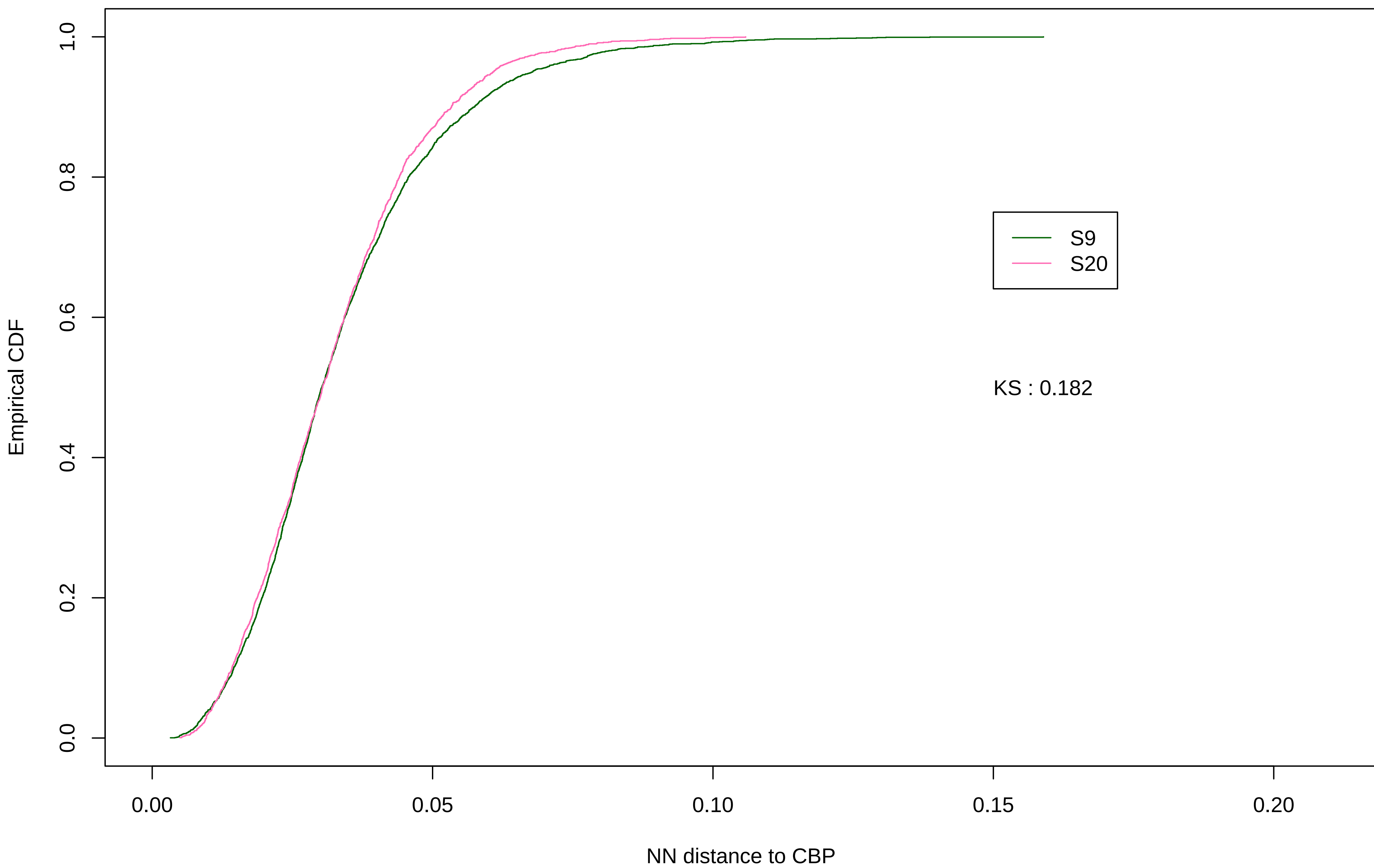

Empirical CDF
0.0
0.2
0.4
0.6
0.8
1.0
0.00
0.05
0.10
0.15
0.20
NN distance to CBP
S9
S20
KS : 0.182

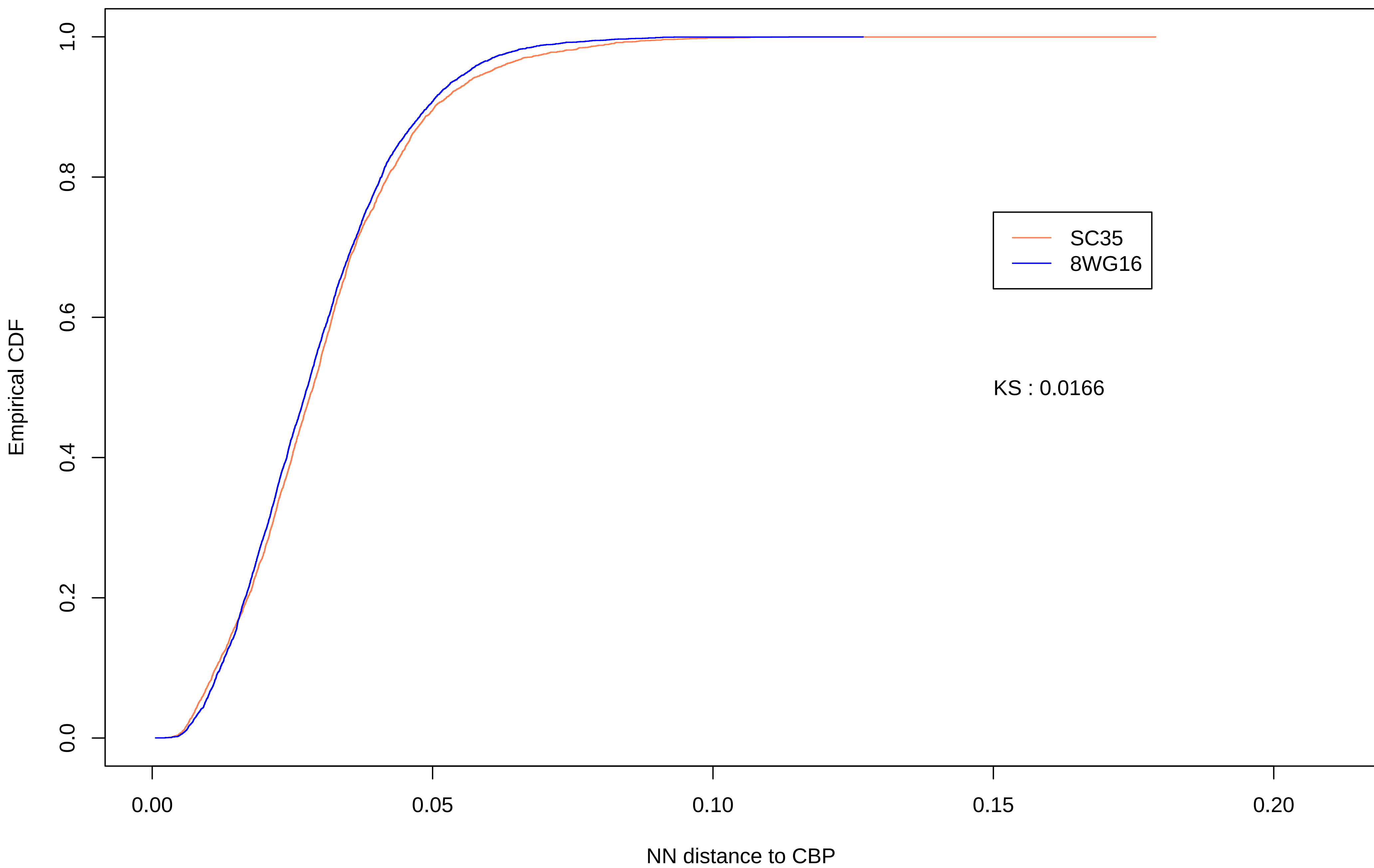

Empirical CDF
1.0
0.8
0.6
0.4
0.2
0.0
SC35
8WG16
KS : 0.0166
0.00
0.05
0.10
0.15
0.20
NN distance to CBP

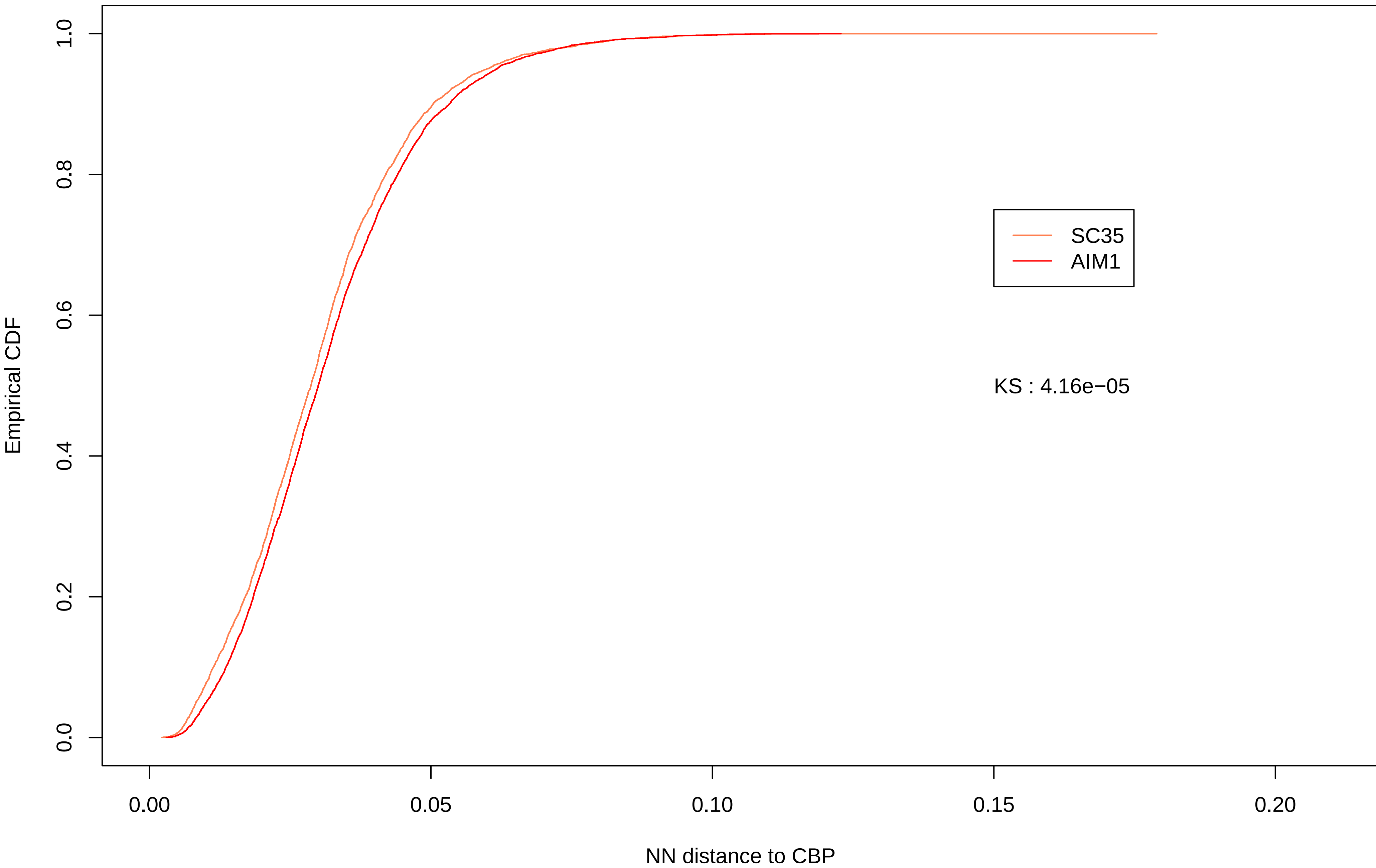

Empirical CDF
0.0
0.2
0.4
0.6
0.8
1.0
0.00
0.05
0.10
0.15
0.20
NN distance to CBP
SC35
AIM1
KS : 4.16e−05

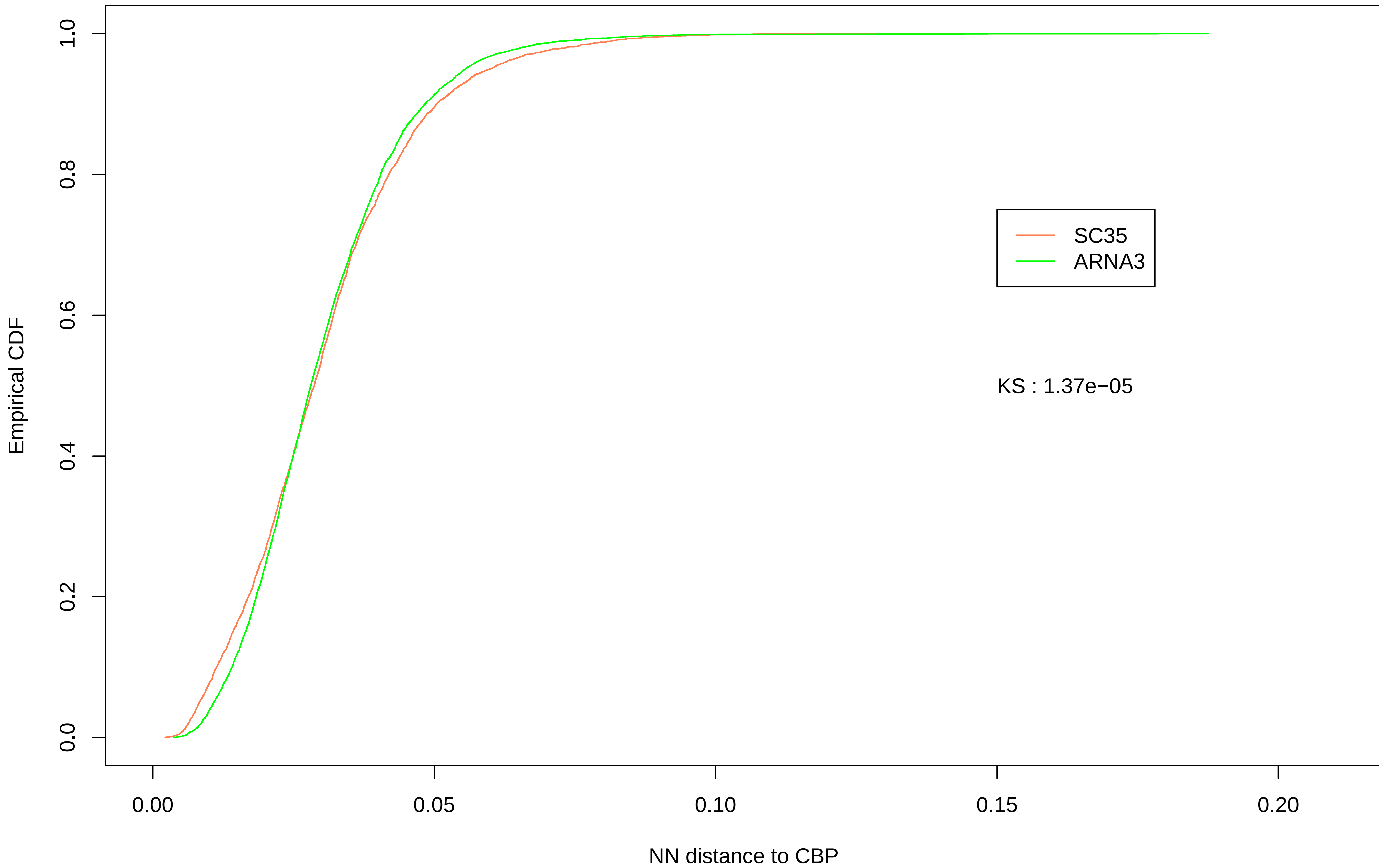

Empirical CDF
1.0
0.8
0.6
0.4
0.2
0.0
0.00
0.05
0.10
0.15
0.20
NN distance to CBP
SC35
ARNA3
KS : 1.37e−05

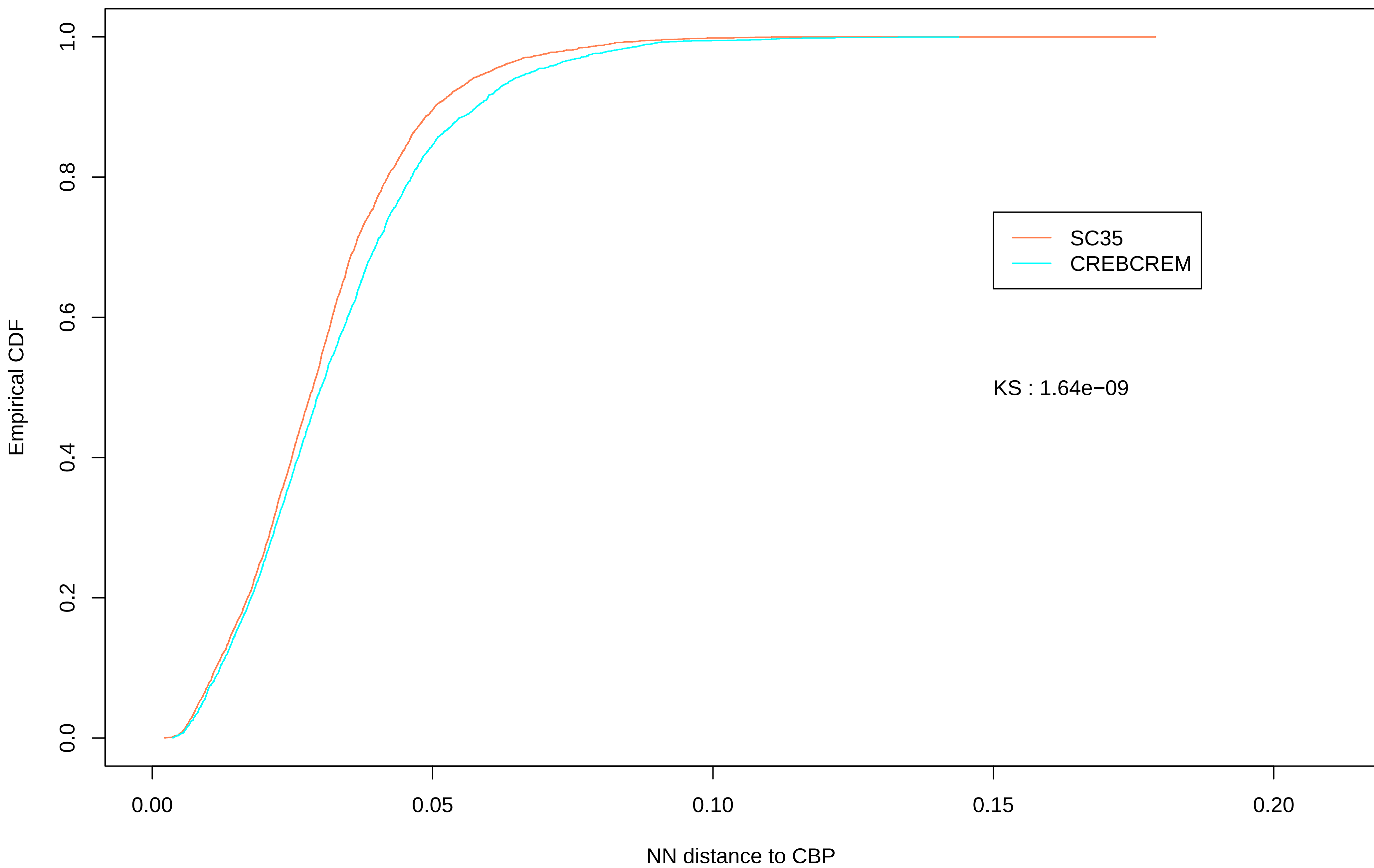

SC35
CREBCREM
KS : 1.64e−09
Empirical CDF
NN distance to CBP
0.0
0.2
0.4
0.6
0.8
1.0
0.00
0.05
0.10
0.15
0.20

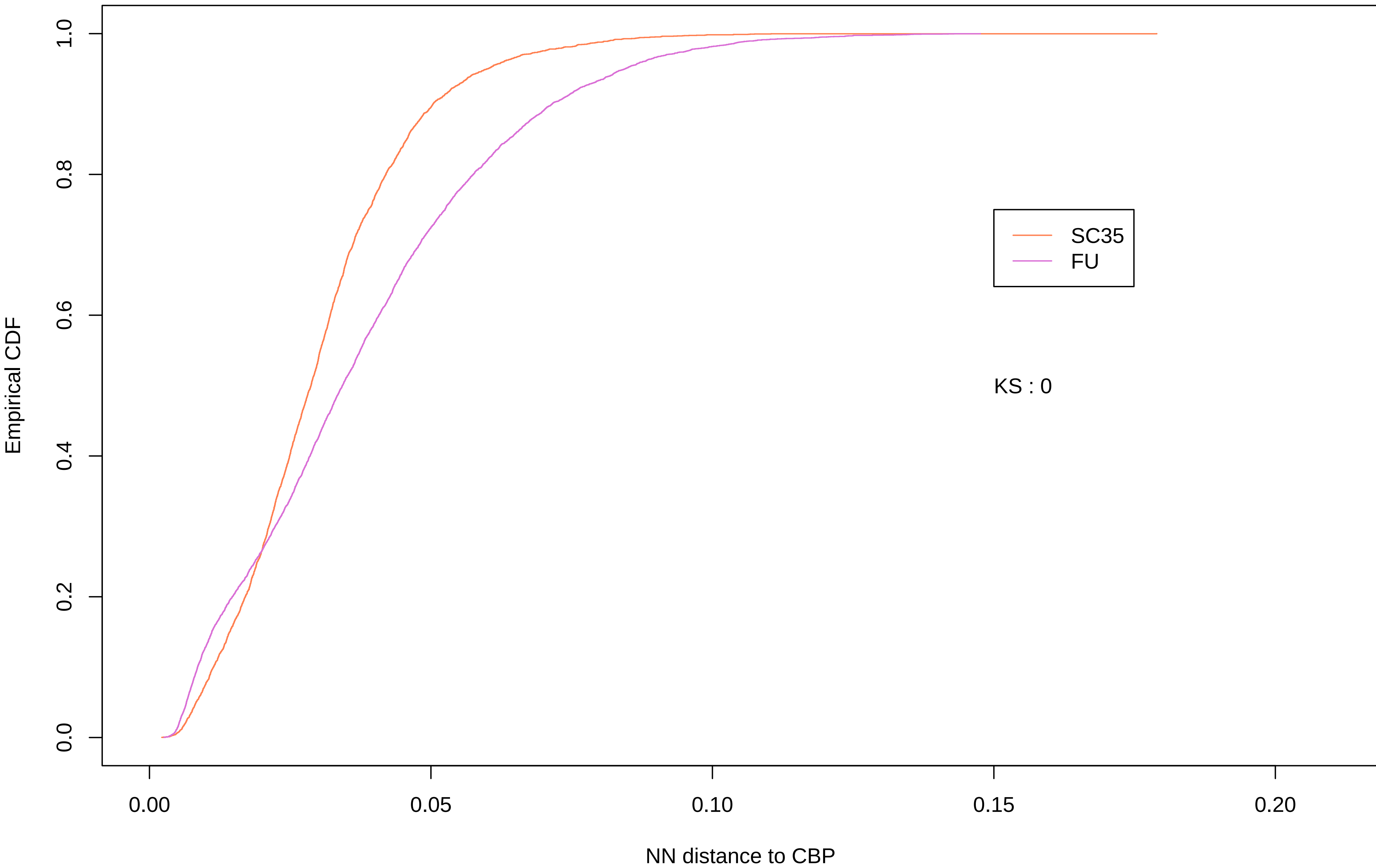

Empirical CDF
0.0
0.2
0.4
0.6
0.8
1.0
0.00
0.05
0.10
0.15
0.20
NN distance to CBP
SC35
FU
KS : 0

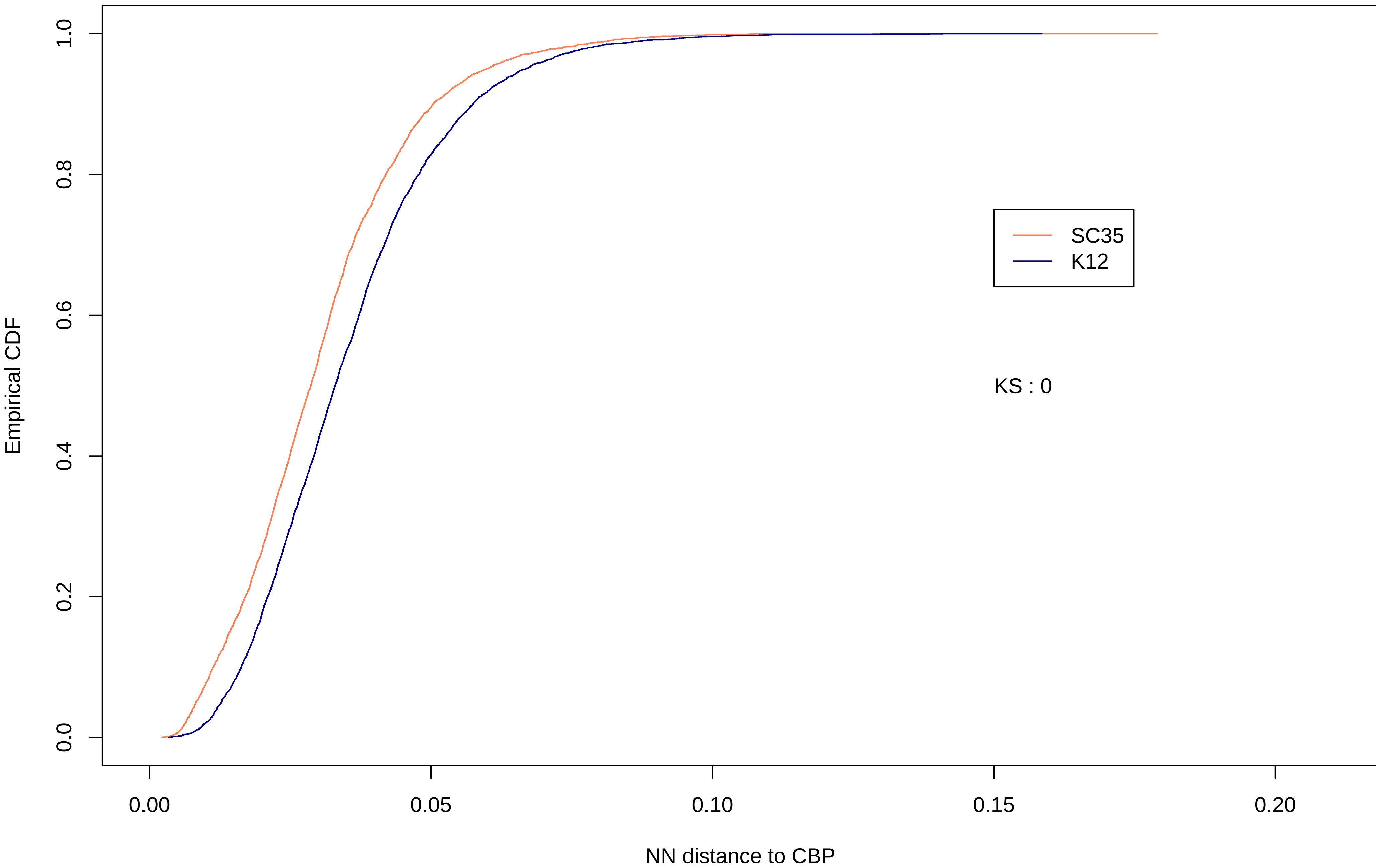

Empirical CDF
0.0
0.2
0.4
0.6
0.8
1.0
0.00
0.05
0.10
0.15
0.20
NN distance to CBP
SC35
K12
KS : 0

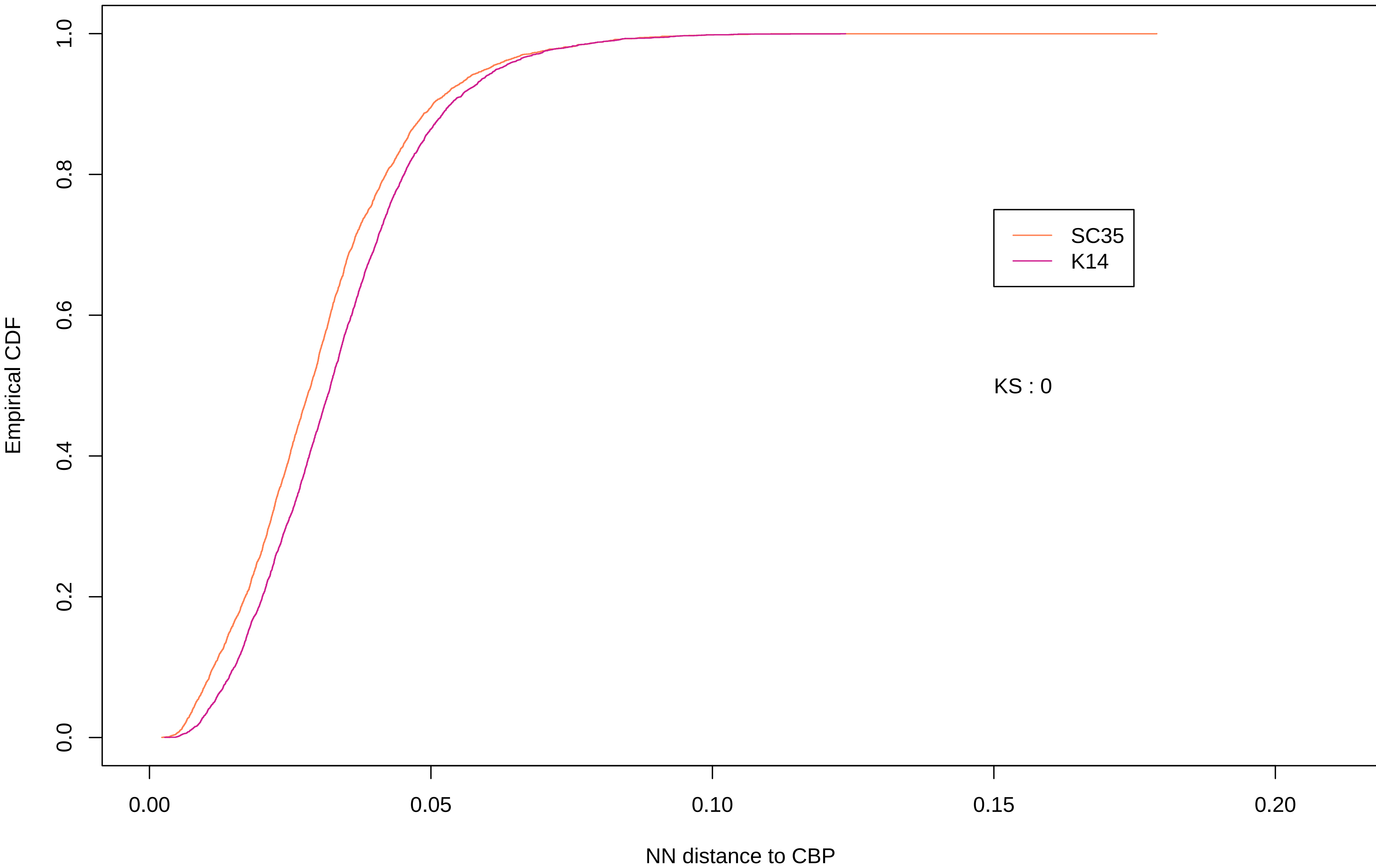
Empirical CDF
0.0
0.2
0.4
0.6
0.8
1.0
0.00
0.05
0.10
0.15
0.20
NN distance to CBP
SC35
K14
KS : 0

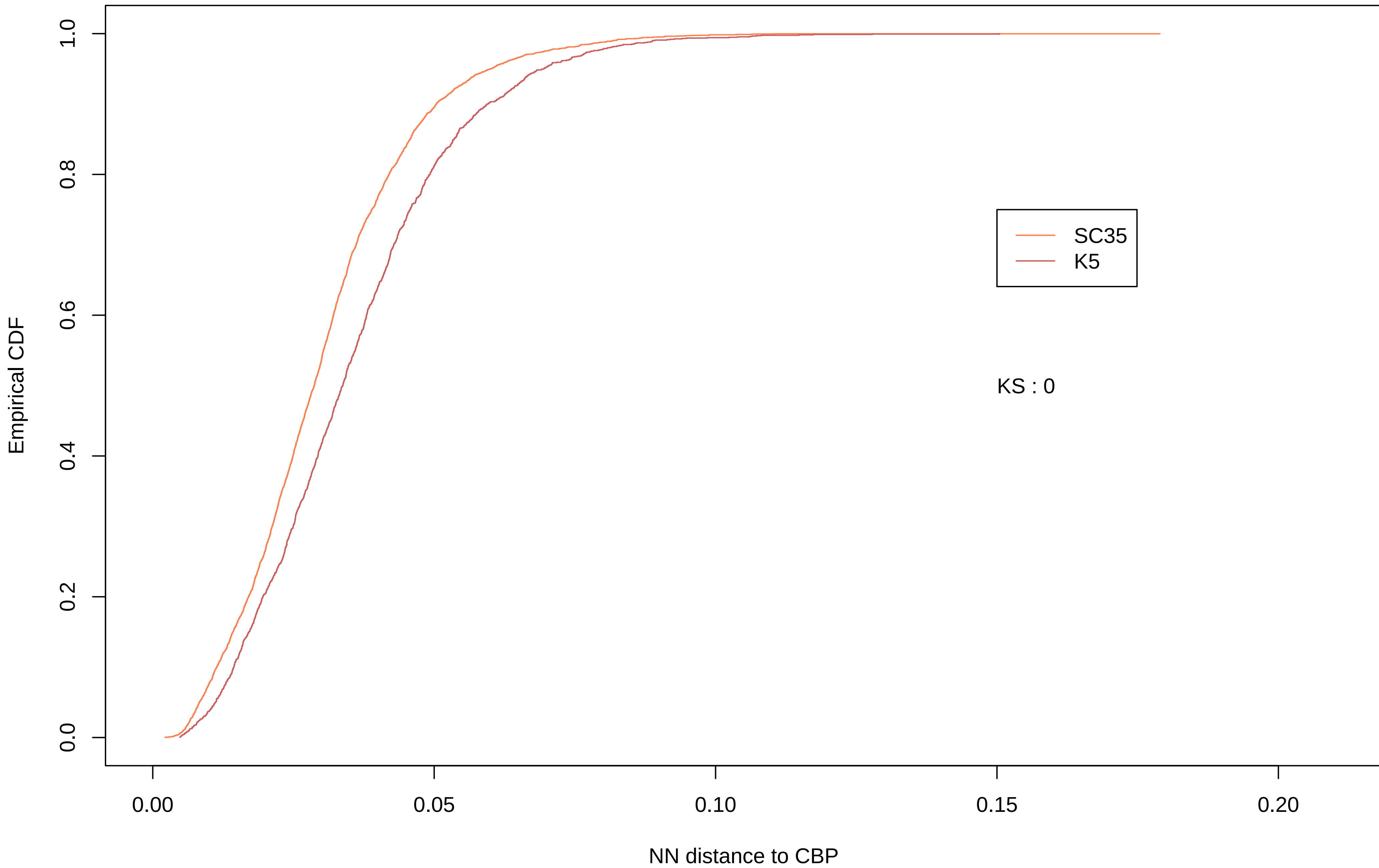

SC35
K5
KS : 0
Empirical CDF
0.0
0.2
0.4
0.6
0.8
1.0
0.00
0.05
0.10
0.15
0.20
NN distance to CBP

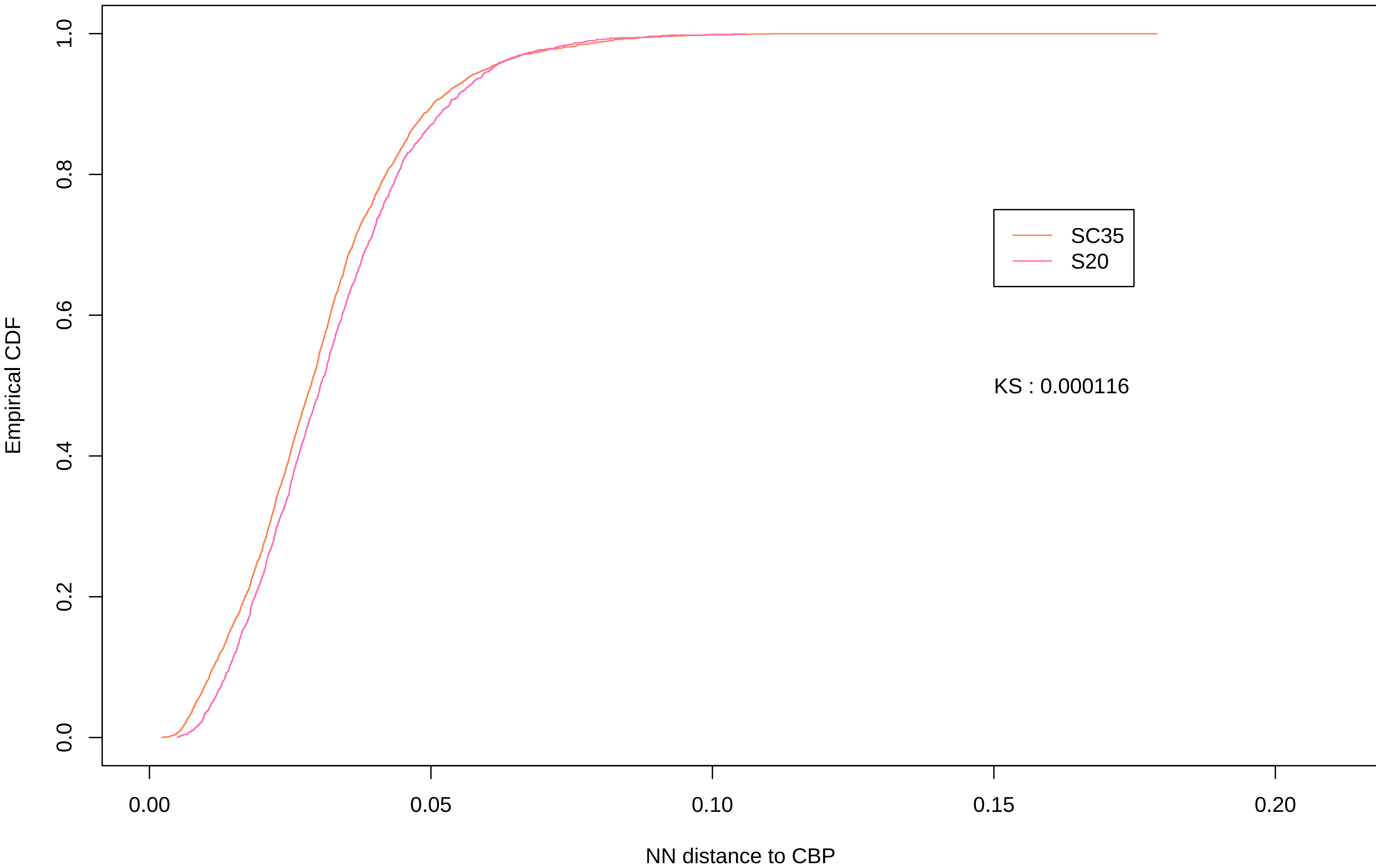

SC35
S20
KS : 0.000116
Empirical CDF
NN distance to CBP
0.0
0.2
0.4
0.6
0.8
1.0
0.00
0.05
0.10
0.15
0.20

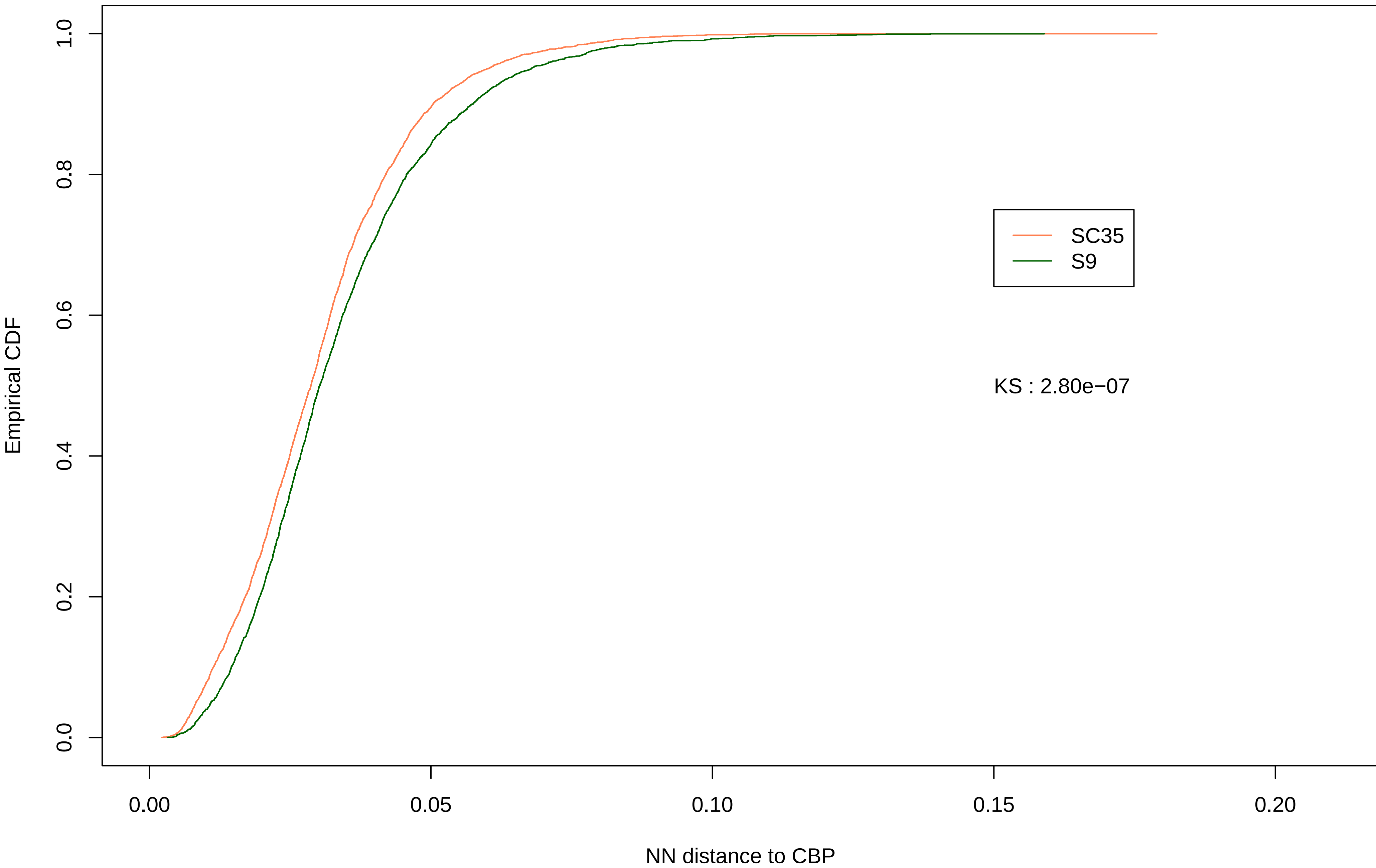

SC35
S9
KS : 2.80e−07
Empirical CDF
NN distance to CBP
0.0
0.2
0.4
0.6
0.8
1.0
0.00
0.05
0.10
0.15
0.20
